# Children’s views of obesity, body size and weight: systematic review of UK qualitative evidence

**DOI:** 10.1136/jech-2025-225045

**Published:** 2026-01-27

**Authors:** Theo Lorenc, Helen Burchett, Rosa Mendizabal-Espinosa, Claire Stansfield, Katy Sutcliffe, Amanda Sowden

**Affiliations:** 1 Centre for Reviews and Dissemination, University of York, York, England, UK; 2 Department of Public Health, Environments and Society, London School of Hygiene and Tropical Medicine, London, UK; 3 EPPI-Centre, Social Science Research Unit, University College London Institute of Education, London, UK

**Keywords:** OBESITY, CHILD, QUALITATIVE RESEARCH, SYSTEMATIC REVIEW

## Abstract

**Background:**

Understanding views about body size and weight is key to addressing both obesity and weight stigma. The views of younger children are not well understood and may differ in important ways from those of adults and young people. This review aimed to fill this gap.

**Methods:**

Systematic review of qualitative evidence. We searched 19 database sources in February 2025 and included qualitative studies from the UK published since 2008 reporting data on children’s (4–12 years) views of obesity, body size, body shape or weight. We assessed study quality using the Critical Appraisal Skills Programme checklist and conducted a thematic synthesis of the data.

**Results:**

We included 34 studies. Study quality overall was fairly high. Children reported a range of perceived impacts of body weight, including health impacts, limitations on activities, bullying and teasing, which generated negative perceptions of overweight and fear of fatness. Ideas about body shape may be influenced by family members and by media or social media content. Children identified diet and physical activity as the main influences on body weight. There may be differences in views between girls and boys at older ages, although both are concerned about weight. We found very little data on differences relating to ethnicity or socioeconomic status. Children with overweight or obesity reported a pervasive experience of negative attitudes and bullying, which could be a barrier to participating in activities.

**Conclusion:**

Children, including very young children, generally hold very negative views of overweight and obesity. Overweight is seen to be linked with unhealthiness, limited agency and with being bullied. These links may influence how children understand messages about weight and health.

**PROSPERO registration number:**

CRD42025650306.

**WHAT IS ALREADY KNOWN ON THIS TOPIC**

- Childhood obesity is a serious public health problem.
- Weight stigma and body dissatisfaction are widespread among young people and adults.

**WHAT THIS STUDY ADDS**

- Children generally have negative views of overweight, but understand it differently to adults.
- Overweight is seen as unhealthy and as limiting physical activity.
- Weight-based bullying is widespread.

**HOW THIS STUDY MIGHT AFFECT RESEARCH, PRACTICE OR POLICY**

- There is a risk that obesity prevention interventions could contribute to weight stigma.
- More attention is needed to how children’s views and concerns about weight differ from adults’, and how they shape the reception of public health messages.
- There is limited data on differences between groups of children.

## Background

 Overweight and obesity in children is a serious and growing public health concern. Data from the National Child Measurement Programme (NCMP) in England show a prevalence of obesity of 9.6% in the Reception year (age 4–5) and 22.1% in year 6 (age 10–11).^1^ The causes of obesity are complex, including genetic, behavioural, social and environmental factors. A wide range of interventions have been implemented to attempt to prevent childhood overweight, in schools, homes and community settings.^2^

Body dissatisfaction and disordered eating are also widespread among children^3 4^ and are increasingly recognised as a serious public health problem in their own right.^5^ Children frequently report weight stigma, bias against people with overweight and weight-based bullying or teasing.^6 7^ These social and interpersonal factors can have serious impacts on mental health and well-being and may also contribute to overweight.^8 9^

These interlinked problems pose a challenge for policy and practice. Qualitative data can help to address them by understanding how people make sense of body size and weight and the social and cultural narratives which influence their behaviours and attitudes. There is a particular need to understand the views of children themselves, as distinct from those of parents, teachers or other adults (or teenagers, whose views are likely to differ in key respects). For these reasons, we focused on data from children aged 4–12 years in the UK, looking broadly at views of obesity, weight and body size. This review builds on the previous review by Rees and colleagues published in 2009, which, to our knowledge, is the last systematic review to cover these data.^10^

## Methods

The review question was: What is known from qualitative studies from the UK about how children aged 4–12 perceive body size, shape or weight? The review protocol was registered on PROSPERO before starting work (CRD42025650306). EPPI-Reviewer 6 software was used to manage data.^11^

We searched the following database sources in January and February 2025:

- ASSIA (ProQuest).
- Book Citation Index—Social Science (Web of Science).
- British Education Index (EBSCO).
- CINAHL (EBSCO).
- Conference Proceeding Citation Index (Web of Science).
- EMBASE (OVID).
- ERIC (EBSCO).
- Health Management Information Consortium (OVID).
- International Bibliography of Social Sciences (ProQuest).
- MEDLINE (OVID).
- NSPCC online library catalogue.
- OATD.org.
- OpenDissertations (EBSCO).
- OpenAlex.
- PolicyCommons.
- ProQuest dissertations and Theses.
- PsycINFO (OVID).
- Social Policy and Practice (OVID).
- Social Science Citation Index and Emerging Sources Citation Index (Web of Science).
- Theses Collection Wales.

We also searched websites and academic search engines and conducted forward citation searching and reference list checking on all eligible references.

The database searches were structured around the following concepts:

1. Population: children and young people.
2. Phenomena: body size, body image, weight and height measurements, weight stigma, weight anxiety, weight satisfaction.
3. Research focus: views, experiences, qualitative research.
4. Country: UK.
5. Limits: publication date from 2008, English language.

The full search strategies are found in online supplemental appendix 1.

A random sample of 10% of titles and abstracts was screened by two reviewers independently and disagreements were resolved by discussion. Agreement on inclusion for this sample was 95.3%. The remaining titles and abstracts were screened by one reviewer alone. All full-text references were screened by two reviewers independently (references to studies excluded in full text are found in online supplemental appendix 4). The inclusion criteria were:

1. The study reports substantive primary qualitative data.
2. The study reports data from children or young people aged 4–12 (either more than 50% of the population were in this age range, or there were separately reported data on this age group).
3. The study reports substantive data about body size, shape, weight or obesity, and/or the experiences of children with overweight or obesity.
4. The study was published in 2008 or later.
5. The study was conducted in the UK.

We assessed the quality of included studies using the Critical Appraisal Skills Programme (CASP) checklist for qualitative research. We extracted data on contextual and methodological features of the studies. These tasks were carried out by one reviewer and checked in detail by a second reviewer. We extracted qualitative findings data using the line-by-line coding function in EPPI-Reviewer; only data corresponding to the inclusion criteria (age 4–12 and topics to do with weight or body size) were coded. The coding focused on direct quotes from participants, but we did also code study authors’ summaries and characterisations of participants’ views where these provided data over and above what could be extracted from direct quotes. We did not code study authors’ broader interpretations or theories. The full coding frame is found in online supplemental appendix 5. Data were synthesised using a thematic synthesis method based on grounded-theory principles.^12^ We developed codes inductively, recoding studies as new codes emerged. Finally, we developed third-order constructs based on overarching lines of argument across the evidence base. We used GRADE-CERQual to assess confidence in the findings.^13^

## Results

**Figure 1:**
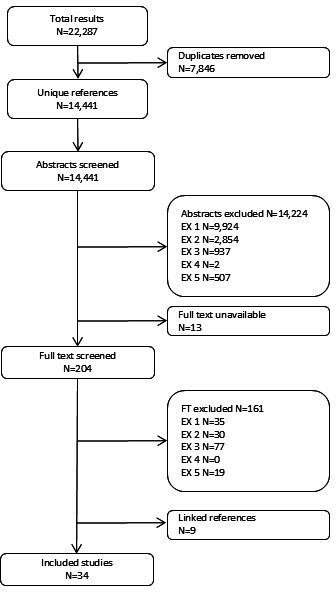
Flow of literature through the review.

The searches located 14 441 unique records. After screening, 34 studies (43 study reports) were included in the review. The flow of literature is shown in figure 1. Brief descriptive information on the characteristics of the studies is shown in table 1 (with further detail in online supplemental appendix 6). The studies represent a range of methodological approaches, with data collection including individual and group interviews, ‘draw and talk’ methods and participant observation. The topic foci of the studies were also diverse. Some focused on views of obesity or body size, while others looked at views of diet, physical activity or health in general (in the latter cases, we only extracted data on views of body size). Five studies mainly or wholly included children with overweight or obesity. Most studies included both girls and boys; seven studies focused mainly or wholly on girls, and none on boys. Seven studies reported that either the sampling frame or the sample was more than 50% black or minority ethnic, and 10 that they were of lower socioeconomic status (although this was often reported impressionistically).

**Table 1:** Descriptive characteristics of included studies

First author, reference(s)	Study focus	Age	% female	Other information on sample/context
Baxter^22 54^	Understandings of weight change	4–6	38	–
Bell^38^	Views of ‘fitspiration’ content on social media	12–13	35	Higher-SES school
Blood^41^	Experiences of school-based height and weight measurement	10–11	75	75% BME sample
Bromfield^26^	Implementation of National Healthy Schools Programme relating to obesity	9–11	NR	Lower-SES area
Charsley^23 55^	Perceptions of fatness and other physical differences	4–7	49	39% BME sample
Clark^35 56^	Girls’ experiences of sport and physical activity	10–13	100	–
Conway^44^	Views of food labelling and the NCMP	9–13	64%	61% BME, most lower-SES sample
Cowley^24^	Attitudes to PE and physical activity	12–13	44%	Medium-SES school
Dearing^27^	Pro-social behavioural intentions towards peers with obesity	4–6	35%	Mix of settings
Fairbrother^14^	Perceptions of food and relationship between food and health	9–10	NR	Mix of settings
Fielden^18^	Understandings of obesity and links to diet and physical activity	4–5, 10–11	NR	–
Gemmell^36^	Overweight children’s perceptions and experiences relating to body size	8–12	67%	All overweight
Gillison^33^	Development of guidance for parents on talking to children about weight	9–11	63%	–
Goldthorpe^40^	Views about responsibility for health	8–10	50%	Lower-SES area; 56% BME sample
Hall^31^	Experiences of children with overweight; experiences of weight-based victimisation	9–11	52%	Higher-SES area; mostly White sample
Harrold^57^	Obesity stigma	4–7	54%	Lower-SES area; mostly White sample
Herbert^39^	Views of weight monitoring	8–12	48%	Mixed-SES schools
Hooper^19^	Conceptions of health relating to physical education	11–12	NR	Mix of settings
Kamal^28^	Views about determinants of obesity and compensatory reasoning about health behaviours	5–10	55%	Higher-SES, mostly White school
Kesten^32^	Influences on girls’ obesity-related health behaviours	6–11	100%	Mix of settings
Kumari^15^	Views of young people with overweight	11–13	36%	54% BME sample. All overweight
Lewis^46^	Views of physical activity in children with overweight	6–16	50%	Lower-SES area. All overweight
Mansfield^16^	Views of childhood obesity	9–10	37%	Lower-SES area
Miller^42^	Views on prevention of eating disorders	10–14	100%	–
Monaghan^25^	Views of body size and health	12–13	100%	Most White
Murphy^30^	Views of health, diet, physical activity and weight	9–10	58%	73% BME, most lower-SES sample
Newson^17^	Experiences of children with obesity; views of a weight management programme	7–13	56%	All overweight
Nnyanzi^58 59^	Impact and implementation of the NCMP	10–11	67%	All white, most lower-SES sample
Ogden^60^	Choices about food	9–10	63%	All White sample
Paddock^45 61^	Use of social media	11–14	52%	Mostly White sample; lower-SES area
Palmer^20^	Understandings and experiences of the body	9–10	59%	Mix of settings
Rich^21 29 34 62^	(Varied aims)	9–16	NR	–
Willett^43^	Girls’ views about fashion and digital media	12–13	100%	Lower-SES, 75% BME school
Windram-Geddes^37 63^	Girls’ experiences of PE and physical activity	10–14	100%	–

BME, black and minority ethnic; NCMP, National Child Measurement Programme; NR, not reported; PE, physical activity; SEN, special educational needs; SES, socioeconomic status.

The results of quality assessment are presented in online supplemental appendix 2. Overall, the quality of the studies was reasonably high. The most common domains in which there were limitations were sampling and recruitment, and the relationship of researcher to participants. Further information can be found in the GRADE-Cerqual assessment in online supplemental appendix 3.

The findings are structured into three main areas: perceived impacts of weight, perceived causes of overweight and personal experiences and goals related to weight and size. In addition, findings from children experiencing overweight or obesity, and differences between groups of children, are explored.

### Perceived impacts of weight

Participants identified issues relating to the health impacts of overweight. In some cases, these were specific outcomes such as heart disease and diabetes,^14^,^17^ particularly in studies of children with overweight or obesity.^15 17^ Some participants also recalled media content on people with extreme obesity and serious health problems.^14 18^ More often, however, overweight was seen as ‘unhealthy’ in a general sense.^18^,^21^ Studies of younger children (4–6 years) found that they already saw overweight as harmful to health, or even fatal in some cases, without having any detailed understanding of the pathways involved^22 23^: ‘he might explode’.^22^ Concerns about appearance were also mentioned, but almost always linked to health^17^,^19^; conversely, health was often seen as directly visible in people’s appearance.^19 21^ These findings were coherent across the studies (and between children with and without overweight), and there are minimal concerns about study quality.

If you look at a person, and they’re bigger, well, fatter … you can just tell that they’re not healthy.^19^

Because it’s bad for you, because it looks bad.^18^

Some participants also identified negative health impacts from underweight, using terms like ‘weak’ or ‘fragile’, but these were mentioned less often and seen as less serious than the consequences of overweight.^14 18 19 22 24 25^ These findings were fairly sparse and reflect a range of different perceptions.

Participants felt that overweight could be a barrier to doing things and participating in activities, such as physical play, sports and everyday activities such as walking.^1420 22 23 26^,^29^ Some felt that overweight could limit social relationships by restricting activities and reducing mobility.^16 30^ Limitations on activity were mentioned by several younger children,^22 23 27^ with one study finding this was the most common reason for not wanting to be friends with a child with overweight.^23^ These findings were coherent across studies and there are minimal concerns about study quality.

Because she’s too fat and when you’re fat you can’t do exercise … because if you’re too wide then you find it difficult to walk …^28^

He do not like nothing because he’s too big, he cannot sit nowhere, he has to sit on the floor. He’s bored, coz got no friends … he cannot get anywhere because he’s too fat, so he has to go in his taxi car and things.^30^

The idea of limitations on physical activity sometimes led to a broader sense that overweight compromises the ability to live a ‘normal’ life^20 22 29^ and could be a barrier to employment in adulthood.^14 16 18^

[Of character gaining weight] She won’t be able to do normal things.^22^

Because I don’t want to be fat … it’s disgusting […] You can’t even walk properly.^20^

Cause if you’re too fat you could not be really ﬁt and walk around as much as you normally do […] I wouldn’t really like it ’cause I would just like to be normal like everyone else.^29^

Children with overweight were thought to often experience bullying and teasing.^1416 19 22^,^24 30 31^ (As discussed below, this perception was confirmed by children with overweight themselves.) This perception was shared by younger as well as older children^22 23^ and seems to have often been a focus of concern. Some felt that overweight could lead to longer-term difficulties in forming friendships.^19 23 31^ Participants suggested that weight-based bullying could itself have impacts on health, through disordered eating or a lack of motivation to be physically active.^24 30^ These findings were coherent across studies and there are minimal concerns about study quality.

### Perceived causes of overweight

The main causes of overweight, or weight gain or loss, identified by participants were diet and physical activity. Overweight was ascribed to overeating in general by some participants.^15 23^ More frequently, children (especially younger children) blamed specific ‘bad’ foods such as sweets, chips or ‘junk’, as contrasted with ‘good’ foods like fruit and vegetables.^22 28 30 32^ Some older children referred to macronutrients, most commonly fat and sugar^14 15 33^ or carbohydrates.^34^ The idea of an energy balance between diet and physical activity was expressed by several participants, sometimes as a deliberate strategy to avoid weight gain.^3235^,^37^ Some saw overeating in psychological terms as an ‘addiction’ or a ‘brain problem’.^14 16 18 34^ While there are concerns about study quality around these findings, they are consistent across studies.

Many participants identified a lack of physical activity as an important cause of overweight and increasing activity as a route to weight loss.^1416 18 19 22 28 32^,^39^ The link here is often unclear—although one study does go into children’s theories, finding a diverse set of ideas about how exercise ‘grinds down’ food or stops fat ‘sticking’ to the body^28^—and may not be clearly distinguished from a broader link between lower weight, fitness and health. There are also some concerns about study quality around these findings. In some cases, this link is seen in terms of people with overweight being ‘lazy’.^19 22 28^

Because they’re really thin and healthy … because, the fat … when you run you get thinner, what means you’re healthier, what means you can tell.^28^

A few participants also referred to natural or familial predispositions to overweight.^14 21^ Social and environmental factors were rarely mentioned: one study focusing on questions of responsibility found diverse views, with some participants citing social factors such as availability of fast food.^40^ Several also mentioned the influence of parents on behaviour.^14^,^16^

### Personal experiences and goals related to weight

Aside from these broader perceptions, participants’ specific goals for themselves were varied. Some, mostly girls, reported a desire to lose weight^20 32 35 37^; boys were more likely to focus on strength or muscularity.^14 20 24^ Studies eliciting preferences for body size, mostly in older girls, tended to find that the ideal was thin but not excessively thin.^21 34 37^ There are some concerns about study quality and potential bias around these findings, and relatively few participants express explicit body ideals as such.

I want to stay like quite skinny but not like too skinny like size zero or anything like that. I think it would be good to be just like a normal size.^21^

Participants in several studies reported trying to lose weight.^14 32 35 41^ Some reported potentially disordered eating behaviours such as skipping meals or excessive exercising^35 37 41^ or referred to peers who had developed disordered eating patterns.^21 42 43^ There are some concerns about study quality and consistency around these findings.

Several studies discussed the role of media content in shaping body images, both social and online media,^3238 39 43^,^45^ and traditional media such as television and magazines.^15 21 32 39 42 43^ Participants in several studies mentioned sensationalistic media content on people whose lives had been impacted by extreme obesity.^14^,^1629 31^ There are some concerns about study quality around these findings, and some disparities between in-depth reports of participants’ experiences and their general perceptions. Participants were frequently critical of the unrealistic body standards presented in media and social media and the promotion of unhealthy eating behaviours and body dissatisfaction.

Cos if you see in the media that someone is completely opposite to you and only people who are completely opposite to you in the media, then that’s gonna have a downside, but that might cause them to comfort eat and become more obese.^15^

‘Fat talk’ from other people, including parents and teachers, was a frequent theme. Several participants reported that their parents (usually mothers) commented on their weight or encouraged them to lose weight,^20 32 35^ or in one case to gain weight.^14^ More generally, many picked up on parents’ and family members’ dissatisfaction with their own weight and unsuccessful attempts at weight loss.^14 17 18 39^ While there are concerns about study quality around these findings, they are consistent across studies.

### Experiences of children with overweight or obesity

Studies on children with overweight or obesity were analysed as a separate group.^15 17 31 36 46^ Children with overweight described mixed attitudes towards their own body shape, with some having strongly negative self-perceptions and others feeling they were only slightly overweight.^17 36^ Many described weight-based bullying and teasing from peers, which was sometimes serious and long-lasting.^15 17 31 36^ While individuals’ responses varied, bullying was widely seen as pervasive and unavoidable and could have serious impacts on social relationships and mental health.^15 17 36^

I was bullied at one point, which I think maybe, almost every person goes to a school may have experienced at one point. It’s pretty much … you can’t avoid it forever. You’ll get bullied at one point.^15^

Some participants described physical limitations on participating in activities.^15 17^ However, this was a less commonly described barrier to activity than negative reactions from others,^17 36^ and physical education in particular could be an occasion for bullying.^15 35^

I definitely won't be going to any other clubs after school. I just will never fit in with them sorts.^17^

There are some concerns about study quality around these findings; in particular, all these studies recruited from weight management interventions and may not be representative of all children with overweight or obesity.

### Differences between groups

We located limited data on differences between groups of children. On gender, as mentioned above, some data indicate differences between boys and girls in preferred body shape. There is an assumption in the literature that girls are generally more concerned about weight—reflected in the fact that seven studies focused on girls^25 29 32 35 37 42 43^ and none on boys—which is rarely tested directly. A few male participants suggested that girls cared more about weight because they wanted to look like models or celebrities.^18 45^ Gender norms may affect willingness to talk about weight-related issues, with some participants suggesting that boys were as likely to be emotionally affected by weight-based teasing as girls, but would try to hide the fact.^26^ Very few data addressed differences by ethnicity or socioeconomic status.

## Discussion

The findings of this review indicate that many children hold very negative views of overweight and obesity. These views appear to be well established at early ages, with children as young as four expressing negative beliefs about people with overweight.

Our findings indicate three main drivers of these beliefs. First, there is a widespread sense that overweight is unhealthy. In some cases, this is informed by awareness of specific health consequences, but this is not generally the case. Rather, thinness (but not extreme thinness), health, fitness and attractiveness form a constellation of imagery and affective responses. These associations may form an important driver of stigmatisation of overweight, more so than concerns about appearance as such, which when mentioned in the data was often associated with health.

Second, there is a perception that overweight is a limitation on what one can do. This not only relates primarily to physical activities such as active play and sport but also draws in a broader sense that overweight is a barrier to having a ‘normal’ life and friendships. These views largely do not seem to be based on direct experience; while they are pervasive among children without overweight, they are much less apparent in children with overweight. Third, there is a concern that overweight will lead to bullying and social exclusion, which often forms a focus of fear of becoming overweight. This theme is strongly confirmed by the data from children with overweight, which show that bullying and teasing are pervasive and often serious.

The findings suggest that the views of children in this age group may differ in important ways from those of young people aged over 12 and adults (and there are differences between older and younger children in our age range, although also continuities). Concerns about appearance and body image may be less important than sometimes suggested, although they become more manifest towards the older end of our age range, along with more distinct gender norms. Pseudo-moral stereotypes of people with overweight as ‘lazy’ or ‘greedy’, while sometimes mentioned, also do not appear to be central. Views of people with overweight as unhealthy, and as limited in their physical agency, may be at least as important in shaping negative views, particularly for younger children. On the other hand, some older children express nuanced and critical views towards messages about weight and may have a sophisticated understanding of the causes of overweight.

Compared with Rees *et al*’s review,^10^ there are some clear continuities in the findings, including the prevalence of bullying and teasing, generally negative views of overweight and views on causes of weight and on preferred body sizes. However, the link between weight and health is less prominent in the 2009 review, while, as noted, it is a prominent feature of our analysis (and mentioned by several primary study authors); this may suggest that this theme has become more important in recent years. Compared with studies on older age groups, our findings show some commonalities, such as the perceived impact of overweight on activities and social relationships^47^ and the risk of a self-reinforcing cycle of overweight, bullying and limited physical activity.^48^ While we excluded literature from countries other than the UK, several of the findings align with qualitative research from other countries, particularly the link between weight, health and physical ability.^49^,^51^ However, there may also be divergences, including different body ideals among some populations, such as black children in the USA.^52^

The findings suggest some implications for policy and practice. There may sometimes be well-grounded concerns about public health communication on weight and obesity inadvertently reinforcing weight stigma. Ideas about weight bring with them a network of connotations and imagery, which could undermine the intended content of messages about obesity and health behaviours. The findings also suggest some reasons why weight-monitoring programmes such as the NCMP have been controversial: if weight forms an emotionally charged focus for a range of other concerns—health, agency, social relationships and so on—then responses to the ‘objective’ assessment of weight or BMI may often be unpredictable.

While this review was fully systematic in its methods, there may be some limitations. It did not cover several related topics which would be potentially illuminating, including views of food or physical activity, or concepts of health in general (except where they overlapped with our topic focus). We also excluded data from countries other than the UK, to ensure relevance to the policy context for this review. We used the CASP critical appraisal tool; this tool, and other checklist-based approaches, has been criticised for not giving a full picture of rigour in qualitative research.^53^ We did not fully integrate critical appraisal into the synthesis process, relying instead on GRADE-Cerqual to assess confidence in the findings. We did not independently double-code findings data. There may also be some limitations in the primary studies. Sampling and recruitment bias is a particular concern. Two studies reported differential response rates by gender as a result of parents withholding consent at higher rates for girls,^22 27^ and there may be other sources of recruitment bias, due to the sensitivity of the topic, which were not reported in the studies.

The findings indicate that negative views of weight and weight-based bullying are real concerns among this age group, including very young children. Messages about the causes and health impacts of overweight have considerable impact but may be understood in unpredictable ways; more attention is needed to understand where children learn these links, and how this differs between groups. Children’s understandings of weight differ in important ways from those of teenagers and adults and should not be assumed to reflect the same concerns and priorities.

## Supplementary material

10.1136/jech-2025-225045 online supplemental file 1

10.1136/jech-2025-225045 online supplemental file 2

10.1136/jech-2025-225045 online supplemental file 3

10.1136/jech-2025-225045 online supplemental file 4

10.1136/jech-2025-225045 online supplemental file 5

10.1136/jech-2025-225045 online supplemental file 6

## Data Availability

Data are available upon reasonable request.
