# Supplementary material for: Children’s views of obesity, body size and weight: systematic review of UK qualitative evidence
Source: J Epidemiol Community Health. 2026 Jan 27;80(6):e225045. doi: 10.1136/jech-2025-225045 (PMC13217130; doi:10.1136/jech-2025-225045)
Supplement: online supplemental file 1 [file jech-80-6-s001.docx]

***Children’s views of obesity, body size and weight: Systematic review of UK qualitative evidence. Appendix***

**Search strategies**

| Source name | **MEDLINE** |
| --- | --- |
| Database name/platform | **MEDLINE (OVID)** |
| Date of search | 31 Jan 2025 |
| Date of import | 31 Jan 2025 |
| Number items | 2264 |
| Duplicates | 10 |
| Description |  |
| Notes |  |
| Search string | ________________________________________ Database: Ovid MEDLINE(R) ALL <1946 to January 30, 2025> Search Strategy: 1 ("young people*" or adolescent* or youth or pubertal or pubescent or "pre adolescent" or "Pre pubescent" or "pre pubertal" or teen* or preteen* or tweens or tweenage* or youth or youths or schoolboy* or schoolgirl* or "school aged" or "young person*" or juvenile* or "Boy" or "boys" or "child" or "children*" or "child's" or "Girl" or "girls" or "Minors" or "preadolescent" or "Prepubescent" or "schoolchild*" or pubescent or "early adolescent*").ti,ab,kf,ot,jw. (2192224) 2 exp Minors/ or exp child/ or exp Adolescent/ (3509687) 3 ((child* or adolescence*) not childbirth*).jw. (198483) 4 1 or 2 or 3 (4215243) 5 (Weight not "birth weight").ti. (109030) 6 ("anti-fat" or bodyweight or "obese" or "obesity" or "overweight" or "skinny" or "fatness" or "thinness" or "body image" or "body hatred" or "body positive" or "thin ideal*" or "fat ideal*").ti,ab,kf,ot. (474406) 7 ("body esteem" and ("weight" or height or fat or thin)).ti,ab,kf,ot. (278) 8 ((body adj2 ideal*) or (body adj2 shape) or (body adj2 shapes) or (body adj1 size)).ti,ab,kf,ot. (36277) 9 ((body adj10 weight) not (body adj10 "birth weight")).ti,ab,kf,ot. (295776) 10 ("underweight" not "birth weight").ti,ab,kf,ot. (15908) 11 ((weight adj1 bias) or (weight adj1 biases)).ti,ab,kf,ot. (825) 12 ("weight change" or "weight changes" or (weight adj2 gain*) or (weight adj2 loss) or "lose weight" or "losing weight" or (weight adj1 (reduc* or manage* or control*))).ti,ab,kf,ot. (236658) 13 ("weight adj1 measurement*" or "height adj1 measurement*").ti,ab,kf,ot. (0) 14 ("healthy weight" or "unhealthy weight" or "weight adj5 monitor*" or "height adj5 monitor*").ti,ab,kf,ot. (4949) 15 ("being measured" and ("weight" or weighing or height)).ti,ab,kf,ot. (139) 16 (weighing adj3 experiences).ti,ab,kf,ot. (7) 17 (weight screening* or height screening*).ti,ab,kf,ot. (67) 18 ("being fat" or "being weighed" or "Being thin" or "Being tall" or "Being short").ti,ab,kf,ot. (690) 19 ((stereotype or stereotypes or Stigma or stigmati* or ideals or ideal or discriminate* or discrimination or "peer pressure" or prejudice* or bully* or teasing or cyberbull* or bullied) adj5 ("fat" or weight or thin or height or tall or short or muscle* or muscular* or body)).ti,ab,kf,ot. (12664) 20 ((fat or weight or thin or body or height or muscle or muscular*) adj5 (acceptability or satisfaction or satisfied or dissatisfaction or dissatisfied or anxiety or anxious or angst or peer pressure or feelings or worries or worry or concern or concerns or "over concern" or emotions or emotion or concerns or "self-worth" or "self esteem" or esteem or "self-concept" or "self-identification" or "pre-occupation" or "preoccupation" or "acceptability" or perceiv* or understand* or perception* or internalis* or attitud*)).ti,ab,kf,ot. (41040) 21 (Body adj1 (dysmorph* or dismorph*)).ti,ab,kf,ot. (1717) 22 (Muscle adj1 (dysmorph* or dismorph*)).ti,ab,kf,ot. (291) 23 ((body or fat or thin or height or weight) adj3 (shame or phobia*)).ti,ab,kf,ot. (580) 24 ((weight adj2 muscle) or "excess weight").ti,ab,kf,ot. (11054) 25 ((weight adj3 appearance) or (height adj3 appearance)).ti,ab,kf,ot. (907) 26 5 or 6 or 7 or 8 or 9 or 10 or 11 or 12 or 13 or 14 or 15 or 16 or 17 or 18 or 19 or 20 or 21 or 22 or 23 or 24 or 25 (924493) 27 Body Dysmorphic Disorders/ or Weight Prejudice/ or "body size"/ or "body weight"/ or exp "ideal body weight"/ or "obesity"/ or "overweight"/ or exp "thinness"/ (454755) 28 body height/ (38739) 29 27 or 28 (473493) 30 26 or 29 (1089571) 31 4 and 30 (208890) 32 "attitude to health"/ or "comprehension"/ or "ethnopsychology"/ or "Focus Groups"/ or "Interviews as Topic"/ or Qualitative Research/ or "attitude"/ or Personal Narrative/ (327649) 33 ((Girls or Boys or Child* or teen* or adolescen* or "young people*" or schoolchild* or pupil* or student*) adj2 ("perspective*" or "experience*" or "lived experience*" or "attitude" or "attitudes" or "attitudinal" or "belief" or "beliefs" or "discourse" or "discourses" or "life-world" or "opinions" or "standpoint" or "standpoints" or "understanding" or "understandings" or "viewpoint" or "viewpoints" or "views" or "voice" or "voices" or discussed or described* or drawings or drew)).ab. (74275) 34 ("audiorecording" or "ethnograph*" or "ethnolog*" or "ethnopsycholog*" or "ethno psycholog*" or "Focus Groups" or "Focus Group" or "repertory grid*" or "stories" or "audio record" or "audio recorded" or "audio recorder" or "audio recording" or "audio recordings" or "audio records" or "thematic analysis" or "phenomenol*" or "grounded theory" or "grounded studies" or "grounded research" or "purposive sampling" or "biographical method" or "theoretical sampl*" or "conversation analysis" or "theoretical saturation" or "thematic analyses" or "mixed design" or "mixed method" or "mixed methods" or "qualitative" or "interviewed" or "interviewing" or "interviewer" or "interviews" or "interview" or "narratives" or "Diary entries" or "diary study" or Diaries or journalling or (Questions* adj5 (semi-structured, or in-depth or open or "open ended")) or "Group discussion*").ti,ab,kf,ot. (864508) 35 ("experiences" or "lived experience*" or "perceptions" or "perspective" or "perspectives" or "experience" or "attitude" or "attitudes" or "attitudinal" or "belief" or "beliefs" or "discourse" or "discourses" or "life-world" or "opinions" or "perceived" or "perception" or "standpoint" or "standpoints" or "understanding" or "understandings" or "viewpoint" or "viewpoints" or "views" or "voice" or "voices" or talk*).ti,ot,kf. (952566) 36 32 or 33 or 34 or 35 (1812166) 37 4 and 30 and 36 (20780) 38 exp United Kingdom/ (402174) 39 (England not "New England").ti,ab,kf,ot,in,jw. (160806) 40 ("district council" or "local council" or "local authorities" or "NHS Trust" or "primary care trust" or "borough council" or "county council" or "local authority" or "district councils" or "local councils" or "NHS Trusts" or "primary care trusts" or "borough councils" or "county councils" or "Social Care Trust").ti,ab,ot,kf,in. (111776) 41 ("United Kingdom" or UK or "U.K." or Britain or GB or (British not "British Columbia")).ti,ab,ot,kf,in,jw. (2487990) 42 (Welsh or Scottish or "northern Irish" or Wales or Scotland or "northern Ireland").ti,ab,ot,kf,in,jw. (319223) 43 (national health service* or nhs*).ti,ab,in. (307965) 44 (english not ((published or publication* or translat* or written or language* or speak* or literature or citation*) adj5 english)).ti,ab. (139395) 45 (bath or "bath's" or ((birmingham not alabama*) or ("birmingham's" not alabama*) or bradford or "bradford's" or brighton or "brighton's" or bristol or "bristol's" or carlisle* or "carlisle's" or (cambridge not (massachusetts* or boston* or harvard*)) or ("cambridge's" not (massachusetts* or boston* or harvard*)) or (canterbury not zealand*) or ("canterbury's" not zealand*) or chelmsford or "chelmsford's" or chester or "chester's" or chichester or "chichester's" or coventry or "coventry's" or derby or "derby's" or (durham not (carolina* or nc)) or ("durham's" not (carolina* or nc)) or ely or "ely's" or exeter or "exeter's" or gloucester or "gloucester's" or hereford or "hereford's" or hull or "hull's" or lancaster or "lancaster's" or leeds* or leicester or "leicester's" or (lincoln not nebraska*) or ("lincoln's" not nebraska*) or (liverpool not (new south wales* or nsw)) or ("liverpool's" not (new south wales* or nsw)) or ((london not (ontario* or ont or toronto*)) or ("london's" not (ontario* or ont or toronto*)) or manchester or "manchester's" or (newcastle not (new south wales* or nsw)) or ("newcastle's" not (new south wales* or nsw)) or norwich or "norwich's" or nottingham or "nottingham's" or oxford or "oxford's" or peterborough or "peterborough's" or plymouth or "plymouth's" or portsmouth or "portsmouth's" or preston or "preston's" or ripon or "ripon's" or salford or "salford's" or salisbury or "salisbury's" or sheffield or "sheffield's" or southampton or "southampton's" or st albans or stoke or "stoke's" or sunderland or "sunderland's" or truro or "truro's" or wakefield or "wakefield's" or wells or westminster or "westminster's" or winchester or "winchester's" or wolverhampton or "wolverhampton's" or (worcester not (massachusetts* or boston* or harvard*)) or ("worcester's" not (massachusetts* or boston* or harvard*)) or (york not ("new york*" or ny or ontario* or ont or toronto*)) or ("york's" not ("new york*" or ny or ontario* or ont or toronto*))))).ti,ab,in. (1914200) 46 (aberdeen or "aberdeen's" or dundee or "dundee's" or edinburgh or "edinburgh's" or glasgow or "glasgow's" or inverness or (perth not australia*) or ("perth's" not australia*) or stirling or "stirling's").ti,ab,in. (281610) 47 (armagh or "armagh's" or belfast or "belfast's" or lisburn or "lisburn's" or londonderry or "londonderry's" or derry or "derry's" or newry or "newry's").ti,ab,in. (37724) 48 ("South Holland" or Aldershot or Ashfield or Barking or Barnet or Barnsley or Bedfordshire or Bexley or Birkenhead or Blackburn or Blackpool or Bolton or Bournemouth or Brent or Bridgend or Bromley or Bromwich or Buckinghamshire or Burnley or Camberwell or Cambridgeshire or Camden or Chelsea or Chelsea or Cheshire or Cleveland or Colchester or Cornwall or Crawley or Croydon or Cumbria or Dagenham or Dartford or Derbyshire or Devon or Doncaster or Dorset or Dudley or Ealing or Ealing or Eastbourne or Enfield or Essex or Farnborough or Fulham or Furness or Galloway or Gateshead or Glamorgan or Glasgow or Gloucestershire or Gravesham or Greenwich or Grimsby or Guildford or Hackney or Hamlets or Hammersmith or Hampshire or Haringey or Haringey or Harrow or Hartlepool or Harwell or Hastings or Havering or Helens or Hertfordshire or Highland or Hillingdon or Hounslow or Hounslow or Hove or Huddersfield or Humber or Ipswich or Islington or Kensington or Kent or Kingston or Kirklees or Knowsley or Lambeth or Lancashire or Leicestershire or Lewisham or Lichfield or Lincoln or Lincolnshire or Loughborough or Luton or Lynn or Mansfield or Merseyside or Merton or Middlesbrough or Midlands or Milton Keynes or Newcastle or Newham or Norfolk or Northampton or Northamptonshire or Northumberland or Nottinghamshire or Oadby or Oldham or Oxfordshire or Poole or Portsmouth or Reading or Redbridge or Redcar or Richmond or Rochdale or Rotherham or Rushmoor or Sandwell or Scarborough or Scilly or Shropshire or Slough or Solihull or Somerset or Southampton or Southend or Southwark or Staffordshire or Stockport or Stockton or Suffolk or Surrey or Sussex or Sutton or Swindon or Teesside or Telford or Thurrock or Tower Hamlets or Tyne or Tyneside or Walsall or Waltham or Wandsworth or Warrington or Warwickshire or Watford or Wigan or Wight or Wigston or Wiltshire or Wirral or Woking or Worcestershire or Worthing or Yorkshire).ti,ab,kf,ot,in. (1301438) 49 ("Isle of Man" or "Channel Islands" or "Guernsey").ti,ab,kf,ot,in. (1477) 50 (bangor or "bangor's" or cardiff or "cardiff's" or newport or "newport's" or "st asaph" or "st asaph's" or "st davids" or swansea or "swansea's").ti,ab,in. (77715) 51 38 or 39 or 40 or 41 or 42 or 43 or 44 or 45 or 46 or 47 or 48 or 49 or 50 (4089151) 52 (exp africa/ or exp americas/ or exp antarctic regions/ or exp arctic regions/ or exp asia/ or exp australia/ or exp oceania/ or developing countries/) not (exp United Kingdom/ or europe/) (3559746) 53 51 not 52 (3762338) 54 37 and 53 (2883) 55 limit 54 to yr="2008 -Current" (2264) |

| Source name | **EMBASE** |
| --- | --- |
| Database name/platform | **EMBASE (OVID)** |
| Date of search | Jan 31, 2025 |
| Date of import | Jan 31, 2025 |
| Number items | 2885 |
| Duplicates | 1233 |
| Description |  |
| Notes |  |
| Search string | ________________________________________ Database: Embase Classic+Embase <1947 to 2025 January 30> Search Strategy: 1 ("young people*" or adolescent* or youth or pubertal or pubescent or "pre adolescent" or "Pre pubescent" or "pre pubertal" or teen* or preteen* or tweens or tweenage* or youth or youths or schoolboy* or schoolgirl* or "school aged" or "young person*" or juvenile* or "Boy" or "boys" or "child" or "children*" or "child's" or "Girl" or "girls" or "Minors" or "preadolescent" or "Prepubescent" or "schoolchild*" or pubescent or "early adolescent*").ti,ab,kf,ot,jw. (2981056) 2 exp Minors/ or exp child/ or exp Adolescent/ (4732875) 3 ((child* or adolescence*) not childbirth*).jw. (266257) 4 1 or 2 or 3 (5424800) 5 (Weight not "birth weight").ti. (142755) 6 ("anti-fat" or bodyweight or "obese" or "obesity" or "overweight" or "skinny" or "fatness" or "thinness" or "body image" or "body hatred" or "body positive" or "thin ideal*" or "fat ideal*").ti,ab,kf,ot. (709401) 7 ("body esteem" and ("weight" or height or fat or thin)).ti,ab,kf,ot. (340) 8 ((body adj2 ideal*) or (body adj2 shape) or (body adj2 shapes) or (body adj1 size)).ti,ab,kf,ot. (42125) 9 ((body adj10 weight) not (body adj10 "birth weight")).ti,ab,kf,ot. (419931) 10 ("underweight" not "birth weight").ti,ab,kf,ot. (24473) 11 ((weight adj1 bias) or (weight adj1 biases)).ti,ab,kf,ot. (974) 12 ("weight change" or "weight changes" or (weight adj2 gain*) or (weight adj2 loss) or "lose weight" or "losing weight" or (weight adj1 (reduc* or manage* or control*))).ti,ab,kf,ot. (367106) 13 ("weight adj1 measurement*" or "height adj1 measurement*").ti,ab,kf,ot. (0) 14 ("healthy weight" or "unhealthy weight" or "weight adj5 monitor*" or "height adj5 monitor*").ti,ab,kf,ot. (6736) 15 ("being measured" and ("weight" or weighing or height)).ti,ab,kf,ot. (229) 16 (weighing adj3 experiences).ti,ab,kf,ot. (8) 17 (weight screening* or height screening*).ti,ab,kf,ot. (89) 18 ("being fat" or "being weighed" or "Being thin" or "Being tall" or "Being short").ti,ab,kf,ot. (1003) 19 ((stereotype or stereotypes or Stigma or stigmati* or ideals or ideal or discriminate* or discrimination or "peer pressure" or prejudice* or bully* or teasing or cyberbull* or bullied) adj5 ("fat" or weight or thin or height or tall or short or muscle* or muscular* or body)).ti,ab,kf,ot. (16698) 20 ((fat or weight or thin or body or height or muscle or muscular*) adj5 (acceptability or satisfaction or satisfied or dissatisfaction or dissatisfied or anxiety or anxious or angst or peer pressure or feelings or worries or worry or concern or concerns or "over concern" or emotions or emotion or concerns or "self-worth" or "self esteem" or esteem or "self-concept" or "self-identification" or "pre-occupation" or "preoccupation" or "acceptability" or perceiv* or understand* or perception* or internalis* or attitud*)).ti,ab,kf,ot. (52806) 21 (Body adj1 (dysmorph* or dismorph*)).ti,ab,kf,ot. (2295) 22 (Muscle adj1 (dysmorph* or dismorph*)).ti,ab,kf,ot. (326) 23 ((body or fat or thin or height or weight) adj3 (shame or phobia*)).ti,ab,kf,ot. (725) 24 ((weight adj2 muscle) or "excess weight").ti,ab,kf,ot. (17151) 25 ((weight adj3 appearance) or (height adj3 appearance)).ti,ab,kf,ot. (1387) 26 5 or 6 or 7 or 8 or 9 or 10 or 11 or 12 or 13 or 14 or 15 or 16 or 17 or 18 or 19 or 20 or 21 or 22 or 23 or 24 or 25 (1344408) 27 Body Dysmorphic Disorders/ or Weight Prejudice/ or "body size"/ or "body weight"/ or exp "ideal body weight"/ or "obesity"/ or "overweight"/ or exp "thinness"/ (1022899) 28 body height/ (98524) 29 27 or 28 (1061654) 30 26 or 29 (1689993) 31 4 and 30 (322053) 32 "attitude to health"/ or "comprehension"/ or "ethnopsychology"/ or "Focus Groups"/ or "Interviews as Topic"/ or Qualitative Research/ or "attitude"/ or Personal Narrative/ (933146) 33 ((Girls or Boys or Child* or teen* or adolescen* or "young people*" or schoolchild* or pupil* or student*) adj2 ("perspective*" or "experience*" or "lived experience*" or "attitude" or "attitudes" or "attitudinal" or "belief" or "beliefs" or "discourse" or "discourses" or "life-world" or "opinions" or "standpoint" or "standpoints" or "understanding" or "understandings" or "viewpoint" or "viewpoints" or "views" or "voice" or "voices" or discussed or described* or drawings or drew)).ab. (92336) 34 ("audiorecording" or "ethnograph*" or "ethnolog*" or "ethnopsycholog*" or "ethno psycholog*" or "Focus Groups" or "Focus Group" or "repertory grid*" or "stories" or "audio record" or "audio recorded" or "audio recorder" or "audio recording" or "audio recordings" or "audio records" or "thematic analysis" or "phenomenol*" or "grounded theory" or "grounded studies" or "grounded research" or "purposive sampling" or "biographical method" or "theoretical sampl*" or "conversation analysis" or "theoretical saturation" or "thematic analyses" or "mixed design" or "mixed method" or "mixed methods" or "qualitative" or "interviewed" or "interviewing" or "interviewer" or "interviews" or "interview" or "narratives" or "Diary entries" or "diary study" or Diaries or journalling or (Questions* adj5 (semi-structured, or in-depth or open or "open ended")) or "Group discussion*").ti,ab,kf,ot. (1087193) 35 ("experiences" or "lived experience*" or "perceptions" or "perspective" or "perspectives" or "experience" or "attitude" or "attitudes" or "attitudinal" or "belief" or "beliefs" or "discourse" or "discourses" or "life-world" or "opinions" or "perceived" or "perception" or "standpoint" or "standpoints" or "understanding" or "understandings" or "viewpoint" or "viewpoints" or "views" or "voice" or "voices" or talk*).ti,ot,kf. (1217235) 36 32 or 33 or 34 or 35 (2627628) 37 4 and 30 and 36 (31033) 38 exp United Kingdom/ (518151) 39 (England not "New England").ti,ab,kf,ot,in,jw. (215723) 40 ("district council" or "local council" or "local authorities" or "NHS Trust" or "primary care trust" or "borough council" or "county council" or "local authority" or "district councils" or "local councils" or "NHS Trusts" or "primary care trusts" or "borough councils" or "county councils" or "Social Care Trust").ti,ab,ot,kf,in. (198538) 41 ("United Kingdom" or UK or "U.K." or Britain or GB or (British not "British Columbia")).ti,ab,ot,kf,in,jw. (3853571) 42 (Welsh or Scottish or "northern Irish" or Wales or Scotland or "northern Ireland").ti,ab,ot,kf,in,jw. (336862) 43 ("English population?" or "English longitudinal" or "English town?" or "English count*" or "English city" or "English cities" or "English health").ti,ab,kf,ot,in. (2881) 44 (national health service* or nhs*).ti,ab,in. (451817) 45 (english not ((published or publication* or translat* or written or language* or speak* or literature or citation*) adj5 english)).ti,ab. (70324) 46 (bath or "bath's" or ((birmingham not alabama*) or ("birmingham's" not alabama*) or bradford or "bradford's" or brighton or "brighton's" or bristol or "bristol's" or carlisle* or "carlisle's" or (cambridge not (massachusetts* or boston* or harvard*)) or ("cambridge's" not (massachusetts* or boston* or harvard*)) or (canterbury not zealand*) or ("canterbury's" not zealand*) or chelmsford or "chelmsford's" or chester or "chester's" or chichester or "chichester's" or coventry or "coventry's" or derby or "derby's" or (durham not (carolina* or nc)) or ("durham's" not (carolina* or nc)) or ely or "ely's" or exeter or "exeter's" or gloucester or "gloucester's" or hereford or "hereford's" or hull or "hull's" or lancaster or "lancaster's" or leeds* or leicester or "leicester's" or (lincoln not nebraska*) or ("lincoln's" not nebraska*) or (liverpool not (new south wales* or nsw)) or ("liverpool's" not (new south wales* or nsw)) or ((london not (ontario* or ont or toronto*)) or ("london's" not (ontario* or ont or toronto*)) or manchester or "manchester's" or (newcastle not (new south wales* or nsw)) or ("newcastle's" not (new south wales* or nsw)) or norwich or "norwich's" or nottingham or "nottingham's" or oxford or "oxford's" or peterborough or "peterborough's" or plymouth or "plymouth's" or portsmouth or "portsmouth's" or preston or "preston's" or ripon or "ripon's" or salford or "salford's" or salisbury or "salisbury's" or sheffield or "sheffield's" or southampton or "southampton's" or st albans or stoke or "stoke's" or sunderland or "sunderland's" or truro or "truro's" or wakefield or "wakefield's" or wells or westminster or "westminster's" or winchester or "winchester's" or wolverhampton or "wolverhampton's" or (worcester not (massachusetts* or boston* or harvard*)) or ("worcester's" not (massachusetts* or boston* or harvard*)) or (york not ("new york*" or ny or ontario* or ont or toronto*)) or ("york's" not ("new york*" or ny or ontario* or ont or toronto*))))).ti,ab,in. (3183056) 47 (aberdeen or "aberdeen's" or dundee or "dundee's" or edinburgh or "edinburgh's" or glasgow or "glasgow's" or inverness or (perth not australia*) or ("perth's" not australia*) or stirling or "stirling's").ti,ab,in. (439828) 48 (armagh or "armagh's" or belfast or "belfast's" or lisburn or "lisburn's" or londonderry or "londonderry's" or derry or "derry's" or newry or "newry's").ti,ab,in. (61971) 49 ("South Holland" or Aldershot or Ashfield or Barking or Barnet or Barnsley or Bedfordshire or Bexley or Birkenhead or Blackburn or Blackpool or Bolton or Bournemouth or Brent or Bridgend or Bromley or Bromwich or Buckinghamshire or Burnley or Camberwell or Cambridgeshire or Camden or Chelsea or Chelsea or Cheshire or Cleveland or Colchester or Cornwall or Crawley or Croydon or Cumbria or Dagenham or Dartford or Derbyshire or Devon or Doncaster or Dorset or Dudley or Ealing or Ealing or Eastbourne or Enfield or Essex or Farnborough or Fulham or Furness or Galloway or Gateshead or Glamorgan or Glasgow or Gloucestershire or Gravesham or Greenwich or Grimsby or Guildford or Hackney or Hamlets or Hammersmith or Hampshire or Haringey or Haringey or Harrow or Hartlepool or Harwell or Hastings or Havering or Helens or Hertfordshire or Highland or Hillingdon or Hounslow or Hounslow or Hove or Huddersfield or Humber or Ipswich or Islington or Kensington or Kent or Kingston or Kirklees or Knowsley or Lambeth or Lancashire or Leicestershire or Lewisham or Lichfield or Lincoln or Lincolnshire or Loughborough or Luton or Lynn or Mansfield or Merseyside or Merton or Middlesbrough or Midlands or Milton Keynes or Newcastle or Newham or Norfolk or Northampton or Northamptonshire or Northumberland or Nottinghamshire or Oadby or Oldham or Oxfordshire or Poole or Portsmouth or Reading or Redbridge or Redcar or Richmond or Rochdale or Rotherham or Rushmoor or Sandwell or Scarborough or Scilly or Shropshire or Slough or Solihull or Somerset or Southampton or Southend or Southwark or Staffordshire or Stockport or Stockton or Suffolk or Surrey or Sussex or Sutton or Swindon or Teesside or Telford or Thurrock or Tower Hamlets or Tyne or Tyneside or Walsall or Waltham or Wandsworth or Warrington or Warwickshire or Watford or Wigan or Wight or Wigston or Wiltshire or Wirral or Woking or Worcestershire or Worthing or Yorkshire).ti,ab,kf,ot,in. (2002713) 50 ("Isle of Man" or "Channel Islands" or "Guernsey").ti,ab,kf,ot,in. (2071) 51 (bangor or "bangor's" or cardiff or "cardiff's" or newport or "newport's" or "st asaph" or "st asaph's" or "st davids" or swansea or "swansea's").ti,ab,in. (129425) 52 38 or 39 or 40 or 41 or 42 or 43 or 44 or 45 or 46 or 47 or 48 or 49 or 50 or 51 (5945097) 53 (exp africa/ or exp americas/ or exp antarctic regions/ or exp arctic regions/ or exp asia/ or exp australia/ or exp oceania/ or developing countries/) not (exp United Kingdom/ or europe/) (4107942) 54 52 not 53 (5531023) 55 37 and 54 (4470) 56 limit 55 to yr="2008 -Current" (3766) 57 obesity/ or adolescent obesity/ or childhood obesity/ or normal weight obesity/ (627175) 58 body weight gain/ (139835) 59 body weight loss/ or body weight change/ (277952) 60 body weight change/ (23718) 61 body weight/ (444051) 62 body weight control/ (8581) 63 weight bias/ or weight discrimination/ (623) 64 body height/ (98524) 65 body size/ (34263) 66 ideal body weight/ (1843) 67 body image/ or body dissatisfaction/ (30750) 68 body dysmorphic disorder/ or muscle dysmorphia/ (4514) 69 underweight/ (26772) 70 57 or 58 or 59 or 60 or 61 or 62 or 63 or 64 or 65 or 66 or 67 or 68 or 69 (1373668) 71 (Weight not "birth weight").ti. (142755) 72 ("anti-fat" or bodyweight or "obese" or "obesity" or "overweight" or "skinny" or "fatness" or "thinness" or "body image" or "body hatred" or "body positive" or "thin ideal*" or "fat ideal*").ti,ab,kf. (709334) 73 ("body esteem" and ("weight" or height or fat or thin)).ti,ab,kf. (340) 74 ((body adj2 ideal*) or (body adj2 shape) or (body adj2 shapes) or (body adj1 size)).ti,ab,kf. (42125) 75 ((body adj10 weight) not (body adj10 "birth weight")).ti,ab,kf. (419931) 76 ("underweight" not "birth weight").ti,ab,kf. (24473) 77 ((weight adj1 bias) or (weight adj1 biases)).ti,ab,kf. (974) 78 ("weight change" or "weight changes" or (weight adj2 gain*) or (weight adj2 loss) or "lose weight" or "losing weight" or (weight adj1 (reduc* or manage* or control*))).ti,ab,kf. (367102) 79 ("weight adj1 measurement*" or "height adj1 measurement*").ti,ab,kf. (0) 80 ("healthy weight" or "unhealthy weight" or "weight adj5 monitor*" or "height adj5 monitor*").ti,ab,kf. (6736) 81 ("being measured" and ("weight" or weighing or height)).ti,ab,kf. (229) 82 (weighing adj3 experiences).ti,ab,kf. (8) 83 (weight screening* or height screening*).ti,ab,kf. (89) 84 ("being fat" or "being weighed" or "Being thin" or "Being tall" or "Being short").ti,ab,kf. (1003) 85 ((stereotype or stereotypes or Stigma or stigmati* or ideals or ideal or discriminate* or discrimination* or "peer pressure" or prejudice* or bully* or teasing or cyberbull* or bullied) adj5 ("fat" or weight or thin or height or muscle* or muscular* or body)).ti,ab,kf. (14956) 86 ((fat or weight or thin or body or height or muscle or muscular*) adj5 (acceptability or satisfaction or satisfied or dissatisfaction or dissatisfied or anxiety or anxious or angst or peer pressure or feelings or worries or worry or concern or concerns or "over concern" or emotions or emotion or concerns or "self-worth" or "self esteem" or esteem or "self-concept" or "self-identification" or "pre-occupation" or "preoccupation" or "acceptability" or perceiv* or understand* or perception* or internalis* or attitud*)).ti,ab,kf. (52805) 87 (Body adj1 (dysmorph* or dismorph*)).ti,ab,kf. (2295) 88 (Muscle adj1 (dysmorph* or dismorph*)).ti,ab,kf. (326) 89 ((body or fat or thin or height or weight) adj3 (shame or phobia*)).ti,ab,kf. (725) 90 ((weight adj2 muscle) or "excess weight").ti,ab,kf. (17151) 91 ((weight adj3 appearance) or (height adj3 appearance)).ti,ab,kf. (1387) 92 71 or 72 or 73 or 74 or 75 or 76 or 77 or 78 or 79 or 80 or 81 or 82 or 83 or 84 or 85 or 86 or 87 or 88 or 89 or 90 or 91 (1342635) 93 70 or 92 (1802348) 94 child/ or adolescent/ or juvenile/ or school child/ or boy/ or girl/ (3787214) 95 ("young people*" or adolescent* or youth or pubertal or pubescent or "pre adolescent" or "Pre pubescent" or "pre pubertal" or teen* or preteen* or tweens or tweenage* or youth or youths or schoolboy* or schoolgirl* or "school aged" or "young person*" or juvenile* or "Boy" or "boys" or "child" or "children*" or "child's" or "Girl" or "girls" or "Minors" or "preadolescent" or "Prepubescent" or "schoolchild*" or pubescent or "early adolescent*").ti,ab,kf. (2901273) 96 ((child* or adolescen*) not (childbirth or "child birth")).jx. (293437) 97 94 or 95 or 96 (4679869) 98 qualitative research/ or phenomenology/ or ethnography/ or ethnology/ or personal experience/ or ethnographic research/ (304854) 99 semi structured interview/ or unstructured interview/ or (interview/ not structured interview/) (341103) 100 attitude/ (82568) 101 attitude to health/ (139503) 102 comprehension/ (37759) 103 perception/ (178907) 104 thematic analysis/ (44411) 105 ("experiences" or "lived experience*" or "perceptions" or "perspective" or "perspectives" or "experience" or "attitude" or "attitudes" or "attitudinal" or "belief" or "beliefs" or "discourse" or "discourses" or "life-world" or "opinions" or "perceived" or "perception" or "standpoint" or "standpoints" or "understanding" or "understandings" or "viewpoint" or "viewpoints" or "views" or "voice" or "voices" or talk*).ti,kf. (1210769) 106 ((Girls or Boys or Child* or teen* or adolescen* or "young people*" or schoolchild* or pupil* or student*) adj2 ("perspective*" or "experience*" or "lived experience*" or "attitude" or "attitudes" or "attitudinal" or "belief" or "beliefs" or "discourse" or "discourses" or "life-world" or "opinions" or "standpoint" or "standpoints" or "understanding" or "understandings" or "viewpoint" or "viewpoints" or "views" or "voice" or "voices" or discussed or described* or drawings or drew)).ab. (92336) 107 ("audiorecording" or "ethnograph*" or "ethnolog*" or "ethno psycholog*" or "ethnopsycholog*" or "Focus Groups" or "Focus Group" or "repertory grid" or "stories" or "audio record" or "audio recorded" or "audio recorder" or "audio recording" or "audio recordings" or "audio records" or "thematic analysis" or "phenomenol*" or "grounded theory" or "grounded studies" or "grounded research" or "purposive sampling" or "biographical method" or "theoretical sampl*" or "conversation analysis" or "theoretical saturation" or "mixed design" or "mixed method" or "mixed methods" or "qualitative" or "interviewed" or "interviewing" or "interviewer" or "narratives" or "Diary entries" or "diary study" or Diaries or journalling or (Question* adj5 (semi-structured or semistructured or unstructured or informal or in-depth or indepth or open or "open ended")) or "Group discussion*" or ((("interviews" or "interview") not "structured interview*") or "semi structured interview*")).ti,ab,kf. (1090774) 108 98 or 99 or 100 or 101 or 102 or 103 or 104 or 105 or 106 or 107 (2530447) 109 united kingdom/ or exp great britain/ or exp northern ireland/ (518151) 110 ("district council" or "local council" or "local authorities" or "NHS Trust" or "primary care trust" or "borough council" or "county council" or "local authority" or "district councils" or "local councils" or "NHS Trusts" or "primary care trusts" or "borough councils" or "county councils" or "Social Care Trust").ti,ab,ot,kf,in,ad. (200476) 111 (national health service* or nhs*).ti,ab,in,ad. (520773) 112 (gb or "g.b." or britain* or (british* not "british columbia") or uk or "u.k." or "united kingdom*" or (england* not "new england") or "northern ireland*" or irish* or scotland* or scottish* or ((wales or "south wales") not "new south wales") or welsh*).ti,ab,ot,kf,in,jx,ad. (4156904) 113 (english not ((published or publication* or translat* or written or language* or speak* or literature or citation*) adj5 english)).ti,ab. (70324) 114 (bath or "bath's" or ((birmingham not alabama*) or ("birmingham's" not alabama*) or bradford or "bradford's" or brighton or "brighton's" or bristol or "bristol's" or carlisle* or "carlisle's" or (cambridge not (massachusetts* or boston* or harvard*)) or ("cambridge's" not (massachusetts* or boston* or harvard*)) or (canterbury not zealand*) or ("canterbury's" not zealand*) or chelmsford or "chelmsford's" or chester or "chester's" or chichester or "chichester's" or coventry or "coventry's" or derby or "derby's" or (durham not (carolina* or nc)) or ("durham's" not (carolina* or nc)) or ely or "ely's" or exeter or "exeter's" or gloucester or "gloucester's" or hereford or "hereford's" or hull or "hull's" or lancaster or "lancaster's" or leeds* or leicester or "leicester's" or (lincoln not nebraska*) or ("lincoln's" not nebraska*) or (liverpool not (new south wales* or nsw)) or ("liverpool's" not (new south wales* or nsw)) or ((london not (ontario* or ont or toronto*)) or ("london's" not (ontario* or ont or toronto*)) or manchester or "manchester's" or (newcastle not (new south wales* or nsw)) or ("newcastle's" not (new south wales* or nsw)) or norwich or "norwich's" or nottingham or "nottingham's" or oxford or "oxford's" or peterborough or "peterborough's" or plymouth or "plymouth's" or portsmouth or "portsmouth's" or preston or "preston's" or ripon or "ripon's" or salford or "salford's" or salisbury or "salisbury's" or sheffield or "sheffield's" or southampton or "southampton's" or st albans or stoke or "stoke's" or sunderland or "sunderland's" or truro or "truro's" or wakefield or "wakefield's" or wells or westminster or "westminster's" or winchester or "winchester's" or wolverhampton or "wolverhampton's" or (worcester not (massachusetts* or boston* or harvard*)) or ("worcester's" not (massachusetts* or boston* or harvard*)) or (york not ("new york*" or ny or ontario* or ont or toronto*)) or ("york's" not ("new york*" or ny or ontario* or ont or toronto*))))).ti,ab,in,ad. (3211191) 115 (aberdeen or "aberdeen's" or dundee or "dundee's" or edinburgh or "edinburgh's" or glasgow or "glasgow's" or inverness or (perth not australia*) or ("perth's" not australia*) or stirling or "stirling's").ti,ab,in,ad. (442866) 116 (armagh or "armagh's" or belfast or "belfast's" or lisburn or "lisburn's" or londonderry or "londonderry's" or derry or "derry's" or newry or "newry's").ti,ab,in,ad. (62265) 117 ("South Holland" or Aldershot or Ashfield or Barking or Barnet or Barnsley or Bedfordshire or Bexley or Birkenhead or Blackburn or Blackpool or Bolton or Bournemouth or Brent or Bridgend or Bromley or Bromwich or Buckinghamshire or Burnley or Camberwell or Cambridgeshire or Camden or Chelsea or Chelsea or Cheshire or Cleveland or Colchester or Cornwall or Crawley or Croydon or Cumbria or Dagenham or Dartford or Derbyshire or Devon or Doncaster or Dorset or Dudley or Ealing or Ealing or Eastbourne or Enfield or Essex or Farnborough or Fulham or Furness or Galloway or Gateshead or Glamorgan or Glasgow or Gloucestershire or Gravesham or Greenwich or Grimsby or Guildford or Hackney or Hamlets or Hammersmith or Hampshire or Haringey or Haringey or Harrow or Hartlepool or Harwell or Hastings or Havering or Helens or Hertfordshire or Highland or Hillingdon or Hounslow or Hounslow or Hove or Huddersfield or Humber or Ipswich or Islington or Kensington or Kent or Kingston or Kirklees or Knowsley or Lambeth or Lancashire or Leicestershire or Lewisham or Lichfield or Lincoln or Lincolnshire or Loughborough or Luton or Lynn or Mansfield or Merseyside or Merton or Middlesbrough or Midlands or Milton Keynes or Newcastle or Newham or Norfolk or Northampton or Northamptonshire or Northumberland or Nottinghamshire or Oadby or Oldham or Oxfordshire or Poole or Portsmouth or Reading or Redbridge or Redcar or Richmond or Rochdale or Rotherham or Rushmoor or Sandwell or Scarborough or Scilly or Shropshire or Slough or Solihull or Somerset or Southampton or Southend or Southwark or Staffordshire or Stockport or Stockton or Suffolk or Surrey or Sussex or Sutton or Swindon or Teesside or Telford or Thurrock or Tower Hamlets or Tyne or Tyneside or Walsall or Waltham or Wandsworth or Warrington or Warwickshire or Watford or Wigan or Wight or Wigston or Wiltshire or Wirral or Woking or Worcestershire or Worthing or Yorkshire).ti,ab,kf,ot,in,ad. (2050169) 118 ("Isle of Man" or "Channel Islands" or "Guernsey").ti,ab,kf,ot,in,ad. (2119) 119 (bangor or "bangor's" or cardiff or "cardiff's" or newport or "newport's" or "st asaph" or "st asaph's" or "st davids" or swansea or "swansea's").ti,ab,in,ad. (130215) 120 109 or 110 or 111 or 112 or 113 or 114 or 115 or 116 or 117 or 118 or 119 (6045726) 121 93 and 97 and 108 and 120 (5942) 122 (exp "arctic and antarctic"/ or exp oceanic regions/ or exp western hemisphere/ or exp eastern hemisphere/ or exp africa/ or exp asia/ or exp "Australia and New Zealand"/ or southern Europe/) not (exp united kingdom/ or europe/) (5406841) 123 121 not 122 (4435) 124 limit 123 to yr="1934 - 2007" (757) 125 123 not 124 (3678) 126 limit 125 to english language (3669) 127 limit 126 to "remove preprint records" (3657) 128 limit 127 to conference abstract status (1019) 129 105 or 106 or 107 (2168397) 130 92 and 95 and 129 (18083) 131 128 and 130 (804) 132 127 not 128 (2638) 133 131 or 132 (3442) 134 adult/ not (Adult/ and (child/ or adolescent/ or juvenile/ or school child/ or boy/ or girl/)) (8921908) 135 133 and 134 (209) 136 133 not 135 (3233) 137 limit 136 to (editorial or letter or "review") (348) 138 136 not 137 (2885) |
| Items coded | 1652 |
| Uploaded documents | 9 |
| Masters of duplicates | 275 |
| Deleted items | 1233 |
| Outcomes | 0 |
| Import filter | RIS |
| Is deleted? | false |

| Source name | **Psycinfo** |
| --- | --- |
| Database name/platform | **PsycINFO (OVID)** |
| Date of search | Jan 31, 2025 |
| Date of import | Jan 31, 2025 |
| Number items | 1470 |
| Duplicates | 554 |
| Description |  |
| Notes |  |
| Search string | Database: APA PsycInfo <1806 to January 2025 Week 4> Search Strategy: 1 ("young people*" or adolescent* or youth or pubertal or pubescent or "pre adolescent" or "Pre pubescent" or "pre pubertal" or teen* or preteen* or tweens or tweenage* or youth or youths or schoolboy* or schoolgirl* or "school aged" or "young person*" or juvenile* or "Boy" or "boys" or "child" or "children*" or "child's" or "Girl" or "girls" or "Minors" or "preadolescent" or "Prepubescent" or "schoolchild*" or pubescent or "early adolescent*").ti,ab,id. (1062550) 2 (Weight not "birth weight").ti. (17371) 3 ("anti-fat" or bodyweight or "obese" or "obesity" or "overweight" or "skinny" or "fatness" or "thinness" or "body image" or "body hatred" or "body positive" or "thin ideal*" or "fat ideal*").ti,ab,id. (71690) 4 ("body esteem" and ("weight" or height or fat or thin)).ti,ab,id. (361) 5 ((body adj2 ideal*) or (body adj2 shape) or (body adj2 shapes) or (body adj1 size)).ti,ab,id. (8569) 6 ((body adj10 weight) not (body adj10 "birth weight")).ti,ab,id. (25402) 7 ("underweight" not "birth weight").ti,ab,id. (2550) 8 ((weight adj1 bias) or (weight adj1 biases)).ti,ab,id. (617) 9 ("weight change" or "weight changes" or (weight adj2 gain*) or (weight adj2 loss) or "lose weight" or "losing weight" or (weight adj1 (reduc* or manage* or control*))).ti,ab,id. (32819) 10 ("weight adj1 measurement*" or "height adj1 measurement*").ti,ab,id. (0) 11 ("healthy weight" or "unhealthy weight" or "weight adj5 monitor*" or "height adj5 monitor*").ti,ab,id. (1969) 12 ("being measured" and ("weight" or weighing or height)).ti,ab,id. (28) 13 (weighing adj3 experiences).ti,ab,id. (2) 14 (weight screening* or height screening*).ti,ab,id. (9) 15 ("being fat" or "being weighed" or "Being thin" or "Being tall" or "Being short").ti,ab,id. (296) 16 ((stereotype or stereotypes or Stigma or stigmati* or ideals or ideal or discriminate* or discrimination* or "peer pressure" or prejudice* or bully* or teasing or cyberbull* or bullied) adj5 ("fat" or weight or thin or height or muscle* or muscular* or body)).ti,ab,id. (6281) 17 ((fat or weight or thin or body or height or muscle or muscular*) adj5 (acceptability or satisfaction or satisfied or dissatisfaction or dissatisfied or anxiety or anxious or angst or peer pressure or feelings or worries or worry or concern or concerns or "over concern" or emotions or emotion or concerns or "self-worth" or "self esteem" or esteem or "self-concept" or "self-identification" or "pre-occupation" or "preoccupation" or "acceptability" or perceiv* or understand* or perception* or internalis* or attitud*)).ti,ab,id. (30591) 18 (Body adj1 (dysmorph* or dismorph*)).ti,ab,id. (1760) 19 (Muscle adj1 (dysmorph* or dismorph*)).ti,ab,id. (356) 20 ((body or fat or thin or height or weight) adj3 (shame or phobia*)).ti,ab,id. (947) 21 ((weight adj2 muscle) or "excess weight").ti,ab,id. (1009) 22 ((weight adj3 appearance) or (height adj3 appearance)).ti,ab,id. (503) 23 ((Girls or Boys or Child* or teen* or adolescen* or "young people*" or schoolchild* or pupil* or student*) adj2 ("perspective*" or "experience*" or "lived experience*" or "attitude" or "attitudes" or "attitudinal" or "belief" or "beliefs" or "discourse" or "discourses" or "life-world" or "opinions" or "standpoint" or "standpoints" or "understanding" or "understandings" or "viewpoint" or "viewpoints" or "views" or "voice" or "voices" or discussed or described* or drawings or drew)).ab. (109496) 24 ("audiorecording" or "ethnograph*" or "ethnolog*" or "ethno psycholog*" or "ethnopsycholog*" or "Focus Groups" or "Focus Group" or "repertory grid" or "stories" or "audio record" or "audio recorded" or "audio recorder" or "audio recording" or "audio recordings" or "audio records" or "thematic analysis" or "phenomenol*" or "grounded theory" or "grounded studies" or "grounded research" or "purposive sampling" or "biographical method" or "theoretical sampl*" or "conversation analysis" or "theoretical saturation" or "thematic analyses" or "mixed design" or "mixed method" or "mixed methods" or "qualitative" or "interviewed" or "interviewing" or "interviewer" or "interviews" or "interview" or "narratives" or "Diary entries" or "diary study" or Diaries or journalling or (Questions* adj5 (semi-structured, or in-depth or open or "open ended")) or "Group discussion*").ti,ab,id. (702465) 25 ("experiences" or "lived experience*" or "perceptions" or "perspective" or "perspectives" or "experience" or "attitude" or "attitudes" or "attitudinal" or "belief" or "beliefs" or "discourse" or "discourses" or "life-world" or "opinions" or "perceived" or "perception" or "standpoint" or "standpoints" or "understanding" or "understandings" or "viewpoint" or "viewpoints" or "views" or "voice" or "voices" or talk*).ti,id. (800907) 26 ((child* or adolescen*) not (childbirth or "child birth")).jw. (177253) 27 exp Child Attitudes/ (8238) 28 adolescent attitudes/ (22220) 29 attitudes/ (32976) 30 "Body Dysmorphic Disorder"/ or "Body Image Disturbances"/ or Body Image/ (16802) 31 body image/ or body dissatisfaction/ or body esteem/ or muscle dysmorphia/ (14884) 32 body size/ or body height/ or exp body weight/ (56704) 33 body mass index/ (9214) 34 body weight/ or exp overweight/ or underweight/ or weight control/ or weight gain/ or weight loss/ or body fat/ or "obesity (attitudes toward)"/ or weight-based discrimination/ (51107) 35 obesity/ (30542) 36 30 or 31 or 32 or 33 or 34 or 35 (76329) 37 2 or 3 or 4 or 5 or 6 or 7 or 8 or 9 or 10 or 11 or 12 or 13 or 14 or 15 or 16 or 17 or 18 or 19 or 20 or 21 or 22 or 30 or 31 or 32 or 33 or 34 or 35 (133821) 38 1 or 26 or 27 or 28 (1090686) 39 focus group/ or qualitative methods/ or focus group interview/ (12769) 40 group discussion/ or "obesity (attitudes toward)"/ (4607) 41 interpretative phenomenological analysis/ or narrative analysis/ or semi-structured interview/ or thematic analysis/ or phenomenology/ (24820) 42 interviews/ (13365) 43 (interview or "focus group" or "qualitative study").md. (537864) 44 23 or 24 or 25 or 27 or 28 or 29 or 39 or 40 or 41 or 42 or 43 (1536249) 45 37 and 44 (34634) 46 limit 45 to ("180 school age (age 6 to 12 yrs)" or "200 adolescence (age 13 to 17 yrs)") (8161) 47 1 or 26 or 27 or 28 (1090686) 48 45 and 47 (10917) 49 46 or 48 (12294) 50 limit 49 to yr="1860 - 2007" (3120) 51 49 not 50 (9174) 52 limit 51 to (afrikaans or albanian or arabic or bulgarian or catalan or chinese or czech or danish or dutch or finnish or french or georgian or german or greek or hebrew or hindi or hungarian or iranian or italian or japanese or korean or lithuanian or malaysian or nonenglish or norwegian or polish or portuguese or romanian or russian or serbo croatian or slovak or slovene or spanish or swedish or turkish or ukrainian) (243) 53 51 not 52 (8931) 54 (England not "New England").ti,ab,id,ot,in,jw. (127175) 55 ("district council" or "local council" or "local authorities" or "NHS Trust" or "primary care trust" or "borough council" or "county council" or "local authority" or "district councils" or "local councils" or "NHS Trusts" or "primary care trusts" or "borough councils" or "county councils" or Eur or "Social Care Trust").ti,ab,ot,id,in. (16571) 56 (Welsh or Scottish or "Irish" or Wales or Scotland or "northern Ireland").ti,ab,ot,id,in,jw. (79378) 57 (national health service* or nhs*).ti,ab,in. (32180) 58 (english not ((published or publication* or translat* or written or language* or speak* or literature or citation*) adj5 english)).ti,ab. (104718) 59 (bath or "bath's" or ((birmingham not alabama*) or ("birmingham's" not alabama*) or bradford or "bradford's" or brighton or "brighton's" or bristol or "bristol's" or carlisle* or "carlisle's" or (cambridge not (massachusetts* or boston* or harvard*)) or ("cambridge's" not (massachusetts* or boston* or harvard*)) or (canterbury not zealand*) or ("canterbury's" not zealand*) or chelmsford or "chelmsford's" or chester or "chester's" or chichester or "chichester's" or coventry or "coventry's" or derby or "derby's" or (durham not (carolina* or nc)) or ("durham's" not (carolina* or nc)) or ely or "ely's" or exeter or "exeter's" or gloucester or "gloucester's" or hereford or "hereford's" or hull or "hull's" or lancaster or "lancaster's" or leeds* or leicester or "leicester's" or (lincoln not nebraska*) or ("lincoln's" not nebraska*) or (liverpool not (new south wales* or nsw)) or ("liverpool's" not (new south wales* or nsw)) or ((london not (ontario* or ont or toronto*)) or ("london's" not (ontario* or ont or toronto*)) or manchester or "manchester's" or (newcastle not (new south wales* or nsw)) or ("newcastle's" not (new south wales* or nsw)) or norwich or "norwich's" or nottingham or "nottingham's" or oxford or "oxford's" or peterborough or "peterborough's" or plymouth or "plymouth's" or portsmouth or "portsmouth's" or preston or "preston's" or ripon or "ripon's" or salford or "salford's" or salisbury or "salisbury's" or sheffield or "sheffield's" or southampton or "southampton's" or st albans or stoke or "stoke's" or sunderland or "sunderland's" or truro or "truro's" or wakefield or "wakefield's" or wells or westminster or "westminster's" or winchester or "winchester's" or wolverhampton or "wolverhampton's" or (worcester not (massachusetts* or boston* or harvard*)) or ("worcester's" not (massachusetts* or boston* or harvard*)) or (york not ("new york*" or ny or ontario* or ont or toronto*)) or ("york's" not ("new york*" or ny or ontario* or ont or toronto*))))).ti,ab,in. (434800) 60 (aberdeen or "aberdeen's" or dundee or "dundee's" or edinburgh or "edinburgh's" or glasgow or "glasgow's" or inverness or (perth not australia*) or ("perth's" not australia*) or stirling or "stirling's").ti,ab,in. (53869) 61 (armagh or "armagh's" or belfast or "belfast's" or lisburn or "lisburn's" or londonderry or "londonderry's" or derry or "derry's" or newry or "newry's").ti,ab,in. (7442) 62 ("South Holland" or Aldershot or Ashfield or Barking or Barnet or Barnsley or Bedfordshire or Bexley or Birkenhead or Blackburn or Blackpool or Bolton or Bournemouth or Brent or Bridgend or Bromley or Bromwich or Buckinghamshire or Burnley or Camberwell or Cambridgeshire or Camden or Chelsea or Chelsea or Cheshire or Cleveland or Colchester or Cornwall or Crawley or Croydon or Cumbria or Dagenham or Dartford or Derbyshire or Devon or Doncaster or Dorset or Dudley or Ealing or Ealing or Eastbourne or Enfield or Essex or Farnborough or Fulham or Furness or Galloway or Gateshead or Glamorgan or Glasgow or Gloucestershire or Gravesham or Greenwich or Grimsby or Guildford or Hackney or Hamlets or Hammersmith or Hampshire or Haringey or Haringey or Harrow or Hartlepool or Harwell or Hastings or Havering or Helens or Hertfordshire or Highland or Hillingdon or Hounslow or Hounslow or Hove or Huddersfield or Humber or Ipswich or Islington or Kensington or Kent or Kingston or Kirklees or Knowsley or Lambeth or Lancashire or Leicestershire or Lewisham or Lichfield or Lincoln or Lincolnshire or Loughborough or Luton or Lynn or Mansfield or Merseyside or Merton or Middlesbrough or Midlands or Milton Keynes or Newcastle or Newham or Norfolk or Northampton or Northamptonshire or Northumberland or Nottinghamshire or Oadby or Oldham or Oxfordshire or Poole or Portsmouth or Reading or Redbridge or Redcar or Richmond or Rochdale or Rotherham or Rushmoor or Sandwell or Scarborough or Scilly or Shropshire or Slough or Solihull or Somerset or Southampton or Southend or Southwark or Staffordshire or Stockport or Stockton or Suffolk or Surrey or Sussex or Sutton or Swindon or Teesside or Telford or Thurrock or Tower Hamlets or Tyne or Tyneside or Walsall or Waltham or Wandsworth or Warrington or Warwickshire or Watford or Wigan or Wight or Wigston or Wiltshire or Wirral or Woking or Worcestershire or Worthing or Yorkshire).ti,ab,ot,in. (369421) 63 ("Isle of Man" or "Channel Islands" or "Guernsey").ti,ab,ot,in. (407) 64 (bangor or "bangor's" or cardiff or "cardiff's" or newport or "newport's" or "st asaph" or "st asaph's" or "st davids" or swansea or "swansea's").ti,ab,in. (22804) 65 ("United Kingdom" or UK or "U.K." or Britain or GB or (British not "British Columbia")).ti,ab,ot,id,in,jw. (444946) 66 54 or 55 or 56 or 57 or 58 or 59 or 60 or 61 or 62 or 63 or 64 or 65 (961721) 67 53 and 66 (1470) |
| Items coded | 916 |
| Uploaded documents | 8 |
| Masters of duplicates | 296 |
| Deleted items | 554 |
| Outcomes | 0 |
| Import filter | RIS |
| Is deleted? | false |

| Source name | **Social Policy and Practice** |
| --- | --- |
| Database name/platform | **Social Policy and Practice (OVID)** |
| Date of search | Jan 31, 2025 |
| Date of import | Jan 31, 2025 |
| Number items | 219 |
| Duplicates | 53 |
| Description |  |
| Notes |  |
| Search string | Database: Social Policy and Practice <202501> Search Strategy: 1 (Weight not "birth weight").ti. (219) 2 ("anti-fat" or bodyweight or "obese" or "obesity" or "overweight" or "skinny" or "fatness" or "thinness" or "body image" or "body hatred" or "body positive" or "thin ideal*" or "fat ideal*").af. (2573) 3 ("body esteem" and ("weight" or height or fat or thin)).af. (2) 4 ((body adj2 ideal*) or (body adj2 shape) or (body adj2 shapes) or (body adj1 size)).af. (64) 5 ((body adj10 weight) not (body adj10 "birth weight")).af. (175) 6 ("underweight" not "birth weight").af. (49) 7 ((weight adj1 bias) or (weight adj1 biases)).af. (5) 8 ("weight change" or "weight changes" or (weight adj2 gain*) or (weight adj2 loss) or "lose weight" or "losing weight" or (weight adj1 (reduc* or manage* or control*))).af. (436) 9 ("weight adj1 measurement*" or "height adj1 measurement*").af. (0) 10 ("healthy weight" or "unhealthy weight" or "weight adj5 monitor*" or "height adj5 monitor*").af. (112) 11 ("being measured" and ("weight" or weighing or height)).af. (0) 12 (weighing adj3 experiences).af. (0) 13 ("being fat" or "being weighed" or "Being thin" or "Being tall" or "Being short").af. (15) 14 (weight screening* or height screening*).af. (1) 15 ((stereotype or stereotypes or Stigma or stigmati* or ideals or ideal or discriminate* or discrimination* or "peer pressure" or prejudice* or bully* or teasing or cyberbull* or bullied) adj5 ("fat" or weight or thin or height or muscle* or muscular* or body)).af. (78) 16 ((fat or weight or thin or body or height or muscle or muscular*) adj5 (acceptability or satisfaction or satisfied or dissatisfaction or dissatisfied or anxiety or anxious or angst or peer pressure or feelings or worries or worry or concern or concerns or "over concern" or emotions or emotion or concerns or "self-worth" or "self esteem" or esteem or "self-concept" or "self-identification" or "pre-occupation" or "preoccupation" or "acceptability" or perceiv* or understand* or perception* or internalis* or attitud*)).af. (327) 17 (Body adj1 (dysmorph* or dismorph*)).af. (17) 18 ((body or fat or thin or height or weight) adj3 (shame or phobia*)).af. (1) 19 ((weight adj3 appearance) or (height adj3 appearance)).af. (9) 20 ("young people*" or adolescent* or youth or pubertal or pubescent or "pre adolescent" or "Pre pubescent" or "pre pubertal" or teen* or preteen* or tweens or tweenage* or youth or youths or schoolboy* or schoolgirl* or "school aged" or "young person*" or juvenile* or "Boy" or "boys" or "child" or "children*" or "child's" or "Girl" or "girls" or "Minors" or "preadolescent" or "Prepubescent" or "schoolchild*" or pubescent or "early adolescent*").af. (145043) 21 1 or 2 or 3 or 4 or 5 or 6 or 7 or 8 or 9 or 10 or 11 or 12 or 13 or 14 or 15 or 16 or 17 or 18 or 19 (3160) 22 20 and 21 (1539) 23 ("experiences" or "lived experience*" or "perceptions" or "perspective" or "perspectives" or "experience" or "attitude" or "attitudes" or "attitudinal" or "belief" or "beliefs" or "discourse" or "discourses" or "life-world" or "opinions" or "perceived" or "perception" or "standpoint" or "standpoints" or "understanding" or "understandings" or "viewpoint" or "viewpoints" or "views" or "voice" or "voices" or talk*).ti. (31691) 24 ((Girls or Boys or Child* or teen* or adolescen* or "young people*" or schoolchild* or pupil* or student*) adj2 ("perspective*" or "experience*" or "lived experience*" or "attitude" or "attitudes" or "attitudinal" or "belief" or "beliefs" or "discourse" or "discourses" or "life-world" or "opinions" or "standpoint" or "standpoints" or "understanding" or "understandings" or "viewpoint" or "viewpoints" or "views" or "voice" or "voices" or discussed or described* or drawings or drew)).ab. (11693) 25 ("audiorecording" or "ethnograph*" or "ethnolog*" or "ethno psycholog*" or "ethnopsycholog*" or "Focus Groups" or "Focus Group" or "repertory grid" or "stories" or "audio record" or "audio recorded" or "audio recorder" or "audio recording" or "audio recordings" or "audio records" or "thematic analysis" or "phenomenol*" or "grounded theory" or "grounded studies" or "grounded research" or "purposive sampling" or "biographical method" or "theoretical sampl*" or "conversation analysis" or "theoretical saturation" or "mixed design" or "mixed method" or "mixed methods" or "qualitative" or "interviewed" or "interviewing" or "interviewer" or "narratives" or "Diary entries" or "diary study" or Diaries or journalling or (Question* adj5 (semi-structured or semistructured or unstructured or informal or in-depth or indepth or open or "open ended")) or "Group discussion*" or "interviews" or "interview").af. (48552) 26 23 or 24 or 25 (76922) 27 22 and 26 (274) 28 limit 27 to yr="1863 - 2007" (55) 29 27 not 28 (219) ________________________________________ |
| Items coded | 166 |
| Uploaded documents | 9 |
| Masters of duplicates | 49 |
| Deleted items | 53 |
| Outcomes | 0 |
| Import filter | RIS |
| Is deleted? | false |

| Source name | **HMIC** |
| --- | --- |
| Database name/platform | **HEALTH MANAGEMENT INFORMATION CONSORTIUM-HMIC (OVID)** |
| Date of search | Jan 31, 2025 |
| Date of import | Jan 31, 2025 |
| Number items | 218 |
| Duplicates | 70 |
| Description |  |
| Notes |  |
| Search string | Database: HMIC Health Management Information Consortium <1979 to November 2024> Search Strategy: 1 (Weight not "birth weight").ti. (803) 2 ("anti-fat" or bodyweight or "obese" or "obesity" or "overweight" or "skinny" or "fatness" or "thinness" or "body image" or "body hatred" or "body positive" or "thin ideal*" or "fat ideal*").af. (5477) 3 ("body esteem" and ("weight" or height or fat or thin)).af. (2) 4 ((body adj2 ideal*) or (body adj2 shape) or (body adj2 shapes) or (body adj1 size)).af. (133) 5 ((body adj10 weight) not (body adj10 "birth weight")).af. (1074) 6 ("underweight" not "birth weight").af. (168) 7 ((weight adj1 bias) or (weight adj1 biases)).af. (7) 8 ("weight change" or "weight changes" or (weight adj2 gain*) or (weight adj2 loss) or "lose weight" or "losing weight" or (weight adj1 (reduc* or manage* or control*))).af. (1509) 9 ("weight adj1 measurement*" or "height adj1 measurement*").af. (0) 10 ("healthy weight" or "unhealthy weight" or "weight adj5 monitor*" or "height adj5 monitor*").af. (180) 11 ("being measured" and ("weight" or weighing or height)).af. (3) 12 (weighing adj3 experiences).af. (0) 13 ("being fat" or "being weighed" or "Being thin" or "Being tall" or "Being short").af. (14) 14 (weight screening* or height screening*).af. (3) 15 ((stereotype or stereotypes or Stigma or stigmati* or ideals or ideal or discriminate* or discrimination* or "peer pressure" or prejudice* or bully* or teasing or cyberbull* or bullied) adj5 ("fat" or weight or thin or height or muscle* or muscular* or body)).af. (68) 16 ((fat or weight or thin or body or height or muscle or muscular*) adj5 (acceptability or satisfaction or satisfied or dissatisfaction or dissatisfied or anxiety or anxious or angst or peer pressure or feelings or worries or worry or concern or concerns or "over concern" or emotions or emotion or concerns or "self-worth" or "self esteem" or esteem or "self-concept" or "self-identification" or "pre-occupation" or "preoccupation" or "acceptability" or perceiv* or understand* or perception* or internalis* or attitud*)).af. (349) 17 (Body adj1 (dysmorph* or dismorph*)).af. (10) 18 ((body or fat or thin or height or weight) adj3 (shame or phobia*)).af. (1) 19 ((weight adj3 appearance) or (height adj3 appearance)).af. (3) 20 ("young people*" or adolescent* or youth or pubertal or pubescent or "pre adolescent" or "Pre pubescent" or "pre pubertal" or teen* or preteen* or tweens or tweenage* or youth or youths or schoolboy* or schoolgirl* or "school aged" or "young person*" or juvenile* or "Boy" or "boys" or "child" or "children*" or "child's" or "Girl" or "girls" or "Minors" or "preadolescent" or "Prepubescent" or "schoolchild*" or pubescent or "early adolescent*").af. (44267) 21 1 or 2 or 3 or 4 or 5 or 6 or 7 or 8 or 9 or 10 or 11 or 12 or 13 or 14 or 15 or 16 or 17 or 18 or 19 (6499) 22 20 and 21 (2214) 23 ("experiences" or "lived experience*" or "perceptions" or "perspective" or "perspectives" or "experience" or "attitude" or "attitudes" or "attitudinal" or "belief" or "beliefs" or "discourse" or "discourses" or "life-world" or "opinions" or "perceived" or "perception" or "standpoint" or "standpoints" or "understanding" or "understandings" or "viewpoint" or "viewpoints" or "views" or "voice" or "voices" or talk*).ti. (19977) 24 ((Girls or Boys or Child* or teen* or adolescen* or "young people*" or schoolchild* or pupil* or student*) adj2 ("perspective*" or "experience*" or "lived experience*" or "attitude" or "attitudes" or "attitudinal" or "belief" or "beliefs" or "discourse" or "discourses" or "life-world" or "opinions" or "standpoint" or "standpoints" or "understanding" or "understandings" or "viewpoint" or "viewpoints" or "views" or "voice" or "voices" or discussed or described* or drawings or drew)).ab. (1888) 25 ("audiorecording" or "ethnograph*" or "ethnolog*" or "ethno psycholog*" or "ethnopsycholog*" or "Focus Groups" or "Focus Group" or "repertory grid" or "stories" or "audio record" or "audio recorded" or "audio recorder" or "audio recording" or "audio recordings" or "audio records" or "thematic analysis" or "phenomenol*" or "grounded theory" or "grounded studies" or "grounded research" or "purposive sampling" or "biographical method" or "theoretical sampl*" or "conversation analysis" or "theoretical saturation" or "mixed design" or "mixed method" or "mixed methods" or "qualitative" or "interviewed" or "interviewing" or "interviewer" or "narratives" or "Diary entries" or "diary study" or Diaries or journalling or (Question* adj5 (semi-structured or semistructured or unstructured or informal or in-depth or indepth or open or "open ended")) or "Group discussion*" or "interviews" or "interview").af. (29300) 26 23 or 24 or 25 (44595) 27 22 and 26 (300) 28 limit 27 to yr="1863 - 2007" (82) 29 27 not 28 (218) |
| Items coded | 148 |
| Uploaded documents | 2 |
| Masters of duplicates | 39 |
| Deleted items | 70 |
| Outcomes | 0 |
| Import filter | RIS |
| Is deleted? | false |

| Source name | **SSCI-ESCI-1** |
| --- | --- |
| Database name/platform | **Social Sciences Citation Index and Emerging Sources Citation Index (Web of Science Core Collection)** |
| Date of search | Jan 30, 2025 |
| Date of import | Jan 31, 2025 |
| Number items | 1000 |
| Duplicates | 562 |
| Description |  |
| Notes |  |
| Search string | # Web of Science Search Strategy (v0.1)  # Database: Web of Science Core Collection  # Entitlements:  - WOS.IC: 1993 to 2025 - WOS.CCR: 1985 to 2025 - WOS.SCI: 1900 to 2025 - WOS.AHCI: 1975 to 2025 - WOS.BHCI: 2005 to 2025 - WOS.BSCI: 2005 to 2025 - WOS.ESCI: 2005 to 2025 - WOS.ISTP: 1990 to 2025 - WOS.SSCI: 1900 to 2025 - WOS.ISSHP: 1990 to 2025   # Searches:  1: (TS=(body NEAR/10 weight) NOT TS=(body NEAR/10 "birth weight")) OR TS=((stereotype or stereotypes or Stigma or stigmati* or ideals or ideal or discriminate* or discrimination* or prejudice* or bully* or teasing or cyberbull* or bullied) NEAR/5 ("fat" or weight or thin or height or muscle* or muscular* or body)) Editions: WOS.SSCI,WOS.ESCI Date Run: Thu Jan 30 2025 11:50:25 GMT+0000 (Greenwich Mean Time) Results: 55941  2: TS=((fat or weight or thin or body or height or muscle or muscular*) NEAR/5 (acceptability or satisfaction or satisfied or dissatisfaction or dissatisfied or anxiety or anxious or angst or "peer pressure" or feelings or worries or worry or concern or concerns or "over concern" or emotions or emotion)) Editions: WOS.SSCI,WOS.ESCI Date Run: Thu Jan 30 2025 11:51:14 GMT+0000 (Greenwich Mean Time) Results: 19777  3: TS=((fat or weight or thin or body or height or muscle or muscular*) NEAR/5 (concerns or "self-worth" or "self esteem" or esteem or "self-concept" or "self-identification" or "pre-occupation" or "preoccupation" or "acceptability" or perceiv* or understand* or perception* or internalis* or attitud*)) Editions: WOS.SSCI,WOS.ESCI Date Run: Thu Jan 30 2025 11:51:39 GMT+0000 (Greenwich Mean Time) Results: 25783  4: TI=(Weight NOT "birth weight") OR TS=("anti-fat" or bodyweight or "obese" or "obesity" or "overweight" or "skinny" or "fatness" or "thinness" or "body image" or "body hatred" or "body positive" or "thin ideal*" or "fat ideal*") OR TS=("body esteem" and ("weight" or height or fat or thin)) OR TS=(body NEAR/2 ideal*) or TS=(body NEAR/2 shape) or TS=(body NEAR/2 shapes) or TS=(body NEAR/1 size) OR TS=("underweight" NOT "birth weight") OR TS=(weight NEAR/1 bias) or TS=(weight NEAR/1 biases) OR TS=("weight change" or "weight changes") or TS=(weight NEAR/2 gain*) or TS=(weight NEAR/2 loss) or TS=("lose weight" or "losing weight") or TS=(weight NEAR/1 (reduc* or manage* or control*)) OR TS=("weight NEAR/1 measurement*" or "height NEAR/1 measurement*") OR TS=("healthy weight" or "unhealthy weight") or TS=("weight NEAR/5 monitor*" or "height NEAR/5 monitor*") OR TS=("being measured" and ("weight" or weighing or height)) OR TS=(weighing NEAR/3 experiences) OR TS=(weight screening* or height screening*) OR TS=("being fat" or "being weighed" or "Being thin" or "Being tall" or "Being short") OR TS=(Body NEAR/1 (dysmorph* or dismorph*)) OR TS=((body or fat or thin or height or weight) NEAR/3 (shame or phobia*)) OR TS=(weight NEAR/3 appearance) or TS=(height NEAR/3 appearance) Editions: WOS.SSCI,WOS.ESCI Date Run: Thu Jan 30 2025 11:51:49 GMT+0000 (Greenwich Mean Time) Results: 241649  5: #4 OR #3 OR #2 OR #1 Editions: WOS.SSCI,WOS.ESCI Date Run: Thu Jan 30 2025 11:51:57 GMT+0000 (Greenwich Mean Time) Results: 277332  6: TS=("young people*" or adolescent* or youth or pubertal or pubescent or "pre adolescent" or "Pre pubescent" or "pre pubertal" or teen* or preteen* OR tweens or tweenage* or youth or youths or schoolboy* or schoolgirl* or "school aged" or "young person*" or juvenile* or "Boy" or "boys" or "child" or "children*" or "child's" or "Girl" or "girls" or "Minors" or "preadolescent" or "Prepubescent" or "schoolchild*" or pubescent or "early adolescent*") Editions: WOS.SSCI,WOS.ESCI Date Run: Thu Jan 30 2025 11:52:15 GMT+0000 (Greenwich Mean Time) Results: 1433556  7: TI=("experiences" or "lived experience*" or "perceptions" or "perspective" or "perspectives" or "experience" or "attitude" or "attitudes" or "attitudinal" or "belief" or "beliefs" or "discourse" or "discourses" or "life-world" or "opinions" or "perceived" or "perception" or "standpoint" or "standpoints" or "understanding" or "understandings" or "viewpoint" or "viewpoints" or "views" or "voice" or "voices" or talk*) Editions: WOS.SSCI,WOS.ESCI Date Run: Thu Jan 30 2025 11:52:31 GMT+0000 (Greenwich Mean Time) Results: 1060382  8: AB=((Girls or Boys or Child* or teen* or adolescen* or "young people*" or schoolchild* or pupil* or student*) NEAR/2 ("perspective*" or "experience*" or "lived experience*" or "attitude" or "attitudes" or "attitudinal" or "belief" or "beliefs" or "discourse" or "discourses" or "life-world" or "opinions" or "standpoint" or "standpoints" or "understanding" or "understandings" or "viewpoint" or "viewpoints" or "views" or "voice" or "voices" or discussed or described* or drawings or drew)) Editions: WOS.SSCI,WOS.ESCI Date Run: Thu Jan 30 2025 11:52:40 GMT+0000 (Greenwich Mean Time) Results: 159079  9: TS=("audiorecording" or "ethnograph*" or "ethnolog*" or "ethno psycholog*" or "ethnopsycholog*" or "Focus Groups" or "Focus Group" or "repertory grid" or "stories" or "audio record" or "audio recorded" or "audio recorder" or "audio recording" or "audio recordings" or "audio records" or "thematic analysis" or "phenomenol*" or "grounded theory" or "grounded studies" or "grounded research" or "purposive sampling" or "biographical method" or "theoretical sampl*" or "conversation analysis" or "theoretical saturation" or "mixed design" or "mixed method" or "mixed methods" or "qualitative" or "interviewed" or "interviewing" or "interviewer" or "narratives" or "Diary entries" or "diary study" or Diaries or journalling or (Question* NEAR/5 ("semi structured" or semistructured or unstructured or informal or in-depth or indepth or open or "open ended")) or "Group discussion*" or ("interviews" or "interview" NOT ("structured interview*")) OR "semi structured interview*") Editions: WOS.SSCI,WOS.ESCI Date Run: Thu Jan 30 2025 11:52:49 GMT+0000 (Greenwich Mean Time) Results: 1248037  10: #9 OR #8 OR #7 Editions: WOS.SSCI,WOS.ESCI Date Run: Thu Jan 30 2025 11:53:00 GMT+0000 (Greenwich Mean Time) Results: 2152527  11: #10 AND #5 AND #6 Editions: WOS.SSCI,WOS.ESCI Date Run: Thu Jan 30 2025 11:53:12 GMT+0000 (Greenwich Mean Time) Results: 14331  12: #11 Editions: WOS.SSCI,WOS.ESCI Timespan: 2008-01-01 to 2025-12-01 Date Run: Thu Jan 30 2025 11:54:14 GMT+0000 (Greenwich Mean Time) Results: 12644  13: TS=("National Health Service*" or NHS*) or (OO=(National Health Service) or OG=(NHS*) or AD=(National Health Service or NHS*)) and (AD=(Britain* or UK or "United Kingdom*" or England* or "North Ireland*" or "Northern Ireland*" or Scotland* or Wales*)) Editions: WOS.SSCI,WOS.ESCI Timespan: 2008-01-01 to 2025-12-01 Date Run: Thu Jan 30 2025 11:58:06 GMT+0000 (Greenwich Mean Time) Results: 83705  14: TI=(English not ((published or publication* or translat* or written or language* or speak* or literature or citation*) NEAR/5 English)) or AB=(English not ((published or publication* or translat* or written or language* or speak* or literature or citation*) NEAR/5 English)) Editions: WOS.SSCI,WOS.ESCI Timespan: 2008-01-01 to 2025-12-01 Date Run: Thu Jan 30 2025 11:58:16 GMT+0000 (Greenwich Mean Time) Results: 87832  15: TS=(GB or Britain* or (British* not "British Columbia") or UK or "United Kingdom*" or (England* not "New England") or "Northern Ireland*" or "Northern Irish*" or "North Ireland*" or "North Irish*" or Scotland* or Scottish* or ((Wales or "South Wales") not "New South Wales") or Welsh*) or SO=(Britain* or British* or UK or "United Kingdom*" or England* or "Northern Ireland*" or "Northern Irish*" or Scotland* or Scottish* or Wales* or Welsh*) or CU=(Britain* or UK or "United Kingdom*" or England* or "North Ireland*" or "Northern Ireland*" or Scotland* or Wales*) Editions: WOS.SSCI,WOS.ESCI Timespan: 2008-01-01 to 2025-12-01 Date Run: Thu Jan 30 2025 11:58:26 GMT+0000 (Greenwich Mean Time) Results: 1132568  16: TI=(Bath* or (Birmingham* not Alabama*) or Bradford* or Brighton* or Bristol* or Carlisle* or (Cambridge* not (Massachusetts* or Boston* or Harvard*)) or (Canterbury* not Zealand*) or Chelmsford* or Chester* or Chichester* or Coventry* or Derby* or (Durham* not (Carolina* or NC)) or Ely* or Exeter* or Gloucester* or Hereford* or Hull* or Lancaster* or Leeds* or Leicester* or (Lincoln* not Nebraska*) or (Liverpool* not ("New South Wales*" or NSW)) or (London* not (Ontario* or ONT or Toronto*)) or Manchester* or (Newcastle* not ("New South Wales*" or NSW)) or Norwich* or Nottingham* or Oxford* or Peterborough* or Plymouth* or Portsmouth* or Preston* or Ripon* or Salford* or Salisbury* or Sheffield* or Southampton* or "St Alban*" or Stoke* or Sunderland* or Truro* or Wakefield* or Wells or Westminster* or Winchester* or Wolverhampton* or (Worcester* not (Massachusetts* or Boston* or Harvard*)) or (York* not ("New York*" or NY or Ontario* or ONT or Toronto*))) or AB=(Bath* or (Birmingham* not Alabama*) or Bradford* or Brighton* or Bristol* or Carlisle* or (Cambridge* not (Massachusetts* or Boston* or Harvard*)) or (Canterbury* not Zealand*) or Chelmsford* or Chester* or Chichester* or Coventry* or Derby* or (Durham* not (Carolina* or NC)) or Ely* or Exeter* or Gloucester* or Hereford* or Hull* or Lancaster* or Leeds* or Leicester* or (Lincoln* not Nebraska*) or (Liverpool* not ("New South Wales*" or NSW)) or (London* not (Ontario* or ONT or Toronto*)) or Manchester* or (Newcastle* not ("New South Wales*" or NSW)) or Norwich* or Nottingham* or Oxford* or Peterborough* or Plymouth* or Portsmouth* or Preston* or Ripon* or Salford* or Salisbury* or Sheffield* or Southampton* or "St Alban*" or Stoke* or Sunderland* or Truro* or Wakefield* or Wells or Westminster* or Winchester* or Wolverhampton* or (Worcester* not (Massachusetts* or Boston* or Harvard*)) or (York* not ("New York*" or NY or Ontario* or ONT or Toronto*))) or AD=(Bath* or (Birmingham* and (Midlands* or England* or UK or "United Kingdom*")) or Bradford* or Brighton* or Bristol* or Carlisle* or (Cambridge* and (Cambridgeshire* or England* or UK or "United Kingdom*")) or (Canterbury* and (Kent* or England* or UK or "United Kingdom*")) or Chelmsford* or Chester* or Chichester* or Coventry* or Derby* or (Durham* and ("County Durham*" or England* or UK or "United Kingdom*")) or Ely* or Exeter* or Gloucester* or Hereford* or Hull* or Lancaster* or Leeds* or Leicester* or (Lincoln* and (Lincolnshire* or England* or UK or "United Kingdom*")) or (Liverpool* and (Merseyside* or England* or UK or "United Kingdom*")) or (London* and (Borough* or Westminster* or "Kingston-upon-Thames" or Kensington* or Chelsea* or Greenwich* or England* or UK or "United Kingdom*")) or Manchester* or (Newcastle* and (Tyne* or Wear* or England* or UK or "United Kingdom*")) or Norwich* or Nottingham* or Oxford* or Peterborough* or Plymouth* or Portsmouth* or Preston* or Ripon* or Salford* or Salisbury* or Sheffield* or Southampton* or ("St Alban*" and (Hertfordshire* or England* or UK or "United Kingdom*")) or Stoke* or Sunderland* or Truro* or Wakefield* or Wells or Westminster* or Winchester* or Wolverhampton* or (Worcester* and (Worcestershire* or England* or UK or "United Kingdom*")) or (York* and (Yorkshire* or England* or UK or "United Kingdom*"))) Editions: WOS.SSCI,WOS.ESCI Date Run: Thu Jan 30 2025 11:58:49 GMT+0000 (Greenwich Mean Time) Results: 1371754  17: TI=(Bath* or (Birmingham* not Alabama*) or Bradford* or Brighton* or Bristol* or Carlisle* or (Cambridge* not (Massachusetts* or Boston* or Harvard*)) or (Canterbury* not Zealand*) or Chelmsford* or Chester* or Chichester* or Coventry* or Derby* or (Durham* not (Carolina* or NC)) or Ely* or Exeter* or Gloucester* or Hereford* or Hull* or Lancaster* or Leeds* or Leicester* or (Lincoln* not Nebraska*) or (Liverpool* not ("New South Wales*" or NSW)) or (London* not (Ontario* or ONT or Toronto*)) or Manchester* or (Newcastle* not ("New South Wales*" or NSW)) or Norwich* or Nottingham* or Oxford* or Peterborough* or Plymouth* or Portsmouth* or Preston* or Ripon* or Salford* or Salisbury* or Sheffield* or Southampton* or "St Alban*" or Stoke* or Sunderland* or Truro* or Wakefield* or Wells or Westminster* or Winchester* or Wolverhampton* or (Worcester* not (Massachusetts* or Boston* or Harvard*)) or (York* not ("New York*" or NY or Ontario* or ONT or Toronto*))) or AB=(Bath* or (Birmingham* not Alabama*) or Bradford* or Brighton* or Bristol* or Carlisle* or (Cambridge* not (Massachusetts* or Boston* or Harvard*)) or (Canterbury* not Zealand*) or Chelmsford* or Chester* or Chichester* or Coventry* or Derby* or (Durham* not (Carolina* or NC)) or Ely* or Exeter* or Gloucester* or Hereford* or Hull* or Lancaster* or Leeds* or Leicester* or (Lincoln* not Nebraska*) or (Liverpool* not ("New South Wales*" or NSW)) or (London* not (Ontario* or ONT or Toronto*)) or Manchester* or (Newcastle* not ("New South Wales*" or NSW)) or Norwich* or Nottingham* or Oxford* or Peterborough* or Plymouth* or Portsmouth* or Preston* or Ripon* or Salford* or Salisbury* or Sheffield* or Southampton* or "St Alban*" or Stoke* or Sunderland* or Truro* or Wakefield* or Wells or Westminster* or Winchester* or Wolverhampton* or (Worcester* not (Massachusetts* or Boston* or Harvard*)) or (York* not ("New York*" or NY or Ontario* or ONT or Toronto*))) or AD=(Bath* or (Birmingham* and (Midlands* or England* or UK or "United Kingdom*")) or Bradford* or Brighton* or Bristol* or Carlisle* or (Cambridge* and (Cambridgeshire* or England* or UK or "United Kingdom*")) or (Canterbury* and (Kent* or England* or UK or "United Kingdom*")) or Chelmsford* or Chester* or Chichester* or Coventry* or Derby* or (Durham* and ("County Durham*" or England* or UK or "United Kingdom*")) or Ely* or Exeter* or Gloucester* or Hereford* or Hull* or Lancaster* or Leeds* or Leicester* or (Lincoln* and (Lincolnshire* or England* or UK or "United Kingdom*")) or (Liverpool* and (Merseyside* or England* or UK or "United Kingdom*")) or (London* and (Borough* or Westminster* or "Kingston-upon-Thames" or Kensington* or Chelsea* or Greenwich* or England* or UK or "United Kingdom*")) or Manchester* or (Newcastle* and (Tyne* or Wear* or England* or UK or "United Kingdom*")) or Norwich* or Nottingham* or Oxford* or Peterborough* or Plymouth* or Portsmouth* or Preston* or Ripon* or Salford* or Salisbury* or Sheffield* or Southampton* or ("St Alban*" and (Hertfordshire* or England* or UK or "United Kingdom*")) or Stoke* or Sunderland* or Truro* or Wakefield* or Wells or Westminster* or Winchester* or Wolverhampton* or (Worcester* and (Worcestershire* or England* or UK or "United Kingdom*")) or (York* and (Yorkshire* or England* or UK or "United Kingdom*"))) Editions: WOS.SSCI,WOS.ESCI Date Run: Thu Jan 30 2025 11:58:56 GMT+0000 (Greenwich Mean Time) Results: 1371754  18: TI=(Aberdeen* or Dundee* or Edinburgh* or Glasgow* or Inverness or (Perth* not Australia*) or Stirling*) or AB=(Aberdeen* or Dundee* or Edinburgh* or Glasgow* or Inverness or (Perth* not Australia*) or Stirling*) or AD=(Aberdeen* or Dundee* or Edinburgh* or Glasgow* or Inverness or (Perth* and (Perthshire* or Scotland* or UK or "United Kingdom*")) or Stirling*) Editions: WOS.SSCI,WOS.ESCI Date Run: Thu Jan 30 2025 11:59:27 GMT+0000 (Greenwich Mean Time) Results: 169726  19: TI=(Armagh* or Belfast* or Lisburn* or Londonderry* or Derry* or Newry*) or AB=(Armagh* or Belfast* or Lisburn* or Londonderry* or Derry* or Newry*) or AD=(Armagh* or Belfast* or Lisburn* or Londonderry* or Derry* or Newry*) Editions: WOS.SSCI,WOS.ESCI Date Run: Thu Jan 30 2025 11:59:35 GMT+0000 (Greenwich Mean Time) Results: 28102  20: TI=("South Holland" or Aldershot or Ashfield or Barking or Barnet or Barnsley or Bedfordshire or Bexley or Birkenhead or Blackburn or Blackpool or Bolton or Bournemouth or Brent or Bridgend or Bromley or Bromwich or Buckinghamshire or Burnley or Camberwell or Cambridgeshire or Camden or Chelsea or Chelsea or Cheshire or Cleveland or Colchester or Cornwall or Crawley or Croydon or Cumbria or Dagenham or Dartford or Derbyshire or Devon or Doncaster or Dorset or Dudley or Ealing or Ealing or Eastbourne or Enfield or Essex or Farnborough or Fulham or Furness or Galloway or Gateshead or Glamorgan or Glasgow or Gloucestershire or Gravesham or Greenwich or Grimsby or Guildford or Hackney or Hamlets or Hammersmith or Hampshire or Haringey or Haringey or Harrow or Hartlepool or Harwell or Hastings or Havering or Helens or Hertfordshire or Highland or Hillingdon or Hounslow or Hounslow or Hove or Huddersfield or Humber or Ipswich or Islington or Kensington or Kent or Kingston or Kirklees or Knowsley or Lambeth or Lancashire or Leicestershire or Lewisham or Lichfield or Lincoln or Lincolnshire or Loughborough or Luton or Lynn or Mansfield or Merseyside or Merton or Middlesbrough or Midlands or Milton Keynes or Newcastle or Newham or Norfolk or Northampton or Northamptonshire or Northumberland or Nottinghamshire or Oadby or Oldham or Oxfordshire or Poole or Portsmouth or Reading or Redbridge or Redcar or Richmond or Rochdale or Rotherham or Rushmoor or Sandwell or Scarborough or Scilly or Shropshire or Slough or Solihull or Somerset or Southampton or Southend or Southwark or Staffordshire or Stockport or Stockton or Suffolk or Surrey or Sussex or Sutton or Swindon or Teesside or Telford or Thurrock or Tower Hamlets or Tyne or Tyneside or Walsall or Waltham or Wandsworth or Warrington or Warwickshire or Watford or Wigan or Wight or Wigston or Wiltshire or Wirral or Woking or Worcestershire or Worthing or Yorkshire) Editions: WOS.SSCI,WOS.ESCI Date Run: Thu Jan 30 2025 12:29:55 GMT+0000 (Greenwich Mean Time) Results: 91646  21: AB=("South Holland" or Aldershot or Ashfield or Barking or Barnet or Barnsley or Bedfordshire or Bexley or Birkenhead or Blackburn or Blackpool or Bolton or Bournemouth or Brent or Bridgend or Bromley or Bromwich or Buckinghamshire or Burnley or Camberwell or Cambridgeshire or Camden or Chelsea or Chelsea or Cheshire or Cleveland or Colchester or Cornwall or Crawley or Croydon or Cumbria or Dagenham or Dartford or Derbyshire or Devon or Doncaster or Dorset or Dudley or Ealing or Ealing or Eastbourne or Enfield or Essex or Farnborough or Fulham or Furness or Galloway or Gateshead or Glamorgan or Glasgow or Gloucestershire or Gravesham or Greenwich or Grimsby or Guildford or Hackney or Hamlets or Hammersmith or Hampshire or Haringey or Haringey or Harrow or Hartlepool or Harwell or Hastings or Havering or Helens or Hertfordshire or Highland or Hillingdon or Hounslow or Hounslow or Hove or Huddersfield or Humber or Ipswich or Islington or Kensington or Kent or Kingston or Kirklees or Knowsley or Lambeth or Lancashire or Leicestershire or Lewisham or Lichfield or Lincoln or Lincolnshire or Loughborough or Luton or Lynn or Mansfield or Merseyside or Merton or Middlesbrough or Midlands or Milton Keynes or Newcastle or Newham or Norfolk or Northampton or Northamptonshire or Northumberland or Nottinghamshire or Oadby or Oldham or Oxfordshire or Poole or Portsmouth or Reading or Redbridge or Redcar or Richmond or Rochdale or Rotherham or Rushmoor or Sandwell or Scarborough or Scilly or Shropshire or Slough or Solihull or Somerset or Southampton or Southend or Southwark or Staffordshire or Stockport or Stockton or Suffolk or Surrey or Sussex or Sutton or Swindon or Teesside or Telford or Thurrock or Tower Hamlets or Tyne or Tyneside or Walsall or Waltham or Wandsworth or Warrington or Warwickshire or Watford or Wigan or Wight or Wigston or Wiltshire or Wirral or Woking or Worcestershire or Worthing or Yorkshire) Editions: WOS.SSCI,WOS.ESCI Date Run: Thu Jan 30 2025 12:30:02 GMT+0000 (Greenwich Mean Time) Results: 165329  22: AD=("South Holland" or Aldershot or Ashfield or Barking or Barnet or Barnsley or Bedfordshire or Bexley or Birkenhead or Blackburn or Blackpool or Bolton or Bournemouth or Brent or Bridgend or Bromley or Bromwich or Buckinghamshire or Burnley or Camberwell or Cambridgeshire or Camden or Chelsea or Chelsea or Cheshire or Cleveland or Colchester or Cornwall or Crawley or Croydon or Cumbria or Dagenham or Dartford or Derbyshire or Devon or Doncaster or Dorset or Dudley or Ealing or Ealing or Eastbourne or Enfield or Essex or Farnborough or Fulham or Furness or Galloway or Gateshead or Glamorgan or Glasgow or Gloucestershire or Gravesham or Greenwich or Grimsby or Guildford or Hackney or Hamlets or Hammersmith or Hampshire or Haringey or Haringey or Harrow or Hartlepool or Harwell or Hastings or Havering or Helens or Hertfordshire or Highland or Hillingdon or Hounslow or Hounslow or Hove or Huddersfield or Humber or Ipswich or Islington or Kensington or Kent or Kingston or Kirklees or Knowsley or Lambeth or Lancashire or Leicestershire or Lewisham or Lichfield or Lincoln or Lincolnshire or Loughborough or Luton or Lynn or Mansfield or Merseyside or Merton or Middlesbrough or Midlands or Milton Keynes or Newcastle or Newham or Norfolk or Northampton or Northamptonshire or Northumberland or Nottinghamshire or Oadby or Oldham or Oxfordshire or Poole or Portsmouth or Reading or Redbridge or Redcar or Richmond or Rochdale or Rotherham or Rushmoor or Sandwell or Scarborough or Scilly or Shropshire or Slough or Solihull or Somerset or Southampton or Southend or Southwark or Staffordshire or Stockport or Stockton or Suffolk or Surrey or Sussex or Sutton or Swindon or Teesside or Telford or Thurrock or Tower Hamlets or Tyne or Tyneside or Walsall or Waltham or Wandsworth or Warrington or Warwickshire or Watford or Wigan or Wight or Wigston or Wiltshire or Wirral or Woking or Worcestershire or Worthing or Yorkshire) Editions: WOS.SSCI,WOS.ESCI Date Run: Thu Jan 30 2025 12:30:11 GMT+0000 (Greenwich Mean Time) Results: 929060  23: #22 OR #21 OR #20 OR #19 OR #18 OR #17 OR #16 OR #15 OR #14 OR #13 Editions: WOS.SSCI,WOS.ESCI Date Run: Thu Jan 30 2025 12:30:41 GMT+0000 (Greenwich Mean Time) Results: 2307385  24: #23 AND #12 Editions: WOS.SSCI,WOS.ESCI Date Run: Thu Jan 30 2025 12:31:38 GMT+0000 (Greenwich Mean Time) Results: 2315  25: #12 Editions: WOS.SSCI,WOS.ESCI Date Run: Thu Jan 30 2025 12:32:00 GMT+0000 (Greenwich Mean Time) Results: 12644  26: #12 and USA or AUSTRALIA or CANADA or SPAIN or BRAZIL or ITALY or PEOPLES R CHINA or GERMANY or SWEDEN or NETHERLANDS or NORWAY or FRANCE or INDIA or TURKEY or BELGIUM or SOUTH KOREA or ISRAEL or MEXICO or IRAN or POLAND or SOUTH AFRICA or IRELAND or DENMARK or NEW ZEALAND or TAIWAN or PORTUGAL or SWITZERLAND or MALAYSIA or FINLAND or SAUDI ARABIA or CHILE or GREECE or INDONESIA or RUSSIA or JAPAN or PAKISTAN or SINGAPORE or TURKIYE or COLOMBIA or AUSTRIA or THAILAND or GHANA or KUWAIT or ROMANIA or ARGENTINA or EGYPT or CROATIA or PERU or U ARAB EMIRATES or VIETNAM or BANGLADESH or CZECH REPUBLIC or LEBANON or UGANDA or HUNGARY or JORDAN or KENYA or NIGERIA or LITHUANIA or MOROCCO or SLOVAKIA or ETHIOPIA or FIJI or QATAR or SLOVENIA or TUNISIA or UKRAINE or BAHRAIN or BOSNIA HERCEG or ECUADOR or MALAWI or PALESTINE or SERBIA or BARBADOS or BELARUS or DEM REP CONGO or GEORGIA or GUATEMALA or ICELAND or JAMAICA or LIBYA or LUXEMBOURG or MALI or MOZAMBIQUE or OMAN or PARAGUAY or SRI LANKA or TONGA or URUGUAY or VENEZUELA or ALGERIA or ARMENIA or AZERBAIJAN or BOLIVIA or BOTSWANA or BURKINA FASO or CAMEROON or COSTA RICA or CYPRUS or ESTONIA or ESWATINI or GUINEA or HAITI or IRAQ or KAZAKHSTAN or LATVIA or MALTA or NEPAL or NEW CALEDONIA or PHILIPPINES or SENEGAL or SEYCHELLES or SOMALIA or ST VINCENT or SYRIA or TANZANIA or TRINIDAD TOBAGO or ZAMBIA (Exclude – Countries/Regions) Editions: WOS.SSCI,WOS.ESCI Date Run: Thu Jan 30 2025 12:35:04 GMT+0000 (Greenwich Mean Time) Results: 835  27: #26 OR #24 Editions: WOS.SSCI,WOS.ESCI Date Run: Thu Jan 30 2025 12:35:23 GMT+0000 (Greenwich Mean Time) Results: 2379 |
| Items coded | 438 |
| Uploaded documents | 12 |
| Masters of duplicates | 125 |
| Deleted items | 562 |
| Outcomes | 0 |
| Import filter | RIS |
| Is deleted? | false |

| Source name | **IBSS** |
| --- | --- |
| Database name/platform |  |
| Date of search | Jan 30, 2025 |
| Date of import | Jan 31, 2025 |
| Number items | 771 |
| Duplicates | 155 |
| Description |  |
| Notes |  |
| Search string | Re-ran line S35 of ASSIA strategy as thesaurus is the same |
| Items coded | 616 |
| Uploaded documents | 4 |
| Masters of duplicates | 165 |
| Deleted items | 155 |
| Outcomes | 0 |
| Import filter | RIS |
| Is deleted? | false |

| Source name | **ASSIA** |
| --- | --- |
| Database name/platform | **ASSIA (ProQuest)** |
| Date of search | Jan 30, 2025 |
| Date of import | Jan 31, 2025 |
| Number items | 1219 |
| Duplicates | 393 |
| Description |  |
| Notes |  |
| Search string | S1 TI,AB("young people*" or adolescent* or youth or pubertal or pubescent or "pre adolescent" or "Pre pubescent" or "pre pubertal" or teen* or preteen* OR tweens or tweenage* or youth or youths or schoolboy* or schoolgirl* or "school aged" or "young person*" or juvenile* or "Boy" or "boys" or "child" or "children*" or "child's" or "Girl" or "girls" or "Minors" or "preadolescent" or "Prepubescent" or "schoolchild*" or pubescent or "early adolescent*") Applied Social Sciences Index & Abstracts (ASSIA) 290268 S2 MAINSUBJECT.EXACT("Children") OR MAINSUBJECT.EXACT("Adolescents") OR MAINSUBJECT.EXACT("Adolescent girls") OR MAINSUBJECT.EXACT("Adolescent boys") OR MAINSUBJECT.EXACT("Childhood obesity") OR MAINSUBJECT.EXACT("Youth") Applied Social Sciences Index & Abstracts (ASSIA) 121416 S6 TI,AB(body NEAR/10 weight) NOT TI,AB(body NEAR/10 "birth weight") Applied Social Sciences Index & Abstracts (ASSIA) 3927 S7 TI(Weight NOT "birth weight") OR TI,AB("anti-fat" or bodyweight or "obese" or "obesity" or "overweight" or "skinny" or "fatness" or "thinness" or "body image" or "body hatred" or "body positive" or "thin ideal*" or "fat ideal*") OR TI,AB("body esteem" and ("weight" or height or fat or thin)) OR TI,AB(body NEAR/2 ideal*) or TI,AB(body NEAR/2 shape) or TI,AB(body NEAR/2 shapes) or TI,AB(body NEAR/1 size) OR TI,AB("underweight" NOT "birth weight") OR TI,AB(weight NEAR/1 bias) or TI,AB(weight NEAR/1 biases) OR TI,AB("weight change" or "weight changes") or TI,AB(weight NEAR/2 gain*) or TI,AB(weight NEAR/2 loss) or TI,AB("lose weight" or "losing weight") or TI,AB(weight NEAR/1 (reduc* or manage* or control*)) OR TI,AB("weight NEAR/1 measurement*" or "height NEAR/1 measurement*") OR TI,AB("healthy weight" or "unhealthy weight") or TI,AB("weight NEAR/5 monitor*" or "height NEAR/5 monitor*") OR TI,AB("being measured" and ("weight" or weighing or height)) OR TI,AB(weighing NEAR/3 experiences) OR TI,AB(weight screening* or height screening*) OR TI,AB("being fat" or "being weighed" or "Being thin" or "Being tall" or "Being short") OR TI,AB((stereotype or stereotypes or Stigma or stigmati* or ideals or ideal or discriminate* or discrimination* or prejudice* or bully* or teasing or cyberbull* or bullied) NEAR/5 ("fat" or weight or thin or height or muscle* or muscular* or body)) OR TI,AB((fat or weight or thin or body or height or muscle or muscular*) NEAR/5 (acceptability or satisfaction or satisfied or dissatisfaction or dissatisfied or anxiety or anxious or angst or "peer pressure" or feelings or worries or worry or concern or concerns or "over concern" or emotions or emotion)) or TI,AB((fat or weight or thin or body or height or muscle or muscular*) NEAR/5 (concerns or "self-worth" or "self esteem" or esteem or "self-concept" or "self-identification" or "pre-occupation" or "preoccupation" or "acceptability" or perceiv* or understand* or perception* or internalis* or attitud*)) OR TI,AB(Body NEAR/1 (dysmorph* or dismorph*)) OR TI,AB((body or fat or thin or height or weight) NEAR/3 (shame or phobia*)) OR TI,AB(weight NEAR/3 appearance) or TI,AB(height NEAR/3 appearance) Applied Social Sciences Index & Abstracts (ASSIA) 26040 S9 AB((Girls or Boys or Child* or teen* or adolescen* or "young people*" or schoolchild* or pupil* or student*) NEAR/2 ("perspective*" or "experience*" or "lived experience*" or "attitude" or "attitudes" or "attitudinal" or "belief" or "beliefs" or "discourse" or "discourses" or "life-world" or "opinions" or "standpoint" or "standpoints" or "understanding" or "understandings" or "viewpoint" or "viewpoints" or "views" or "voice" or "voices" or discussed or described* or drawings or drew)) Applied Social Sciences Index & Abstracts (ASSIA) 36223 S10 TI,AB("audiorecording" or "ethnograph*" or "ethnolog*" or "ethno psycholog*" or "ethnopsycholog*" or "Focus Groups" or "Focus Group" or "repertory grid" or "stories" or "audio record" or "audio recorded" or "audio recorder" or "audio recording" or "audio recordings" or "audio records" or "thematic analysis" or "phenomenol*" or "grounded theory" or "grounded studies" or "grounded research" or "purposive sampling" or "biographical method" or "theoretical sampl*" or "conversation analysis" or "theoretical saturation" or "mixed design" or "mixed method" or "mixed methods" or "qualitative" or "interviewed" or "interviewing" or "interviewer" or "narratives" or "Diary entries" or "diary study" or Diaries or journalling or (Question* NEAR/5 ("semi structured" or semistructured or unstructured or informal or in-depth or indepth or open or "open ended")) or "Group discussion*" or ("interviews" or "interview" NOT ("structured interview*")) OR "semi structured interview*") Applied Social Sciences Index & Abstracts (ASSIA) 194773 S12 [S1] OR [S2] Applied Social Sciences Index & Abstracts (ASSIA) 296098 S18 TI("experiences" or ("lived experience" OR "lived experiences") or "perceptions" or "perspective" or "perspectives" or "experience" or "attitude" or "attitudes" or "attitudinal" or "belief" or "beliefs" or "discourse" or "discourses" or "life-world" or "opinions" or "perceived" or "perception" or "standpoint" or "standpoints" or "understanding" or "understandings" or "viewpoint" or "viewpoints" or "views" or "voice" or "voices" or talk*) AND TI(("young people" OR "young peoples") or adolescent* or youth or pubertal or pubescent or "pre adolescent" or "Pre pubescent" or "pre pubertal" or teen* or preteen* OR tweens or tweenage* or youth or youths or schoolboy* or schoolgirl* or "school aged" or ("young person" OR "young persons") or juvenile* or "Boy" or "boys" or "child" or "children*" or "child's" or "Girl" or "girls" or "Minors" or "preadolescent" or "Prepubescent" or "schoolchild*" or pubescent or ("early adolescent" OR "early adolescents")) Applied Social Sciences Index & Abstracts (ASSIA) 21006 S29 MAINSUBJECT.EXACT("Ethnography") OR MAINSUBJECT.EXACT("Urban ethnography") OR MAINSUBJECT.EXACT("Focus groups") OR MAINSUBJECT.EXACT("Grounded theory") OR MAINSUBJECT.EXACT("Qualitative research") OR MAINSUBJECT.EXACT("Interpretative phenomenological analysis") OR MAINSUBJECT.EXACT("Opinions") OR MAINSUBJECT.EXACT("Interviews") OR MAINSUBJECT.EXACT("Beliefs") OR MAINSUBJECT.EXACT("Semistructured questionnaires") OR MAINSUBJECT.EXACT("Phenomenology") OR MAINSUBJECT.EXACT("Personal experiences") OR MAINSUBJECT.EXACT("Narratives") OR MAINSUBJECT.EXACT("Life history") OR MAINSUBJECT.EXACT("Attitudes") Applied Social Sciences Index & Abstracts (ASSIA) 100860 S30 MAINSUBJECT.EXACT("Weight gain") OR MAINSUBJECT.EXACT("Obesity") OR MAINSUBJECT.EXACT("Weight loss") OR MAINSUBJECT.EXACT("Body weight") OR MAINSUBJECT.EXACT("Height") OR MAINSUBJECT.EXACT("Body size") OR MAINSUBJECT.EXACT("Body shape") OR MAINSUBJECT.EXACT("Childhood obesity") OR MAINSUBJECT.EXACT("Body image") OR MAINSUBJECT.EXACT("Thinness") Applied Social Sciences Index & Abstracts (ASSIA) 18235 S32 [S9] OR [S10] OR [S18] OR [S29] Applied Social Sciences Index & Abstracts (ASSIA) 265579 S33 [S6] OR [S7] OR [S30] Applied Social Sciences Index & Abstracts (ASSIA) 29531 S34 [S12] AND [S32] AND [S33] Applied Social Sciences Index & Abstracts (ASSIA) 2271 S35 ([S12] AND [S32] AND [S33]) AND pd(20080101-20251231) Applied Social Sciences Index & Abstracts (ASSIA) 1794 S37 ([S12] AND [S32] AND [S33]) NOT (location.exact("United States--US" OR "Australia" OR "China" OR "New Zealand" OR "Canada" OR "India" OR "California" OR "Netherlands" OR "Denmark" OR "Israel" OR "Mexico" OR "New York" OR "Scotland" OR "Norway" OR "Spain" OR "Sweden" OR "Uganda" OR "Africa" OR "Ethiopia" OR "Queensland Australia" OR "Taiwan" OR "Turkey" OR "Bangladesh" OR "France" OR "Georgia" OR "Germany" OR "Ghana" OR "North Carolina" OR "Saudi Arabia" OR "South Korea" OR "Thailand" OR "Kansas" OR "Kenya" OR "Los Angeles California" OR "Malawi" OR "Malaysia" OR "Massachusetts" OR "Minnesota" OR "Mississippi" OR "Portugal" OR "South Africa" OR "South Carolina" OR "Western Australia Australia" OR "Aachen Germany" OR "Alaska" OR "Baltimore Maryland" OR "Bangkok Thailand" OR "Belgium" OR "Brazil" OR "Chicago Illinois" OR "Florida" OR "Indonesia" OR "Iran" OR "Lebanon" OR "Melbourne Victoria Australia" OR "Midwest states" OR "Minneapolis Minnesota" OR "Mumbai India" OR "Nebraska" OR "New York City New York" OR "Nigeria" OR "Pacific Northwest" OR "South Australia Australia" OR "South Dakota" OR "Southern California" OR "Sydney New South Wales Australia" OR "Tanzania" OR "Texas" OR "Utah" OR "Victoria Australia" OR "Addis Ababa Ethiopia" OR "Alabama" OR "Alberta Canada" OR "Albuquerque New Mexico" OR "Appalachia" OR "Appalachia Virginia" OR "Atlanta Georgia" OR "Brisbane Queensland Australia" OR "Bronx New York" OR "Brooklyn New York" OR "Cambodia" OR "Central Valley" OR "Colorado" OR "Congo-Democratic Republic of Congo" OR "Connecticut" OR "Cook County Illinois" OR "Cyprus" OR "East Asia" OR "Egypt" OR "Finland" OR "Guinea Bissau" OR "Hong Kong" OR "Hong Kong China" OR "Isan Thailand") AND pd(20080101-20251231)) Applied Social Sciences Index & Abstracts (ASSIA) 1367 S38 ([S12] AND [S32] AND [S33]) AND (location.exact("United Kingdom--UK" OR "England" OR "Wales" OR "Bristol England" OR "Glasgow Scotland") AND pd(20080101-20251231)) Applied Social Sciences Index & Abstracts (ASSIA) 57 Saved search lines S38 and S37 and de-duped in Endnote = 1,219 records |
| Items coded | 826 |
| Uploaded documents | 14 |
| Masters of duplicates | 213 |
| Deleted items | 393 |
| Outcomes | 0 |
| Import filter | RIS |
| Is deleted? | false |

| Source name | **CINAHL** |
| --- | --- |
| Database name/platform | **CINAHL (EBSCO)** |
| Date of search | Jan 31, 2025 |
| Date of import | Jan 31, 2025 |
| Number items | 2229 |
| Duplicates | 1086 |
| Description |  |
| Notes |  |
| Search string | # Query Limiters/Expanders Last Run Via Results S32 S30 OR S31 Expanders - Apply equivalent subjects Search modes - Proximity Interface - EBSCOhost Research Databases Search Screen - Advanced Search Database - CINAHL Plus 2,229 S31 S6 AND S26 Expanders - Apply equivalent subjects Search modes - Proximity Interface - EBSCOhost Research Databases Search Screen - Advanced Search Database - CINAHL Plus 565 S30 S26 NOT S29 Expanders - Apply equivalent subjects Search modes - Proximity Interface - EBSCOhost Research Databases Search Screen - Advanced Search Database - CINAHL Plus 2,136 S29 S27 NOT S28 Expanders - Apply equivalent subjects Search modes - Proximity Interface - EBSCOhost Research Databases Search Screen - Advanced Search Database - CINAHL Plus Display S28 (MH "Europe") OR (MH "United Kingdom+") Expanders - Apply equivalent subjects Search modes - Proximity Interface - EBSCOhost Research Databases Search Screen - Advanced Search Database - CINAHL Plus Display S27 (MH "Africa+") OR (MH "Americas+") OR (MH "Asia+") OR (MH "Australia+") OR (MH "Atlantic Islands+") OR (MH "Indian Ocean Islands+") OR (MH "Pacific Islands+") Expanders - Apply equivalent subjects Search modes - Proximity Interface - EBSCOhost Research Databases Search Screen - Advanced Search Database - CINAHL Plus Display S26 s25 Limiters - Publication Year: 2008-2025 Expanders - Apply equivalent subjects Search modes - Proximity Interface - EBSCOhost Research Databases Search Screen - Advanced Search Database - CINAHL Plus 3,335 S25 S15 AND S24 Expanders - Apply equivalent subjects Search modes - Proximity Interface - EBSCOhost Research Databases Search Screen - Advanced Search Database - CINAHL Plus 3,911 S24 S5 OR S6 OR S14 OR S16 OR S17 OR S18 OR S19 OR S20 OR S21 OR S22 OR S23 Expanders - Apply equivalent subjects Search modes - Proximity Interface - EBSCOhost Research Databases Search Screen - Advanced Search Database - CINAHL Plus Display S23 TI ( ("South Holland" or Aldershot or Ashfield or Barking or Barnet or Barnsley or Bedfordshire or Bexley or Birkenhead or Blackburn or Blackpool or Bolton or Bournemouth or Brent or Bridgend or Bromley or Bromwich or Buckinghamshire or Burnley or Camberwell or Cambridgeshire or Camden or Chelsea or Chelsea or Cheshire or Cleveland or Colchester or Cornwall or Crawley or Croydon or Cumbria or Dagenham or Dartford or Derbyshire or Devon or Doncaster or Dorset or Dudley or Ealing or Ealing or Eastbourne or Enfield or Essex or Farnborough or Fulham or Furness or Galloway or Gateshead or Glamorgan or Glasgow or Gloucestershire or Gravesham or Greenwich or Grimsby or Guildford or Hackney or Hamlets or Hammersmith or Hampshire or Haringey or Haringey or Harrow or Hartlepool or Harwell or Hastings or Havering or Helens or Hertfordshire or Highland or Hillingdon or Hounslow or Hounslow or Hove or Huddersfield or Humber or Ipswich or Islington or Kensington or Kent or Kingston or Kirklees or Knowsley or Lambeth or Lancashire or Leicestershire or Lewisham or Lichfield or Lincoln or Lincolnshire or Loughborough or Luton or Lynn or Mansfield or Merseyside or Merton or Middlesbrough or Midlands or Milton Keynes or Newcastle or Newham or Norfolk or Northampton or Northamptonshire or Northumberland or Nottinghamshire or Oadby or Oldham or Oxfordshire or Poole or Portsmouth or Reading or Redbridge or Redcar or Richmond or Rochdale or Rotherham or Rushmoor or Sandwell or Scarborough or Scilly or Shropshire or Slough or Solihull or Somerset or Southampton or Southend or Southwark or Staffordshire or Stockport or Stockton or Suffolk or Surrey or Sussex or Sutton or Swindon or Teesside or Telford or Thurrock or Tower Hamlets or Tyne or Tyneside or Walsall or Waltham or Wandsworth or Warrington or Warwickshire or Watford or Wigan or Wight or Wigston or Wiltshire or Wirral or Woking or Worcestershire or Worthing or Yorkshire). ) OR AB ( ("South Holland" or Aldershot or Ashfield or Barking or Barnet or Barnsley or Bedfordshire or Bexley or Birkenhead or Blackburn or Blackpool or Bolton or Bournemouth or Brent or Bridgend or Bromley or Bromwich or Buckinghamshire or Burnley or Camberwell or Cambridgeshire or Camden or Chelsea or Chelsea or Cheshire or Cleveland or Colchester or Cornwall or Crawley or Croydon or Cumbria or Dagenham or Dartford or Derbyshire or Devon or Doncaster or Dorset or Dudley or Ealing or Ealing or Eastbourne or Enfield or Essex or Farnborough or Fulham or Furness or Galloway or Gateshead or Glamorgan or Glasgow or Gloucestershire or Gravesham or Greenwich or Grimsby or Guildford or Hackney or Hamlets or Hammersmith or Hampshire or Haringey or Haringey or Harrow or Hartlepool or Harwell or Hastings or Havering or Helens or Hertfordshire or Highland or Hillingdon or Hounslow or Hounslow or Hove or Huddersfield or Humber or Ipswich or Islington or Kensington or Kent or Kingston or Kirklees or Knowsley or Lambeth or Lancashire or Leicestershire or Lewisham or Lichfield or Lincoln or Lincolnshire or Loughborough or Luton or Lynn or Mansfield or Merseyside or Merton or Middlesbrough or Midlands or Milton Keynes or Newcastle or Newham or Norfolk or Northampton or Northamptonshire or Northumberland or Nottinghamshire or Oadby or Oldham or Oxfordshire or Poole or Portsmouth or Reading or Redbridge or Redcar or Richmond or Rochdale or Rotherham or Rushmoor or Sandwell or Scarborough or Scilly or Shropshire or Slough or Solihull or Somerset or Southampton or Southend or Southwark or Staffordshire or Stockport or Stockton or Suffolk or Surrey or Sussex or Sutton or Swindon or Teesside or Telford or Thurrock or Tower Hamlets or Tyne or Tyneside or Walsall or Waltham or Wandsworth or Warrington or Warwickshire or Watford or Wigan or Wight or Wigston or Wiltshire or Wirral or Woking or Worcestershire or Worthing or Yorkshire). ) OR AF ( ("South Holland" or Aldershot or Ashfield or Barking or Barnet or Barnsley or Bedfordshire or Bexley or Birkenhead or Blackburn or Blackpool or Bolton or Bournemouth or Brent or Bridgend or Bromley or Bromwich or Buckinghamshire or Burnley or Camberwell or Cambridgeshire or Camden or Chelsea or Chelsea or Cheshire or Cleveland or Colchester or Cornwall or Crawley or Croydon or Cumbria or Dagenham or Dartford or Derbyshire or Devon or Doncaster or Dorset or Dudley or Ealing or Ealing or Eastbourne or Enfield or Essex or Farnborough or Fulham or Furness or Galloway or Gateshead or Glamorgan or Glasgow or Gloucestershire or Gravesham or Greenwich or Grimsby or Guildford or Hackney or Hamlets or Hammersmith or Hampshire or Haringey or Haringey or Harrow or Hartlepool or Harwell or Hastings or Havering or Helens or Hertfordshire or Highland or Hillingdon or Hounslow or Hounslow or Hove or Huddersfield or Humber or Ipswich or Islington or Kensington or Kent or Kingston or Kirklees or Knowsley or Lambeth or Lancashire or Leicestershire or Lewisham or Lichfield or Lincoln or Lincolnshire or Loughborough or Luton or Lynn or Mansfield or Merseyside or Merton or Middlesbrough or Midlands or Milton Keynes or Newcastle or Newham or Norfolk or Northampton or Northamptonshire or Northumberland or Nottinghamshire or Oadby or Oldham or Oxfordshire or Poole or Portsmouth or Reading or Redbridge or Redcar or Richmond or Rochdale or Rotherham or Rushmoor or Sandwell or Scarborough or Scilly or Shropshire or Slough or Solihull or Somerset or Southampton or Southend or Southwark or Staffordshire or Stockport or Stockton or Suffolk or Surrey or Sussex or Sutton or Swindon or Teesside or Telford or Thurrock or Tower Hamlets or Tyne or Tyneside or Walsall or Waltham or Wandsworth or Warrington or Warwickshire or Watford or Wigan or Wight or Wigston or Wiltshire or Wirral or Woking or Worcestershire or Worthing or Yorkshire). ) Expanders - Apply equivalent subjects Search modes - Proximity Interface - EBSCOhost Research Databases Search Screen - Advanced Search Database - CINAHL Plus Display S22 TI ( (bangor or "bangor's" or cardiff or "cardiff's" or newport or "newport's" or "st asaph" or "st asaph's" or "st davids" or swansea or "swansea's"). ) OR AB ( (bangor or "bangor's" or cardiff or "cardiff's" or newport or "newport's" or "st asaph" or "st asaph's" or "st davids" or swansea or "swansea's"). ) OR AF ( (bangor or "bangor's" or cardiff or "cardiff's" or newport or "newport's" or "st asaph" or "st asaph's" or "st davids" or swansea or "swansea's"). ) Expanders - Apply equivalent subjects Search modes - Proximity Interface - EBSCOhost Research Databases Search Screen - Advanced Search Database - CINAHL Plus Display S21 TI ( ("Isle of Man" or "Channel Islands" or "Guernsey") ) OR AB ( ("Isle of Man" or "Channel Islands" or "Guernsey") ) OR AF ( ("Isle of Man" or "Channel Islands" or "Guernsey") ) Expanders - Apply equivalent subjects Search modes - Proximity Interface - EBSCOhost Research Databases Search Screen - Advanced Search Database - CINAHL Plus Display S20 TI ( (armagh or "armagh's" or belfast or "belfast's" or lisburn or "lisburn's" or londonderry or "londonderry's" or derry or "derry's" or newry or "newry's") ) OR AB ( (armagh or "armagh's" or belfast or "belfast's" or lisburn or "lisburn's" or londonderry or "londonderry's" or derry or "derry's" or newry or "newry's") ) OR AF ( (armagh or "armagh's" or belfast or "belfast's" or lisburn or "lisburn's" or londonderry or "londonderry's" or derry or "derry's" or newry or "newry's") ) Expanders - Apply equivalent subjects Search modes - Proximity Interface - EBSCOhost Research Databases Search Screen - Advanced Search Database - CINAHL Plus Display S19 TI ( (aberdeen or "aberdeen's" or dundee or "dundee's" or edinburgh or "edinburgh's" or glasgow or "glasgow's" or inverness or (perth not australia*) or ("perth's" not australia*) or stirling or "stirling's"). ) OR AB ( (aberdeen or "aberdeen's" or dundee or "dundee's" or edinburgh or "edinburgh's" or glasgow or "glasgow's" or inverness or (perth not australia*) or ("perth's" not australia*) or stirling or "stirling's"). ) OR AF ( (aberdeen or "aberdeen's" or dundee or "dundee's" or edinburgh or "edinburgh's" or glasgow or "glasgow's" or inverness or (perth not australia*) or ("perth's" not australia*) or stirling or "stirling's"). ) Expanders - Apply equivalent subjects Search modes - Proximity Interface - EBSCOhost Research Databases Search Screen - Advanced Search Database - CINAHL Plus Display S18 TI ( (bath or "bath's" or ((birmingham not alabama*) or ("birmingham's" not alabama*) or bradford or "bradford's" or brighton or "brighton's" or bristol or "bristol's" or carlisle* or "carlisle's" or (cambridge not (massachusetts* or boston* or harvard*)) or ("cambridge's" not (massachusetts* or boston* or harvard*)) or (canterbury not zealand*) or ("canterbury's" not zealand*) or chelmsford or "chelmsford's" or chester or "chester's" or chichester or "chichester's" or coventry or "coventry's" or derby or "derby's" or (durham not (carolina* or nc)) or ("durham's" not (carolina* or nc)) or ely or "ely's" or exeter or "exeter's" or gloucester or "gloucester's" or hereford or "hereford's" or hull or "hull's" or lancaster or "lancaster's" or leeds* or leicester or "leicester's" or (lincoln not nebraska*) or ("lincoln's" not nebraska*) or (liverpool not (new south wales* or nsw)) or ("liverpool's" not (new south wales* or nsw)) or ((london not (ontario* or ont or toronto*)) or ("london's" not (ontario* or ont or toronto*)) or manchester or "manchester's" or (newcastle not (new south wales* or nsw)) or ("newcastle's" not (new south wales* or nsw)) or norwich or "norwich's" or nottingham or "nottingham's" or oxford or "oxford's" or peterborough or "peterborough's" or plymouth or "plymouth's" or portsmouth or "portsmouth's" or preston or "preston's" or ripon or "ripon's" or salford or "salford's" or salisbury or "salisbury's" or sheffield or "sheffield's" or southampton or "southampton's" or st albans or stoke or "stoke's" or sunderland or "sunderland's" or truro or "truro's" or wakefield or "wakefield's" or wells or westminster or "westminster's" or winchester or "winchester's" or wolverhampton or "wolverhampton's" or (worcester not (massachusetts* or boston* or harvard*)) or ("worcester's" not (massachusetts* or boston* or harvard*)) or (york not ("new york*" or ny or ontario* or ont or toronto*)) or ("york's" not ("new york*" or ny or ontario* or ont or toronto*))))) ) OR AB ( (bath or "bath's" or ((birmingham not alabama*) or ("birmingham's" not alabama*) or bradford or "bradford's" or brighton or "brighton's" or bristol or "bristol's" or carlisle* or "carlisle's" or (cambridge not (massachusetts* or boston* or harvard*)) or ("cambridge's" not (massachusetts* or boston* or harvard*)) or (canterbury not zealand*) or ("canterbury's" not zealand*) or chelmsford or "chelmsford's" or chester or "chester's" or chichester or "chichester's" or coventry or "coventry's" or derby or "derby's" or (durham not (carolina* or nc)) or ("durham's" not (carolina* or nc)) or ely or "ely's" or exeter or "exeter's" or gloucester or "gloucester's" or hereford or "hereford's" or hull or "hull's" or lancaster or "lancaster's" or leeds* or leicester or "leicester's" or (lincoln not nebraska*) or ("lincoln's" not nebraska*) or (liverpool not (new south wales* or nsw)) or ("liverpool's" not (new south wales* or nsw)) or ((london not (ontario* or ont or toronto*)) or ("london's" not (ontario* or ont or toronto*)) or manchester or "manchester's" or (newcastle not (new south wales* or nsw)) or ("newcastle's" not (new south wales* or nsw)) or norwich or "norwich's" or nottingham or "nottingham's" or oxford or "oxford's" or peterborough or "peterborough's" or plymouth or "plymouth's" or portsmouth or "portsmouth's" or preston or "preston's" or ripon or "ripon's" or salford or "salford's" or salisbury or "salisbury's" or sheffield or "sheffield's" or southampton or "southampton's" or st albans or stoke or "stoke's" or sunderland or "sunderland's" or truro or "truro's" or wakefield or "wakefield's" or wells or westminster or "westminster's" or winchester or "winchester's" or wolverhampton or "wolverhampton's" or (worcester not (massachusetts* or boston* or harvard*)) or ("worcester's" not (massachusetts* or boston* or harvard*)) or (york not ("new york*" or ny or ontario* or ont or toronto*)) or ("york's" not ("new york*" or ny or ontario* or ont or toronto*))))) ) OR AF ( (bath or "bath's" or ((birmingham not alabama*) or ("birmingham's" not alabama*) or bradford or "bradford's" or brighton or "brighton's" or bristol or "bristol's" or carlisle* or "carlisle's" or (cambridge not (massachusetts* or boston* or harvard*)) or ("cambridge's" not (massachusetts* or boston* or harvard*)) or (canterbury not zealand*) or ("canterbury's" not zealand*) or chelmsford or "chelmsford's" or chester or "chester's" or chichester or "chichester's" or coventry or "coventry's" or derby or "derby's" or (durham not (carolina* or nc)) or ("durham's" not (carolina* or nc)) or ely or "ely's" or exeter or "exeter's" or gloucester or "gloucester's" or hereford or "hereford's" or hull or "hull's" or lancaster or "lancaster's" or leeds* or leicester or "leicester's" or (lincoln not nebraska*) or ("lincoln's" not nebraska*) or (liverpool not (new south wales* or nsw)) or ("liverpool's" not (new south wales* or nsw)) or ((london not (ontario* or ont or toronto*)) or ("london's" not (ontario* or ont or toronto*)) or manchester or "manchester's" or (newcastle not (new south wales* or nsw)) or ("newcastle's" not (new south wales* or nsw)) or norwich or "norwich's" or nottingham or "nottingham's" or oxford or "oxford's" or peterborough or "peterborough's" or plymouth or "plymouth's" or portsmouth or "portsmouth's" or preston or "preston's" or ripon or "ripon's" or salford or "salford's" or salisbury or "salisbury's" or sheffield or "sheffield's" or southampton or "southampton's" or st albans or stoke or "stoke's" or sunderland or "sunderland's" or truro or "truro's" or wakefield or "wakefield's" or wells or westminster or "westminster's" or winchester or "winchester's" or wolverhampton or "wolverhampton's" or (worcester not (massachusetts* or boston* or harvard*)) or ("worcester's" not (massachusetts* or boston* or harvard*)) or (york not ("new york*" or ny or ontario* or ont or toronto*)) or ("york's" not ("new york*" or ny or ontario* or ont or toronto*))))) ) Expanders - Apply equivalent subjects Search modes - Proximity Interface - EBSCOhost Research Databases Search Screen - Advanced Search Database - CINAHL Plus Display S17 TI ( (english not ((published or publication* or translat* or written or language* or speak* or literature or citation*) N4 english)) ) OR AB ( (english not ((published or publication* or translat* or written or language* or speak* or literature or citation*) N4 english)) ) Expanders - Apply equivalent subjects Search modes - Proximity Interface - EBSCOhost Research Databases Search Screen - Advanced Search Database - CINAHL Plus Display S16 TI ( ("district council" or "local council" or "local authorities" or "NHS Trust" or "primary care trust" or "borough council" or "county council" or "local authority" or "district councils" or "local councils" or "NHS Trusts" or "primary care trusts" or "borough councils" or "county councils" or "Social Care Trust" OR national health service* or nhs*)) OR AB ( ("district council" or "local council" or "local authorities" or "NHS Trust" or "primary care trust" or "borough council" or "county council" or "local authority" or "district councils" or "local councils" or "NHS Trusts" or "primary care trusts" or "borough councils" or "county councils" or "Social Care Trust" national health service* or nhs*)) OR AF ( ("district council" or "local council" or "local authorities" or "NHS Trust" or "primary care trust" or "borough council" or "county council" or "local authority" or "district councils" or "local councils" or "NHS Trusts" or "primary care trusts" or "borough councils" or "county councils" or "Social Care Trust" national health service* or nhs*) ) Expanders - Apply equivalent subjects Search modes - Proximity Interface - EBSCOhost Research Databases Search Screen - Advanced Search Database - CINAHL Plus Display S15 S13 Expanders - Apply equivalent subjects Search modes - Proximity Interface - EBSCOhost Research Databases Search Screen - Advanced Search Database - CINAHL Plus 12,398 S14 SO ( (gb or "g.b." or britain* or (british* not "british columbia") or uk or "u.k." or "united kingdom*" or (england* not "new england") or "northern ireland*" or irish* or scotland* or scottish* or ((wales or "south wales") not "new south wales") or welsh*) ) Expanders - Apply equivalent subjects Search modes - Proximity Interface - EBSCOhost Research Databases Search Screen - Advanced Search Database - CINAHL Plus Display S13 S10 AND S11 AND S12 Expanders - Apply equivalent subjects Search modes - Proximity Interface - EBSCOhost Research Databases Search Screen - Advanced Search Database - CINAHL Plus 12,398 S12 S3 OR S9 Expanders - Apply equivalent subjects Search modes - Proximity Interface - EBSCOhost Research Databases Search Screen - Advanced Search Database - CINAHL Plus 762,181 S11 S1 OR S7 Expanders - Apply equivalent subjects Search modes - Proximity Interface - EBSCOhost Research Databases Search Screen - Advanced Search Database - CINAHL Plus 297,491 S10 S2 OR S4 OR S8 Expanders - Apply equivalent subjects Search modes - Proximity Interface - EBSCOhost Research Databases Search Screen - Advanced Search Database - CINAHL Plus 1,312,465 S9 (SU "Qualitative Studies") OR (SU "Ethnographic Research") OR (SU "Ethnological Research") OR (SU "Ethnonursing Research") OR (SU "Grounded Theory") OR (SU "Phenomenological Research") OR (SU "Phenomenology") OR (SU "Narratives") OR (SU "Unstructured Interview") OR (SU "Semi-Structured Interview") OR (SU "Interviews") OR (SU "Audiorecording") OR (SU "Focus Groups") OR (SU "Life Experiences") OR (SU "Thematic Analysis") OR (SU "Attitude") OR (SU "Attitude to Obesity") OR (SU "Attitude to Health") Expanders - Apply equivalent subjects Search modes - Proximity Interface - EBSCOhost Research Databases Search Screen - Advanced Search Database - CINAHL Plus 563,956 S8 (SU "Child") OR (SU "Adolescence") Expanders - Apply equivalent subjects Search modes - Proximity Interface - EBSCOhost Research Databases Search Screen - Advanced Search Database - CINAHL Plus Display S7 (SU "Obesity") OR (SU "Attitude to Obesity") OR (SU "Weight Bias") OR (SU "Pediatric Obesity") OR (SU "Weight Gain") OR (SU "Body Weight") OR (SU "Body Weight Changes") OR (SU "Weight Loss") OR (SU "Thinness") OR (SU "Body Dysmorphic Disorder") OR (SU "Body Height") OR (SU "Body Dissatisfaction") OR (SU "Body Size") OR (SU "Body Image") OR (SU "Body Satisfaction") Expanders - Apply equivalent subjects Search modes - Proximity Interface - EBSCOhost Research Databases Search Screen - Advanced Search Database - CINAHL Plus Display S6 (SU "United Kingdom") OR (SU "Great Britain") OR (SU "England") OR (SU "Scotland") OR (SU "Wales") OR (SU"Northern Ireland") Expanders - Apply equivalent subjects Search modes - Proximity Interface - EBSCOhost Research Databases Search Screen - Advanced Search Database - CINAHL Plus Display S5 SU ( (gb or "g.b." or britain* or (british* not "british columbia") or uk or "u.k." or "united kingdom*" or (england* not "new england") or "northern ireland*" or irish* or scotland* or scottish* or ((wales or "south wales") not "new south wales") or welsh*) ) OR TI ( (english not ((published or publication* or translat* or written or language* or speak* or literature or citation*) adj5 english)). ) OR AB ( (english not ((published or publication* or translat* or written or language* or speak* or literature or citation*) adj5 english)) ) OR TI ( (gb or "g.b." or britain* or (british* not "british columbia") or uk or "u.k." or "united kingdom*" or (england* not "new england") or "northern ireland*" or irish* or scotland* or scottish* or ((wales or "south wales") not "new south wales") or welsh*) ) OR AB ( (gb or "g.b." or britain* or (british* not "british columbia") or uk or "u.k." or "united kingdom*" or (england* not "new england") or "northern ireland*" or irish* or scotland* or scottish* or ((wales or "south wales") not "new south wales") or welsh*) ) Expanders - Apply equivalent subjects Search modes - Proximity Interface - EBSCOhost Research Databases Search Screen - Advanced Search Database - CINAHL Plus Display S4 SO ( child* not (childbirth OR "child birth") ) OR SO adolescen* Expanders - Apply equivalent subjects Search modes - Proximity Interface - EBSCOhost Research Databases Search Screen - Advanced Search Database - CINAHL Plus Display S3 ( (TI("experiences" or "lived experience*" or "perceptions" or "perspective" or "perspectives" or "experience" or "attitude" or "attitudes" or "attitudinal" or "belief" or "beliefs" or "discourse" or "discourses" or "life-world" or "opinions" or "perceived" or "perception" or "standpoint" or "standpoints" or "understanding" or "understandings" or "viewpoint" or "viewpoints" or "views" or "voice" or "voices" or talk*) AND TI("young people*" or adolescent* or youth or pubertal or pubescent or "pre adolescent" or "Pre pubescent" or "pre pubertal" or teen* or preteen* OR tweens or tweenage* or youth or youths or schoolboy* or schoolgirl* or "school aged" or "young person*" or juvenile* or "Boy" or "boys" or "child" or "children*" or "child's" or "Girl" or "girls" or "Minors" or "preadolescent" or "Prepubescent" or "schoolchild*" or pubescent or "early adolescent*")) OR ( AB((Girls or Boys or Child* or teen* or adolescen* or "young people*" or schoolchild* or pupil* or student*) N2 ("perspective*" or "experience*" or "lived experience*" or "attitude" or "attitudes" or "attitudinal" or "belief" or "beliefs" or "discourse" or "discourses" or "life-world" or "opinions" or "standpoint" or "standpoints" or "understanding" or "understandings" or "viewpoint" or "viewpoints" or "views" or "voice" or "voices" or discussed or described* or drawings or drew)) ) OR ( AB((Girls or Boys or Child* or teen* or adolescen* or "young people*" or schoolchild* or pupil* or student*) N2 ("perspective*" or "experience*" or "lived experience*" or "attitude" or "attitudes" or "attitudinal" or "belief" or "beliefs" or "discourse" or "discourses" or "life-world" or "opinions" or "standpoint" or "standpoints" or "understanding" or "understandings" or "viewpoint" or "viewpoints" or "views" or "voice" or "voices" or discussed or described* or drawings or drew)) TI("audiorecording" or "ethnograph*" or "ethnolog*" or "ethno psycholog*" or "ethnopsycholog*" or "Focus Groups" or "Focus Group" or "repertory grid" or "stories" or "audio record" or "audio recorded" or "audio recorder" or "audio recording" or "audio recordings" or "audio records" or "thematic analysis" or "phenomenol*" or "grounded theory" or "grounded studies" or "grounded research" or "purposive sampling" or "biographical method" or "theoretical sampl*" or "conversation analysis" or "theoretical saturation" or "mixed design" or "mixed method" or "mixed methods" or "qualitative" or "interviewed" or "interviewing" or "interviewer" or "narratives" or "Diary entries" or "diary study" or Diaries or journalling or (Question* N5 (semi-structured or semistructured or unstructured or informal or in-depth or indepth or open or "open ended")) or "Group discussion*" or "interviews" or "interview") OR AB("audiorecording" or "ethnograph*" or "ethnolog*" or "ethno psycholog*" or "ethnopsycholog*" or "Focus Groups" or "Focus Group" or "repertory grid" or "stories" or "audio record" or "audio recorded" or "audio recorder" or "audio recording" or "audio recordings" or "audio records" or "thematic analysis" or "phenomenol*" or "grounded theory" or "grounded studies" or "grounded research" or "purposive sampling" or "biographical method" or "theoretical sampl*" or "conversation analysis" or "theoretical saturation" or "mixed design" or "mixed method" or "mixed methods" or "qualitative" or "interviewed" or "interviewing" or "interviewer" or "narratives" or "Diary entries" or "diary study" or Diaries or journalling or (Question* N5 (semi-structured or semistructured or unstructured or informal or in-depth or indepth or open or "open ended")) or "Group discussion*" or "interviews" or "interview") ) Expanders - Apply equivalent subjects Search modes - Proximity Interface - EBSCOhost Research Databases Search Screen - Advanced Search Database - CINAHL Plus 471,286 S2 TI("young people*" or adolescent* or youth or pubertal or pubescent or "pre adolescent" or "Pre pubescent" or "pre pubertal" or teen* or preteen* or tweens or tweenage* or youth or youths or schoolboy* or schoolgirl* or "school aged" or "young person*" or juvenile* or "Boy" or "boys" or "child" or "children*" or "child's" or "Girl" or "girls" or "Minors" or "preadolescent" or "Prepubescent" or "schoolchild*" or pubescent or "early adolescent*") OR AB("young people*" or adolescent* or youth or pubertal or pubescent or "pre adolescent" or "Pre pubescent" or "pre pubertal" or teen* or preteen* or tweens or tweenage* or youth or youths or schoolboy* or schoolgirl* or "school aged" or "young person*" or juvenile* or "Boy" or "boys" or "child" or "children*" or "child's" or "Girl" or "girls" or "Minors" or "preadolescent" or "Prepubescent" or "schoolchild*" or pubescent or "early adolescent*") Expanders - Apply equivalent subjects Search modes - Proximity Interface - EBSCOhost Research Databases Search Screen - Advanced Search Database - CINAHL Plus 747,591 S1 TI(Weight not "birth weight") OR TI("anti-fat" or bodyweight or "obese" or "obesity" or "overweight" or "skinny" or "fatness" or "thinness" or "body image" or "body hatred" or "body positive" or "thin ideal*" or "fat ideal*") OR AB("anti-fat" or bodyweight or "obese" or "obesity" or "overweight" or "skinny" or "fatness" or "thinness" or "body image" or "body hatred" or "body positive" or "thin ideal*" or "fat ideal*") OR TI("body esteem" and ("weight" or height or fat or thin)) OR AB("body esteem" and ("weight" or height or fat or thin)) OR TI((body N2 ideal*) or (body N2 shape) or (body N2 shapes) or (body N1 size)) OR AB((body N2 ideal*) or (body N2 shape) or (body N2 shapes) or (body N1 size)) OR TI(body N10 weight) OR AB(body N10 weight) OR TI("underweight" not "birth weight") OR AB("underweight" not "birth weight") OR TI((weight N1 bias) or (weight N1 biases)) OR AB((weight N1 bias) or (weight N1 biases)) OR TI("weight change" or "weight changes" or (weight N2 gain*) or (weight N2 loss) or "lose weight" or "losing weight" or (weight N1 (reduc* or manage* or control*))) OR AB("weight change" or "weight changes" or (weight N2 gain*) or (weight N2 loss) or "lose weight" or "losing weight" or (weight N1 (reduc* or manage* or control*))) OR TI("weight N1 measurement*" or "height N1 measurement*") OR AB("weight N1 measurement*" or "height N1 measurement*") OR TI("healthy weight" or "unhealthy weight" or "weight N5 monitor*" or "height N5 monitor*") OR AB("healthy weight" or "unhealthy weight" or "weight N5 monitor*" or "height N5 monitor*") OR TI("being measured" and ("weight" or weighing or height)) OR AB("healthy weight" or "unhealthy weight" or "weight N5 monitor*" or "height N5 monitor*") OR TI(weighing N3 experiences) OR AB(weighing N3 experiences) OR TI(weight screening* or height screening*) OR AB(weight screening* or height screening*) OR TI("being fat" or "being weighed" or "Being thin" or "Being tall" or "Being short") OR AB("being fat" or "being weighed" or "Being thin" or "Being tall" or "Being short") OR TI((stereotype or stereotypes or Stigma or stigmati* or ideals or ideal or discriminate* or discrimination* or "peer pressure" or prejudice* or bully* or teasing or cyberbull* or bullied) N5 ("fat" or weight or thin or height or muscle* or muscular* or body)) OR AB((stereotype or stereotypes or Stigma or stigmati* or ideals or ideal or discriminate* or discrimination* or "peer pressure" or prejudice* or bully* or teasing or cyberbull* or bullied) N5 ("fat" or weight or thin or height or muscle* or muscular* or body)) OR TI((fat or weight or thin or body or height or muscle or muscular*) N5 (acceptability or satisfaction or satisfied or dissatisfaction or dissatisfied or anxiety or anxious or angst or peer pressure or feelings or worries or worry or concern or concerns or "over concern" or emotions or emotion or concerns or "self-worth" or "self esteem" or esteem or "self-concept" or "self-identification" or "pre-occupation" or "preoccupation" or "acceptability" or perceiv* or understand* or perception* or internalis* or attitud*)) OR AB((fat or weight or thin or body or height or muscle or muscular*) N5 (acceptability or satisfaction or satisfied or dissatisfaction or dissatisfied or anxiety or anxious or angst or peer pressure or feelings or worries or worry or concern or concerns or "over concern" or emotions or emotion or concerns or "self-worth" or "self esteem" or esteem or "self-concept" or "self-identification" or "pre-occupation" or "preoccupation" or "acceptability" or perceiv* or understand* or perception* or internalis* or attitud*)) OR TI(Body N1 (dysmorph* or dismorph*)) OR AB(Body N1 (dysmorph* or dismorph*)) OR TI((body or fat or thin or height or weight) N3 (shame or phobia*)) OR AB((body or fat or thin or height or weight) N3 (shame or phobia*)) OR TI((weight N3 appearance) or (height N3 appearance)) OR AB((weight N3 appearance) or (height N3 appearance)) Expanders - Apply equivalent subjects Search modes - Proximity Interface - EBSCOhost Research Databases Search Screen - Advanced Search Database - CINAHL Plus 238,520 |
| Items coded | 1143 |
| Uploaded documents | 5 |
| Masters of duplicates | 86 |
| Deleted items | 1086 |
| Outcomes | 0 |
| Import filter | RIS |
| Is deleted? | false |

| Source name | **ERIC** |
| --- | --- |
| Database name/platform | **ERIC (EBSCO)** |
| Date of search | Jan 31, 2025 |
| Date of import | Jan 31, 2025 |
| Number items | 823 |
| Duplicates | 280 |
| Description |  |
| Notes |  |
| Search string | S15 S11 NOT S14 Expanders - Apply equivalent subjects Search modes - Proximity Interface - EBSCOhost Research Databases Search Screen - Advanced Search Database - ERIC 823 S14 S13 NOT S12 Expanders - Apply equivalent subjects Search modes - Proximity Interface - EBSCOhost Research Databases Search Screen - Advanced Search Database - ERIC 46 S13 s11 Limiters - Education Level: Adult Basic Education, Adult Education, Grade 11, Grade 12, Higher Education, Postsecondary Education, Preschool Education, Two Year Colleges Expanders - Apply equivalent subjects Search modes - Proximity Interface - EBSCOhost Research Databases Search Screen - Advanced Search Database - ERIC 101 S12 s11 Limiters - Education Level: Early Childhood Education, Elementary Education, Elementary Secondary Education, Grade 1, Grade 2, Grade 3, Grade 4, Grade 5, Grade 6, Grade 7, Grade 8, Grade 9, Grade 10, High Schools, Intermediate Grades, Junior High Schools, Kindergarten, Middle Schools, Primary Education, Secondary Education Expanders - Apply equivalent subjects Search modes - Proximity Interface - EBSCOhost Research Databases Search Screen - Advanced Search Database - ERIC 441 S11 S10 Limiters - Published Date: 20080101-20251231 Expanders - Apply equivalent subjects Search modes - Proximity Interface - EBSCOhost Research Databases Search Screen - Advanced Search Database - ERIC 869 S10 S7 AND S8 AND S9 Expanders - Apply equivalent subjects Search modes - Proximity Interface - EBSCOhost Research Databases Search Screen - Advanced Search Database - ERIC 1,264 S9 S3 OR S6 Expanders - Apply equivalent subjects Search modes - Proximity Interface - EBSCOhost Research Databases Search Screen - Advanced Search Database - ERIC 427,898 S8 S1 OR S5 Expanders - Apply equivalent subjects Search modes - Proximity Interface - EBSCOhost Research Databases Search Screen - Advanced Search Database - ERIC 10,423 S7 S2 OR S4 Expanders - Apply equivalent subjects Search modes - Proximity Interface - EBSCOhost Research Databases Search Screen - Advanced Search Database - ERIC 438,993 S6 DE "Adolescent Attitudes" OR DE "Childrens Attitudes" OR DE "Ethnology" OR DE "Ethnography" OR DE "Grounded Theory" OR DE "Interviews" OR DE "Semi Structured Interviews" OR DE "Personal Narratives" OR DE "Qualitative Research" OR DE "Discussion Groups" OR DE "Focus Groups" OR DE "Group Discussion" OR DE "Opinions" OR DE "Attitudes" Expanders - Apply equivalent subjects Search modes - Proximity Interface - EBSCOhost Research Databases Search Screen - Advanced Search Database - ERIC 147,281 S5 (DE "Obesity" OR DE "Body Height" OR DE "Body Weight") OR ((DE "Self Concept") AND TI (Weight OR body OR muscle)) OR ((DE "Self Concept") AND DE ("BODY COMPOSITION")) Expanders - Apply equivalent subjects Search modes - Proximity Interface - EBSCOhost Research Databases Search Screen - Advanced Search Database - ERIC 6,003 S4 DE "Young Children" OR DE "Childrens Attitudes" OR DE "Youth" OR DE "Adolescent Attitudes" OR DE "Adolescents" OR DE "Children" OR DE "Early Adolescents" OR DE "Preadolescents" Expanders - Apply equivalent subjects Search modes - Proximity Interface - EBSCOhost Research Databases Search Screen - Advanced Search Database - ERIC 136,645 S3 ( (TI("experiences" or "lived experience*" or "perceptions" or "perspective" or "perspectives" or "experience" or "attitude" or "attitudes" or "attitudinal" or "belief" or "beliefs" or "discourse" or "discourses" or "life-world" or "opinions" or "perceived" or "perception" or "standpoint" or "standpoints" or "understanding" or "understandings" or "viewpoint" or "viewpoints" or "views" or "voice" or "voices" or talk*) AND TI("young people*" or adolescent* or youth or pubertal or pubescent or "pre adolescent" or "Pre pubescent" or "pre pubertal" or teen* or preteen* OR tweens or tweenage* or youth or youths or schoolboy* or schoolgirl* or "school aged" or "young person*" or juvenile* or "Boy" or "boys" or "child" or "children*" or "child's" or "Girl" or "girls" or "Minors" or "preadolescent" or "Prepubescent" or "schoolchild*" or pubescent or "early adolescent*")) OR ( AB((Girls or Boys or Child* or teen* or adolescen* or "young people*" or schoolchild* or pupil* or student*) N2 ("perspective*" or "experience*" or "lived experience*" or "attitude" or "attitudes" or "attitudinal" or "belief" or "beliefs" or "discourse" or "discourses" or "life-world" or "opinions" or "standpoint" or "standpoints" or "understanding" or "understandings" or "viewpoint" or "viewpoints" or "views" or "voice" or "voices" or discussed or described* or drawings or drew)) ) OR ( AB((Girls or Boys or Child* or teen* or adolescen* or "young people*" or schoolchild* or pupil* or student*) N2 ("perspective*" or "experience*" or "lived experience*" or "attitude" or "attitudes" or "attitudinal" or "belief" or "beliefs" or "discourse" or "discourses" or "life-world" or "opinions" or "standpoint" or "standpoints" or "understanding" or "understandings" or "viewpoint" or "viewpoints" or "views" or "voice" or "voices" or discussed or described* or drawings or drew)) TI("audiorecording" or "ethnograph*" or "ethnolog*" or "ethno psycholog*" or "ethnopsycholog*" or "Focus Groups" or "Focus Group" or "repertory grid" or "stories" or "audio record" or "audio recorded" or "audio recorder" or "audio recording" or "audio recordings" or "audio records" or "thematic analysis" or "phenomenol*" or "grounded theory" or "grounded studies" or "grounded research" or "purposive sampling" or "biographical method" or "theoretical sampl*" or "conversation analysis" or "theoretical saturation" or "mixed design" or "mixed method" or "mixed methods" or "qualitative" or "interviewed" or "interviewing" or "interviewer" or "narratives" or "Diary entries" or "diary study" or Diaries or journalling or (Question* N5 (semi-structured or semistructured or unstructured or informal or in-depth or indepth or open or "open ended")) or "Group discussion*" or "interviews" or "interview") OR AB("audiorecording" or "ethnograph*" or "ethnolog*" or "ethno psycholog*" or "ethnopsycholog*" or "Focus Groups" or "Focus Group" or "repertory grid" or "stories" or "audio record" or "audio recorded" or "audio recorder" or "audio recording" or "audio recordings" or "audio records" or "thematic analysis" or "phenomenol*" or "grounded theory" or "grounded studies" or "grounded research" or "purposive sampling" or "biographical method" or "theoretical sampl*" or "conversation analysis" or "theoretical saturation" or "mixed design" or "mixed method" or "mixed methods" or "qualitative" or "interviewed" or "interviewing" or "interviewer" or "narratives" or "Diary entries" or "diary study" or Diaries or journalling or (Question* N5 (semi-structured or semistructured or unstructured or informal or in-depth or indepth or open or "open ended")) or "Group discussion*" or "interviews" or "interview") ) Expanders - Apply equivalent subjects Search modes - Proximity Interface - EBSCOhost Research Databases Search Screen - Advanced Search Database - ERIC 384,589 S2 TI("young people*" or adolescent* or youth or pubertal or pubescent or "pre adolescent" or "Pre pubescent" or "pre pubertal" or teen* or preteen* or tweens or tweenage* or youth or youths or schoolboy* or schoolgirl* or "school aged" or "young person*" or juvenile* or "Boy" or "boys" or "child" or "children*" or "child's" or "Girl" or "girls" or "Minors" or "preadolescent" or "Prepubescent" or "schoolchild*" or pubescent or "early adolescent*") OR AB("young people*" or adolescent* or youth or pubertal or pubescent or "pre adolescent" or "Pre pubescent" or "pre pubertal" or teen* or preteen* or tweens or tweenage* or youth or youths or schoolboy* or schoolgirl* or "school aged" or "young person*" or juvenile* or "Boy" or "boys" or "child" or "children*" or "child's" or "Girl" or "girls" or "Minors" or "preadolescent" or "Prepubescent" or "schoolchild*" or pubescent or "early adolescent*") Expanders - Apply equivalent subjects Search modes - Proximity Interface - EBSCOhost Research Databases Search Screen - Advanced Search Database - ERIC 423,417 S1 TI(Weight not "birth weight") OR TI("anti-fat" or bodyweight or "obese" or "obesity" or "overweight" or "skinny" or "fatness" or "thinness" or "body image" or "body hatred" or "body positive" or "thin ideal*" or "fat ideal*") OR AB("anti-fat" or bodyweight or "obese" or "obesity" or "overweight" or "skinny" or "fatness" or "thinness" or "body image" or "body hatred" or "body positive" or "thin ideal*" or "fat ideal*") OR TI("body esteem" and ("weight" or height or fat or thin)) OR AB("body esteem" and ("weight" or height or fat or thin)) OR TI((body N2 ideal*) or (body N2 shape) or (body N2 shapes) or (body N1 size)) OR AB((body N2 ideal*) or (body N2 shape) or (body N2 shapes) or (body N1 size)) OR TI(body N10 weight) OR AB(body N10 weight) OR TI("underweight" not "birth weight") OR AB("underweight" not "birth weight") OR TI((weight N1 bias) or (weight N1 biases)) OR AB((weight N1 bias) or (weight N1 biases)) OR TI("weight change" or "weight changes" or (weight N2 gain*) or (weight N2 loss) or "lose weight" or "losing weight" or (weight N1 (reduc* or manage* or control*))) OR AB("weight change" or "weight changes" or (weight N2 gain*) or (weight N2 loss) or "lose weight" or "losing weight" or (weight N1 (reduc* or manage* or control*))) OR TI("weight N1 measurement*" or "height N1 measurement*") OR AB("weight N1 measurement*" or "height N1 measurement*") OR TI("healthy weight" or "unhealthy weight" or "weight N5 monitor*" or "height N5 monitor*") OR AB("healthy weight" or "unhealthy weight" or "weight N5 monitor*" or "height N5 monitor*") OR TI("being measured" and ("weight" or weighing or height)) OR AB("healthy weight" or "unhealthy weight" or "weight N5 monitor*" or "height N5 monitor*") OR TI(weighing N3 experiences) OR AB(weighing N3 experiences) OR TI(weight screening* or height screening*) OR AB(weight screening* or height screening*) OR TI("being fat" or "being weighed" or "Being thin" or "Being tall" or "Being short") OR AB("being fat" or "being weighed" or "Being thin" or "Being tall" or "Being short") OR TI((stereotype or stereotypes or Stigma or stigmati* or ideals or ideal or discriminate* or discrimination* or "peer pressure" or prejudice* or bully* or teasing or cyberbull* or bullied) N5 ("fat" or weight or thin or height or muscle* or muscular* or body)) OR AB((stereotype or stereotypes or Stigma or stigmati* or ideals or ideal or discriminate* or discrimination* or "peer pressure" or prejudice* or bully* or teasing or cyberbull* or bullied) N5 ("fat" or weight or thin or height or muscle* or muscular* or body)) OR TI((fat or weight or thin or body or height or muscle or muscular*) N5 (acceptability or satisfaction or satisfied or dissatisfaction or dissatisfied or anxiety or anxious or angst or peer pressure or feelings or worries or worry or concern or concerns or "over concern" or emotions or emotion or concerns or "self-worth" or "self esteem" or esteem or "self-concept" or "self-identification" or "pre-occupation" or "preoccupation" or "acceptability" or perceiv* or understand* or perception* or internalis* or attitud*)) OR AB((fat or weight or thin or body or height or muscle or muscular*) N5 (acceptability or satisfaction or satisfied or dissatisfaction or dissatisfied or anxiety or anxious or angst or peer pressure or feelings or worries or worry or concern or concerns or "over concern" or emotions or emotion or concerns or "self-worth" or "self esteem" or esteem or "self-concept" or "self-identification" or "pre-occupation" or "preoccupation" or "acceptability" or perceiv* or understand* or perception* or internalis* or attitud*)) OR TI(Body N1 (dysmorph* or dismorph*)) OR AB(Body N1 (dysmorph* or dismorph*)) OR TI((body or fat or thin or height or weight) N3 (shame or phobia*)) OR AB((body or fat or thin or height or weight) N3 (shame or phobia*)) OR TI((weight N3 appearance) or (height N3 appearance)) OR AB((weight N3 appearance) or (height N3 appearance)) Expanders - Apply equivalent subjects Search modes - Proximity Interface - EBSCOhost Research Databases Search Screen - Advanced Search Database - ERIC 8,978 |
| Items coded | 543 |
| Uploaded documents | 7 |
| Masters of duplicates | 74 |
| Deleted items | 280 |
| Outcomes | 0 |
| Import filter | RIS |
| Is deleted? | false |

| Source name | **BEI** |
| --- | --- |
| Database name/platform | **BEI (EBSCO)** |
| Date of search | Jan 31, 2025 |
| Date of import | Jan 31, 2025 |
| Number items | 211 |
| Duplicates | 162 |
| Description |  |
| Notes |  |
| Search string | S10 S9 Limiters - Publication Date: 20080101-20251231 Expanders - Apply equivalent subjects Search modes - Proximity Interface - EBSCOhost Research Databases Search Screen - Advanced Search Database - British Education Index 211 S9 (S2 OR S4) AND S7 AND S8 Expanders - Apply equivalent subjects Search modes - Proximity Interface - EBSCOhost Research Databases Search Screen - Advanced Search Database - British Education Index 224 S8 S3 OR S6 Expanders - Apply equivalent subjects Search modes - Proximity Interface - EBSCOhost Research Databases Search Screen - Advanced Search Database - British Education Index 57,523 S7 S1 OR S5 Expanders - Apply equivalent subjects Search modes - Proximity Interface - EBSCOhost Research Databases Search Screen - Advanced Search Database - British Education Index 1,356 S6 DE "QUALITATIVE research" OR DE "FOCUS groups" OR DE "PHENOMENOGRAPHY" Expanders - Apply equivalent subjects Search modes - Proximity Interface - EBSCOhost Research Databases Search Screen - Advanced Search Database - British Education Index 5,647 S5 DE ("BODY COMPOSITION") OR (SU "Obesity") OR (SU "Attitude to Obesity") OR (SU "Weight Bias") OR (SU "Pediatric Obesity") OR (SU "Weight Gain") OR (SU "Body Weight") OR (SU "Body Weight Changes") OR (SU "Weight Loss") OR (SU "Thinness") OR (SU "Body Dysmorphic Disorder") OR (SU "Body Height") OR (SU "Body Dissatisfaction") OR (SU "Body Size") OR (SU "Body Image") OR (SU "Body Satisfaction") Expanders - Apply equivalent subjects Search modes - Proximity Interface - EBSCOhost Research Databases Search Screen - Advanced Search Database - British Education Index 733 S4 DE "BOYS" OR DE "SCHOOL children" OR DE "ADOLESCENCE" OR DE "TEENAGERS" OR DE "CHILDREN" OR DE "YOUTH" Expanders - Apply equivalent subjects Search modes - Proximity Interface - EBSCOhost Research Databases Search Screen - Advanced Search Database - British Education Index 33,962 S3 ( (TI("experiences" or "lived experience*" or "perceptions" or "perspective" or "perspectives" or "experience" or "attitude" or "attitudes" or "attitudinal" or "belief" or "beliefs" or "discourse" or "discourses" or "life-world" or "opinions" or "perceived" or "perception" or "standpoint" or "standpoints" or "understanding" or "understandings" or "viewpoint" or "viewpoints" or "views" or "voice" or "voices" or talk*) AND TI("young people*" or adolescent* or youth or pubertal or pubescent or "pre adolescent" or "Pre pubescent" or "pre pubertal" or teen* or preteen* OR tweens or tweenage* or youth or youths or schoolboy* or schoolgirl* or "school aged" or "young person*" or juvenile* or "Boy" or "boys" or "child" or "children*" or "child's" or "Girl" or "girls" or "Minors" or "preadolescent" or "Prepubescent" or "schoolchild*" or pubescent or "early adolescent*")) OR ( AB((Girls or Boys or Child* or teen* or adolescen* or "young people*" or schoolchild* or pupil* or student*) N2 ("perspective*" or "experience*" or "lived experience*" or "attitude" or "attitudes" or "attitudinal" or "belief" or "beliefs" or "discourse" or "discourses" or "life-world" or "opinions" or "standpoint" or "standpoints" or "understanding" or "understandings" or "viewpoint" or "viewpoints" or "views" or "voice" or "voices" or discussed or described* or drawings or drew)) ) OR ( AB((Girls or Boys or Child* or teen* or adolescen* or "young people*" or schoolchild* or pupil* or student*) N2 ("perspective*" or "experience*" or "lived experience*" or "attitude" or "attitudes" or "attitudinal" or "belief" or "beliefs" or "discourse" or "discourses" or "life-world" or "opinions" or "standpoint" or "standpoints" or "understanding" or "understandings" or "viewpoint" or "viewpoints" or "views" or "voice" or "voices" or discussed or described* or drawings or drew)) TI("audiorecording" or "ethnograph*" or "ethnolog*" or "ethno psycholog*" or "ethnopsycholog*" or "Focus Groups" or "Focus Group" or "repertory grid" or "stories" or "audio record" or "audio recorded" or "audio recorder" or "audio recording" or "audio recordings" or "audio records" or "thematic analysis" or "phenomenol*" or "grounded theory" or "grounded studies" or "grounded research" or "purposive sampling" or "biographical method" or "theoretical sampl*" or "conversation analysis" or "theoretical saturation" or "mixed design" or "mixed method" or "mixed methods" or "qualitative" or "interviewed" or "interviewing" or "interviewer" or "narratives" or "Diary entries" or "diary study" or Diaries or journalling or (Question* N5 (semi-structured or semistructured or unstructured or informal or in-depth or indepth or open or "open ended")) or "Group discussion*" or "interviews" or "interview") OR AB("audiorecording" or "ethnograph*" or "ethnolog*" or "ethno psycholog*" or "ethnopsycholog*" or "Focus Groups" or "Focus Group" or "repertory grid" or "stories" or "audio record" or "audio recorded" or "audio recorder" or "audio recording" or "audio recordings" or "audio records" or "thematic analysis" or "phenomenol*" or "grounded theory" or "grounded studies" or "grounded research" or "purposive sampling" or "biographical method" or "theoretical sampl*" or "conversation analysis" or "theoretical saturation" or "mixed design" or "mixed method" or "mixed methods" or "qualitative" or "interviewed" or "interviewing" or "interviewer" or "narratives" or "Diary entries" or "diary study" or Diaries or journalling or (Question* N5 (semi-structured or semistructured or unstructured or informal or in-depth or indepth or open or "open ended")) or "Group discussion*" or "interviews" or "interview") ) Expanders - Apply equivalent subjects Search modes - Proximity Interface - EBSCOhost Research Databases Search Screen - Advanced Search Database - British Education Index 56,377 S2 TI("young people*" or adolescent* or youth or pubertal or pubescent or "pre adolescent" or "Pre pubescent" or "pre pubertal" or teen* or preteen* or tweens or tweenage* or youth or youths or schoolboy* or schoolgirl* or "school aged" or "young person*" or juvenile* or "Boy" or "boys" or "child" or "children*" or "child's" or "Girl" or "girls" or "Minors" or "preadolescent" or "Prepubescent" or "schoolchild*" or pubescent or "early adolescent*") OR AB("young people*" or adolescent* or youth or pubertal or pubescent or "pre adolescent" or "Pre pubescent" or "pre pubertal" or teen* or preteen* or tweens or tweenage* or youth or youths or schoolboy* or schoolgirl* or "school aged" or "young person*" or juvenile* or "Boy" or "boys" or "child" or "children*" or "child's" or "Girl" or "girls" or "Minors" or "preadolescent" or "Prepubescent" or "schoolchild*" or pubescent or "early adolescent*") Expanders - Apply equivalent subjects Search modes - Proximity Interface - EBSCOhost Research Databases Search Screen - Advanced Search Database - British Education Index 55,785 S1 TI(Weight not "birth weight") OR TI("anti-fat" or bodyweight or "obese" or "obesity" or "overweight" or "skinny" or "fatness" or "thinness" or "body image" or "body hatred" or "body positive" or "thin ideal*" or "fat ideal*") OR AB("anti-fat" or bodyweight or "obese" or "obesity" or "overweight" or "skinny" or "fatness" or "thinness" or "body image" or "body hatred" or "body positive" or "thin ideal*" or "fat ideal*") OR TI("body esteem" and ("weight" or height or fat or thin)) OR AB("body esteem" and ("weight" or height or fat or thin)) OR TI((body N2 ideal*) or (body N2 shape) or (body N2 shapes) or (body N1 size)) OR AB((body N2 ideal*) or (body N2 shape) or (body N2 shapes) or (body N1 size)) OR TI(body N10 weight) OR AB(body N10 weight) OR TI("underweight" not "birth weight") OR AB("underweight" not "birth weight") OR TI((weight N1 bias) or (weight N1 biases)) OR AB((weight N1 bias) or (weight N1 biases)) OR TI("weight change" or "weight changes" or (weight N2 gain*) or (weight N2 loss) or "lose weight" or "losing weight" or (weight N1 (reduc* or manage* or control*))) OR AB("weight change" or "weight changes" or (weight N2 gain*) or (weight N2 loss) or "lose weight" or "losing weight" or (weight N1 (reduc* or manage* or control*))) OR TI("weight N1 measurement*" or "height N1 measurement*") OR AB("weight N1 measurement*" or "height N1 measurement*") OR TI("healthy weight" or "unhealthy weight" or "weight N5 monitor*" or "height N5 monitor*") OR AB("healthy weight" or "unhealthy weight" or "weight N5 monitor*" or "height N5 monitor*") OR TI("being measured" and ("weight" or weighing or height)) OR AB("healthy weight" or "unhealthy weight" or "weight N5 monitor*" or "height N5 monitor*") OR TI(weighing N3 experiences) OR AB(weighing N3 experiences) OR TI(weight screening* or height screening*) OR AB(weight screening* or height screening*) OR TI("being fat" or "being weighed" or "Being thin" or "Being tall" or "Being short") OR AB("being fat" or "being weighed" or "Being thin" or "Being tall" or "Being short") OR TI((stereotype or stereotypes or Stigma or stigmati* or ideals or ideal or discriminate* or discrimination* or "peer pressure" or prejudice* or bully* or teasing or cyberbull* or bullied) N5 ("fat" or weight or thin or height or muscle* or muscular* or body)) OR AB((stereotype or stereotypes or Stigma or stigmati* or ideals or ideal or discriminate* or discrimination* or "peer pressure" or prejudice* or bully* or teasing or cyberbull* or bullied) N5 ("fat" or weight or thin or height or muscle* or muscular* or body)) OR TI((fat or weight or thin or body or height or muscle or muscular*) N5 (acceptability or satisfaction or satisfied or dissatisfaction or dissatisfied or anxiety or anxious or angst or peer pressure or feelings or worries or worry or concern or concerns or "over concern" or emotions or emotion or concerns or "self-worth" or "self esteem" or esteem or "self-concept" or "self-identification" or "pre-occupation" or "preoccupation" or "acceptability" or perceiv* or understand* or perception* or internalis* or attitud*)) OR AB((fat or weight or thin or body or height or muscle or muscular*) N5 (acceptability or satisfaction or satisfied or dissatisfaction or dissatisfied or anxiety or anxious or angst or peer pressure or feelings or worries or worry or concern or concerns or "over concern" or emotions or emotion or concerns or "self-worth" or "self esteem" or esteem or "self-concept" or "self-identification" or "pre-occupation" or "preoccupation" or "acceptability" or perceiv* or understand* or perception* or internalis* or attitud*)) OR TI(Body N1 (dysmorph* or dismorph*)) OR AB(Body N1 (dysmorph* or dismorph*)) OR TI((body or fat or thin or height or weight) N3 (shame or phobia*)) OR AB((body or fat or thin or height or weight) N3 (shame or phobia*)) OR TI((weight N3 appearance) or (height N3 appearance)) OR AB((weight N3 appearance) or (height N3 appearance)) Expanders - Apply equivalent subjects Search modes - Proximity Interface - EBSCOhost Research Databases Search Screen - Advanced Search Database - ERIC 8,978 |
| Items coded | 49 |
| Uploaded documents | 1 |
| Masters of duplicates | 2 |
| Deleted items | 162 |
| Outcomes | 0 |
| Import filter | RIS |
| Is deleted? | false |

| Source name | **WalesThesis library** |
| --- | --- |
| Database name/platform |  |
| Date of search | Feb 19, 2025 |
| Date of import | Feb 19, 2025 |
| Number items | 6 |
| Duplicates | 1 |
| Description |  |
| Notes |  |
| Search string | Saved 10 items after browsing titles from the results  Year limit 2008-  Search Filters Any field contains obesity OR Any field contains body size OR Any field contains body weight OR Any field contains obese OR Any field contains body image OR Any field contains ideal body OR Title contains thin OR Title contains fat   Search Filters Any field contains fatness OR Any field contains thinness OR Any field contains "body positive" OR Any field contains "body weight" OR Any field contains "being fat" OR Any field contains "being thin" OR Any field contains overweight OR Any field contains weight OR Any field contains body |
| Items coded | 5 |
| Uploaded documents | 1 |
| Masters of duplicates | 1 |
| Deleted items | 1 |
| Outcomes | 0 |
| Import filter | RIS |
| Is deleted? | false |

| Source name | **OATD** |
| --- | --- |
| Database name/platform | **OATD.org** |
| Date of search | Feb 19, 2025 |
| Date of import | Feb 19, 2025 |
| Number items | 21 |
| Duplicates | 0 |
| Description |  |
| Notes |  |
| Search string | 31 records saved from scanning 8 searches - results added in two batches  Advanced search does not seem to work, have tried this on several occasions. So ran 9 basic searches, refined by UK and scanned records by title  Search 1 - obesity views children Search 2 - weight views children Search 3 - body qualitative children - Search 4 obesity views adolescents Search 5 - weight views adolescents Search 6 - body qualitative adolescents Search 7 obesity experiences children Search 8 body experiences children Search 9 weight experiences children |
| Items coded | 21 |
| Uploaded documents | 5 |
| Masters of duplicates | 16 |
| Deleted items | 0 |
| Outcomes | 0 |
| Import filter | RIS |
| Is deleted? | false |

| Source name | **ProQuest-Thesis** |
| --- | --- |
| Database name/platform | **ProQuest Dissertations & Theses Global (ProQuest)** |
| Date of search | Feb 4, 2025 |
| Date of import | Feb 19, 2025 |
| Number items | 384 |
| Duplicates | 21 |
| Description |  |
| Notes |  |
| Search string | S1 TI(Weight NOT "birth weight") OR TI,AB("anti-fat" or bodyweight or "obese" or "obesity" or "overweight" or "skinny" or "fatness" or "thinness" or "body image" or "body hatred" or "body positive" or "thin ideal*" or "fat ideal*") OR TI,AB("body esteem" and ("weight" or height or fat or thin)) OR TI,AB(body NEAR/2 ideal*) or TI,AB(body NEAR/2 shape) or TI,AB(body NEAR/2 shapes) or TI,AB(body NEAR/1 size) OR TI,AB("underweight" NOT "birth weight") OR TI,AB(weight NEAR/1 bias) or TI,AB(weight NEAR/1 biases) OR TI,AB("weight change" or "weight changes") or TI,AB(weight NEAR/2 gain*) or TI,AB(weight NEAR/2 loss) or TI,AB("lose weight" or "losing weight") or TI,AB(weight NEAR/1 (reduc* or manage* or control*)) OR TI,AB("weight NEAR/1 measurement*" or "height NEAR/1 measurement*") OR TI,AB("healthy weight" or "unhealthy weight") or TI,AB("weight NEAR/5 monitor*" or "height NEAR/5 monitor*") OR TI,AB("being measured" and ("weight" or weighing or height)) OR TI,AB(weighing NEAR/3 experiences) OR TI,AB(weight screening* or height screening*) OR TI,AB("being fat" or "being weighed" or "Being thin" or "Being tall" or "Being short") OR TI,AB((stereotype or stereotypes or Stigma or stigmati* or ideals or ideal or discriminate* or discrimination* or prejudice* or bully* or teasing or cyberbull* or bullied) NEAR/5 ("fat" or weight or thin or height or muscle* or muscular* or body)) OR TI,AB((fat or weight or thin or body or height or muscle or muscular*) NEAR/5 (acceptability or satisfaction or satisfied or dissatisfaction or dissatisfied or anxiety or anxious or angst or "peer pressure" or feelings or worries or worry or concern or concerns or "over concern" or emotions or emotion)) or TI,AB((fat or weight or thin or body or height or muscle or muscular*) NEAR/5 (concerns or "self-worth" or "self esteem" or esteem or "self-concept" or "self-identification" or "pre-occupation" or "preoccupation" or "acceptability" or perceiv* or understand* or perception* or internalis* or attitud*)) OR TI,AB(Body NEAR/1 (dysmorph* or dismorph*)) OR TI,AB((body or fat or thin or height or weight) NEAR/3 (shame or phobia*)) OR TI,AB(weight NEAR/3 appearance) or TI,AB(height NEAR/3 appearance) ProQuest Dissertations & Theses Global 83710 S2 TI("experiences" or ("lived experience" OR "lived experiences") or "perceptions" or "perspective" or "perspectives" or "experience" or "attitude" or "attitudes" or "attitudinal" or "belief" or "beliefs" or "discourse" or "discourses" or "life-world" or "opinions" or "perceived" or "perception" or "standpoint" or "standpoints" or "understanding" or "understandings" or "viewpoint" or "viewpoints" or "views" or "voice" or "voices" or talk*) AND TI(("young people" OR "young peoples") or adolescent* or youth or pubertal or pubescent or "pre adolescent" or "Pre pubescent" or "pre pubertal" or teen* or preteen* OR tweens or tweenage* or youth or youths or schoolboy* or schoolgirl* or "school aged" or ("young person" OR "young persons") or juvenile* or "Boy" or "boys" or "child" or "children*" or "child's" or "Girl" or "girls" or "Minors" or "preadolescent" or "Prepubescent" or "schoolchild*" or pubescent or ("early adolescent" OR "early adolescents")) ProQuest Dissertations & Theses Global 29105 S3 [S1] AND [S2] ProQuest Dissertations & Theses Global These databases are searched for part of your query. 825 S4 ([S1] AND [S2]) AND pd(20080101-20251231) ProQuest Dissertations & Theses Global These databases are searched for part of your query. 526 S5 ([S1] AND [S2]) AND pd(20080101-20251231) ProQuest Dissertations & Theses Global These databases are searched for part of your query. 526 S6 TI,AB("audiorecording" or "ethnograph*" or "ethnolog*" or "ethno psycholog*" or "ethnopsycholog*" or "Focus Groups" or "Focus Group" or "repertory grid" or "stories" or "audio record" or "audio recorded" or "audio recorder" or "audio recording" or "audio recordings" or "audio records" or "thematic analysis" or "phenomenol*" or "grounded theory" or "grounded studies" or "grounded research" or "purposive sampling" or "biographical method" or "theoretical sampl*" or "conversation analysis" or "theoretical saturation" or "mixed design" or "mixed method" or "mixed methods" or "qualitative" or "interviewed" or "interviewing" or "interviewer" or "narratives" or "Diary entries" or "diary study" or Diaries or journalling or (Question* NEAR/5 ("semi structured" or semistructured or unstructured or informal or in-depth or indepth or open or "open ended")) or "Group discussion*" or ("interviews" or "interview" NOT ("structured interview*")) OR "semi structured interview*") ProQuest Dissertations & Theses Global 699119 S7 TI,AB("young people*" or adolescent* or youth or pubertal or pubescent or "pre adolescent" or "Pre pubescent" or "pre pubertal" or teen* or preteen* OR tweens or tweenage* or youth or youths or schoolboy* or schoolgirl* or "school aged" or "young person*" or juvenile* or "Boy" or "boys" or "child" or "children*" or "child's" or "Girl" or "girls" or "Minors" or "preadolescent" or "Prepubescent" or "schoolchild*" or pubescent or "early adolescent*") ProQuest Dissertations & Theses Global 417984 S8 [S1] AND [S6] AND [S7] ProQuest Dissertations & Theses Global These databases are searched for part of your query. 2734 S9 AB((Girls or Boys or Child* or teen* or adolescen* or "young people*" or schoolchild* or pupil* or student*) NEAR/2 ("perspective*" or "experience*" or "lived experience*" or "attitude" or "attitudes" or "attitudinal" or "belief" or "beliefs" or "discourse" or "discourses" or "life-world" or "opinions" or "standpoint" or "standpoints" or "understanding" or "understandings" or "viewpoint" or "viewpoints" or "views" or "voice" or "voices" or discussed or described* or drawings or drew)) ProQuest Dissertations & Theses Global 104832 S10 [S1] AND [S9] ProQuest Dissertations & Theses Global These databases are searched for part of your query. 1660 S11 [S5] OR [S8] OR [S10] ProQuest Dissertations & Theses Global These databases are searched for part of your query. 4088 S12 ([S5] OR [S8] OR [S10]) AND pd(20080101-20251231) ProQuest Dissertations & Theses Global These databases are searched for part of your query. 3008 S13 ([S5] OR [S8] OR [S10]) AND pd(20080101-20251231) ProQuest Dissertations & Theses Global These databases are searched for part of your query. 3008 S14 ([S5] OR [S8] OR [S10]) NOT (schloc.exact("United States -- California" OR "Portugal" OR "United States -- Illinois" OR "United States -- Minnesota" OR "United States -- New York" OR "United States -- Texas" OR "Canada -- Ontario, CA" OR "United States -- North Carolina" OR "United States -- Pennsylvania" OR "United States -- Massachusetts" OR "United States -- Florida" OR "Turkey" OR "India" OR "United States -- Ohio" OR "Peoples Rep. of China" OR "United States -- Michigan" OR "United States -- Arizona" OR "Israel" OR "Sweden" OR "United States -- Colorado" OR "United States -- Washington" OR "United States -- Connecticut" OR "United States -- Virginia" OR "South Africa" OR "United States -- South Carolina" OR "Lithuania" OR "United States -- Indiana" OR "United States -- Tennessee" OR "United States -- Maryland" OR "United States -- Wisconsin" OR "Canada -- Quebec, CA" OR "United States -- Utah" OR "United States -- Iowa" OR "United States -- Mississippi" OR "United States -- Oklahoma" OR "United States -- Alabama" OR "United States -- Georgia" OR "United States -- Missouri" OR "United States -- New Jersey" OR "United States -- Nebraska" OR "Canada -- Alberta, CA" OR "United States -- Kansas" OR "United States -- West Virginia" OR "Ireland" OR "United States -- District of Columbia" OR "United States -- North Dakota" OR "United States -- Louisiana" OR "United States -- Rhode Island" OR "Netherlands" OR "United States -- Nevada" OR "United States -- Kentucky" OR "United States -- Delaware" OR "United States -- Hawaii" OR "United States -- New Mexico" OR "Australia" OR "Taiwan R.O.C." OR "United States -- Arkansas" OR "Canada -- Saskatchewan, CA" OR "United States -- New Hampshire" OR "Hong Kong" OR "Hungary" OR "Canada -- British Columbia, CA" OR "Colombia" OR "United States -- California, US" OR "United States -- Idaho" OR "United States -- Maine" OR "United States -- South Dakota" OR "Canada -- Manitoba, CA" OR "Germany" OR "United States -- Oregon" OR "United States -- Puerto Rico" OR "United States -- Vermont" OR "United States -- Wyoming" OR "Brazil" OR "Canada -- New Brunswick, CA" OR "Canada -- Nova Scotia, CA" OR "Chile" OR "Malaysia" OR "Canada -- Newfoundland and Labrador, CA" OR "Mexico" OR "Serbia" OR "Slovenia" OR "Spain" OR "United States -- New York, US" OR "Egypt" OR "France" OR "Greece" OR "Nigeria" OR "Qatar" OR "Republic of Singapore" OR "Saudi Arabia" OR "Turks and Caicos Islands" OR "United States -- Alaska" OR "United States -- Arizona, US" OR "United States -- Massachusetts, US") AND pd(20080101-20251231)) ProQuest Dissertations & Theses Global These databases are searched for part of your query. 384 |
| Items coded | 363 |
| Uploaded documents | 30 |
| Masters of duplicates | 77 |
| Deleted items | 21 |
| Outcomes | 0 |
| Import filter | RIS |
| Is deleted? | false |

| Source name | **OpenDissertations** |
| --- | --- |
| Database name/platform | **EBSCO** |
| Date of search | Feb 17, 2025 |
| Date of import | Feb 19, 2025 |
| Number items | 111 |
| Duplicates | 48 |
| Description |  |
| Notes |  |
| Search string | S6 S4 Expanders - Apply equivalent subjects Narrow by AuthorUniversity: - University Of East Anglia Narrow by AuthorUniversity: - University Of Bath Narrow by AuthorUniversity: - Loughborough University Narrow by AuthorUniversity: - Liverpool John Moores University Narrow by AuthorUniversity: - University Of Sheffield Narrow by AuthorUniversity: - University Of Oxford Narrow by AuthorUniversity: - University Of Manchester Narrow by AuthorUniversity: - University Of Bristol Narrow by AuthorUniversity: - City, University Of London Narrow by AuthorUniversity: - University Of Newcastle Upon Tyne Narrow by AuthorUniversity: - University Of Exeter Narrow by AuthorUniversity: - University Of Glasgow Narrow by AuthorUniversity: - King's College London (University Of London) Narrow by AuthorUniversity: - Durham University Narrow by AuthorUniversity: - Cardiff University Narrow by AuthorUniversity: - University Of Warwick Narrow by AuthorUniversity: - University Of Leeds Narrow by AuthorUniversity: - University Of Edinburgh Narrow by AuthorUniversity: - University Of Birmingham Narrow by AuthorUniversity: - University College London (University Of London) Search modes - Proximity Interface - EBSCOhost Research Databases Search Screen - Advanced Search Database - OpenDissertations 111 S5 S4 Expanders - Apply equivalent subjects Search modes - Proximity Interface - EBSCOhost Research Databases Search Screen - Advanced Search Database - OpenDissertations 452 S4 S2 AND S3 Expanders - Apply equivalent subjects Search modes - Proximity Interface - EBSCOhost Research Databases Search Screen - Advanced Search Database - OpenDissertations 452 S3 qualitative OR "focus group" OR "focus groups" OR questionnaire OR questionnaires OR interview OR interviews OR "experiences" OR "lived experience*" OR "perceptions" OR "perspective" OR "perspectives" OR "attitude" OR "attitudes" OR "beliefs" OR diary OR diaries Limiters - Publication Date: 20080101-20241231 Expanders - Apply equivalent subjects Search modes - Proximity Interface - EBSCOhost Research Databases Search Screen - Advanced Search Database - OpenDissertations 195,180 S2 (schoolchildren OR teens OR teenage* OR children* OR adolescent* OR "young people*" OR girls OR boys OR youth ) AND TI ( weight OR obese OR fat OR thin OR fatness OR thinness OR overweight OR body OR obesity ) Limiters - Publication Date: 20080101-20241231 Expanders - Apply equivalent subjects Search modes - Proximity Interface - EBSCOhost Research Databases Search Screen - Advanced Search Database - OpenDissertations 1,125 S1 ( children OR adolescent OR young OR girls OR boys OR youth ) AND TI ( weight OR obese OR fat OR thin OR fatness OR thinness OR overweight OR body OR obesity ) Expanders - Apply equivalent subjects Search modes - Proximity Interface - EBSCOhost Research Databases Search Screen - Advanced Search Database - OpenDissertations 2,072 |
| Items coded | 63 |
| Uploaded documents | 1 |
| Masters of duplicates | 14 |
| Deleted items | 48 |
| Outcomes | 0 |
| Import filter | RIS |
| Is deleted? | false |

| Source name | **BASE** |
| --- | --- |
| Database name/platform | **Bielefeld Academic Search Engine (BASE)** |
| Date of search | Feb 17, 2025 |
| Date of import | Feb 19, 2025 |
| Number items | 12 |
| Duplicates | 0 |
| Description |  |
| Notes |  |
| Search string | (focussed search for reports or theses)  Advanced search mode children OR adolescent OR young OR girls OR boys OR youth tit:weight OR tit:obese OR tit:fat OR tit:thin OR tit:fatness OR tit:thinness OR tit:overweight OR tit:body OR tit:obesity country:uk doctype:(11* 14 183) year:[2008 TO 2025]  Saved 32 items and imported into Endnote. filtered out non-English records/location and kept 12.  Tried other combinations but nothing seemed useful. |
| Items coded | 12 |
| Uploaded documents | 0 |
| Masters of duplicates | 0 |
| Deleted items | 0 |
| Outcomes | 0 |
| Import filter | RIS |
| Is deleted? | false |

| Source name | **SHEUandleads** |
| --- | --- |
| Database name/platform |  |
| Date of search | Feb 19, 2025 |
| Date of import | Feb 19, 2025 |
| Number items | 18 |
| Duplicates | 9 |
| Description |  |
| Notes |  |
| Search string | Searched the education and health journal archive obesity obese weight overweight body  searched research archive - https://sheu.org.uk/search/ obesity qualitative weight qualitative obese qualitative body qualitative  Saved 18 papers with browsing and following up leads to find the research the paper/link was referring to |
| Items coded | 9 |
| Uploaded documents | 2 |
| Masters of duplicates | 3 |
| Deleted items | 9 |
| Outcomes | 0 |
| Import filter | RIS |
| Is deleted? | false |

| Source name | **GOV-UK** |
| --- | --- |
| Database name/platform |  |
| Date of search | Feb 19, 2025 |
| Date of import | Feb 19, 2025 |
| Number items | 1 |
| Duplicates | 0 |
| Description |  |
| Notes |  |
| Search string | gov.uk 19/02/2025 filter: type: research and statistics; updated after 1 January 2008 search terms: overweight scanned 86 results by title height scanned 28 results by title weight scanned 248 results by title body image scanned 155 results by title body size scanned 158 results by title Saved 1 paper |
| Items coded | 1 |
| Uploaded documents | 0 |
| Masters of duplicates | 0 |
| Deleted items | 0 |
| Outcomes | 0 |
| Import filter | RIS |
| Is deleted? | false |

| Source name | **PolicyCommons** |
| --- | --- |
| Database name/platform |  |
| Date of search | Feb 19, 2025 |
| Date of import | Feb 19, 2025 |
| Number items | 15 |
| Duplicates | 0 |
| Description |  |
| Notes |  |
| Search string | Searches: Multiple searches were tried, results scanned by title and relevant items saved.  Limts: Country UK. Publications, 2008-2028, book, case study, conference paper, discussion/working paper or proceedings or report or thesis/dissertation  summary:'weight NEAR/5 experience' summary:'obesity NEAR/5 experience' summary:'fat NEAR/5 experience' summary:'body ideal NEAR/5 experience' summary:'obese NEAR/5 experience' summary:'fat NEAR/5 experience' summary:'overweight NEAR/5 experience' summary:'body size NEAR/5 experience' summary:'body size NEAR/5 views'  Saved 2 items from above  title:(weight OR obese OR fat OR thin OR fatness OR thinness OR overweight OR body shape OR body ideal OR body shame OR body size OR body shape OR body esteem OR obesity OR height OR body positive) AND title:(view OR attitude OR experience OR understanding OR opinion OR perception OR stigma OR beliefs OR qualitative OR discussion OR group OR interview OR questionnaire OR talk OR speak OR voice OR stereotype OR perspective OR diary OR listen) browsed 63 results saved 3/4 items  I also tried this search with the UK filter off and it looked like any relevant records were not UK based anyway.  summary:(weight OR obese OR fat OR thin OR fatness OR thinness OR overweight OR body shape OR body ideal OR body shame OR body size OR body shape OR body esteem OR obesity OR height OR body positive) AND summary:(view OR attitude OR experience OR understanding OR opinion OR perception OR stigma OR beliefs OR qualitative OR discussion OR group OR interview OR questionnaire OR talk OR speak OR voice OR stereotype OR perspective OR diary OR listen) 2,520 records  browsed first 200 results listed by relevance - 0 new  Search for (weight OR obese OR fat OR thin OR fatness OR thinness OR overweight) AND title:(children OR adolescen OR young people OR boys OR girls OR schoolchildren)  browsed first 300 results listed by relevance - 0 new   (body shape OR body ideal OR body shame OR body size OR body shape OR body esteem OR obesity OR height OR body positive) AND title:(children OR adolescen OR young people OR boys OR girls OR schoolchildren)  browsed first 300 results listed by relevance   (body shape OR body ideal OR body shame OR body size OR body shape OR body esteem OR obesity OR height OR body positive) AND title:(children OR adolescen OR young people OR boys OR girls OR schoolchildren) 4,261 results  Search for (overweight) AND title:(children OR adolescen OR young people OR boys OR girls OR schoolchildren)  browsed first 200 results listed by relevance - 0 new |
| Items coded | 15 |
| Uploaded documents | 0 |
| Masters of duplicates | 0 |
| Deleted items | 0 |
| Outcomes | 0 |
| Import filter | RIS |
| Is deleted? | false |

| Source name | **OpenAlex** |
| --- | --- |
| Database name/platform | **openalex.org** |
| Date of search | Feb 21, 2025 |
| Date of import | Mar 3, 2025 |
| Number items | 1941 |
| Duplicates | 412 |
| Description |  |
| Notes |  |
| Search string | OpenAlex search 21-2-2025 Deduplicated the results before uploading to EPPI-Reviewer.  Searches were intended to be a focused search across all publication types and a broader search to find grey literature.   2,412 records after running 12 searches and de-duplicating 1,941 after removing publication date before 2008   focused search 1: 726 records  https://openalex.org/works?filter=display_name.search:"obese"\|"overweight"\|"obesity"\|"body image"\|fat\|fatness\|thin\|thinness\|"Height"\|weight\|bodyweight\|"body hatred"\|"body positive"\|"thin ideal"\|"fat ideal"\|"body esteem"\|"body ideal"\|"ideal body"\|"body shape"\|"body size"\|"body sizes"\|"weight bias"\|"being fat"\|"being thin"\|"anti fat"\|"body shame",title_and_abstract.search:qualitative\|"focus group"\|"focus groups"\|questionnaire\|questionnaires\|interview\|interviews\|"experiences"\|"lived experience"\|"perceptions"\|"perspective"\|"perspectives"\|"attitude"\|"attitudes"\|"beliefs"\|diary\|diaries,title_and_abstract.search:qualitative\|"focus group"\|"focus groups"\|"semi structured"\|semistructured\|unstructured\|informal\|in-depth\|indepth\|open\|"open ended"\|"experiences"\|"lived experience"\|"perceptions"\|"perspective"\|"perspectives"\|"attitude"\|"attitudes"\|"beliefs"\|diary\|diaries,display_name.search:"young people"\|adolescent\|youth\|pubertal\|pubescent\|"pre adolescent"\|"Pre pubescent"\|"pre pubertal"\|teen\|preteen\|tweens\|tweenage\|youth\|youths\|schoolboy\|schoolgirl\|"school aged"\|"young person"\|juvenile\|"Boy"\|"boys"\|"children"\|"child's"\|"Girl"\|"girls"\|"Minors"\|"preadolescent"\|"Prepubescent"\|"schoolchild"\|pubescent\|"early adolescent",default.search:GB\|Britain\|UK\|"United Kingdom"\|England\|"Northern Ireland"\|"Northern Irish"\|"North Ireland"\|"North Irish"\|Scotland\|Scottish\|Wales\|Welsh\|"english boys"\|"English girls"\|"english school"\|"English children"\|"English teen"\|"English adolescent"\|"English young people"\|"English schoolchild"  search 2 - remove terms for country and apply country filter 404 records  https://openalex.org/works?filter=display_name.search%3A%22obese%22%7C%22overweight%22%7C%22obesity%22%7C%22body%20image%22%7Cfat%7Cfatness%7Cthin%7Cthinness%7C%22Height%22%7Cweight%7Cbodyweight%7C%22body%20hatred%22%7C%22body%20positive%22%7C%22thin%20ideal%22%7C%22fat%20ideal%22%7C%22body%20esteem%22%7C%22body%20ideal%22%7C%22ideal%20body%22%7C%22body%20shape%22%7C%22body%20size%22%7C%22body%20sizes%22%7C%22weight%20bias%22%7C%22being%20fat%22%7C%22being%20thin%22%7C%22anti%20fat%22%7C%22body%20shame%22,title_and_abstract.search%3Aqualitative%7C%22focus%20group%22%7C%22focus%20groups%22%7Cquestionnaire%7Cquestionnaires%7Cinterview%7Cinterviews%7C%22experiences%22%7C%22lived%20experience%22%7C%22perceptions%22%7C%22perspective%22%7C%22perspectives%22%7C%22attitude%22%7C%22attitudes%22%7C%22beliefs%22%7Cdiary%7Cdiaries,title_and_abstract.search%3Aqualitative%7C%22focus%20group%22%7C%22focus%20groups%22%7C%22semi%20structured%22%7Csemistructured%7Cunstructured%7Cinformal%7Cin-depth%7Cindepth%7Copen%7C%22open%20ended%22%7C%22experiences%22%7C%22lived%20experience%22%7C%22perceptions%22%7C%22perspective%22%7C%22perspectives%22%7C%22attitude%22%7C%22attitudes%22%7C%22beliefs%22%7Cdiary%7Cdiaries,display_name.search%3A%22young%20people%22%7Cadolescent%7Cyouth%7Cpubertal%7Cpubescent%7C%22pre%20adolescent%22%7C%22Pre%20pubescent%22%7C%22pre%20pubertal%22%7Cteen%7Cpreteen%7Ctweens%7Ctweenage%7Cyouth%7Cyouths%7Cschoolboy%7Cschoolgirl%7C%22school%20aged%22%7C%22young%20person%22%7Cjuvenile%7C%22Boy%22%7C%22boys%22%7C%22children%22%7C%22child%27s%22%7C%22Girl%22%7C%22girls%22%7C%22Minors%22%7C%22preadolescent%22%7C%22Prepubescent%22%7C%22schoolchild%22%7Cpubescent%7C%22early%20adolescent%22,authorships.countries%3Acountries%2Fgb&page=1  Search 3 - remove terms for obesity and search on obesity as a topic within health promotion domain - 833 records  https://openalex.org/works?filter=title_and_abstract.search%3Aqualitative%7C%22focus%20group%22%7C%22focus%20groups%22%7Cquestionnaire%7Cquestionnaires%7Cinterview%7Cinterviews%7C%22experiences%22%7C%22lived%20experience%22%7C%22perceptions%22%7C%22perspective%22%7C%22perspectives%22%7C%22attitude%22%7C%22attitudes%22%7C%22beliefs%22%7Cdiary%7Cdiaries,title_and_abstract.search%3Aqualitative%7C%22focus%20group%22%7C%22focus%20groups%22%7C%22semi%20structured%22%7Csemistructured%7Cunstructured%7Cinformal%7Cin-depth%7Cindepth%7Copen%7C%22open%20ended%22%7C%22experiences%22%7C%22lived%20experience%22%7C%22perceptions%22%7C%22perspective%22%7C%22perspectives%22%7C%22attitude%22%7C%22attitudes%22%7C%22beliefs%22%7Cdiary%7Cdiaries,display_name.search%3A%22young%20people%22%7Cadolescent%7Cyouth%7Cpubertal%7Cpubescent%7C%22pre%20adolescent%22%7C%22Pre%20pubescent%22%7C%22pre%20pubertal%22%7Cteen%7Cpreteen%7Ctweens%7Ctweenage%7Cyouth%7Cyouths%7Cschoolboy%7Cschoolgirl%7C%22school%20aged%22%7C%22young%20person%22%7Cjuvenile%7C%22Boy%22%7C%22boys%22%7C%22children%22%7C%22child%27s%22%7C%22Girl%22%7C%22girls%22%7C%22Minors%22%7C%22preadolescent%22%7C%22Prepubescent%22%7C%22schoolchild%22%7Cpubescent%7C%22early%20adolescent%22,default.search%3AGB%7CBritain%7CUK%7C%22United%20Kingdom%22%7CEngland%7C%22Northern%20Ireland%22%7C%22Northern%20Irish%22%7C%22North%20Ireland%22%7C%22North%20Irish%22%7CScotland%7CScottish%7CWales%7CWelsh%7C%22english%20boys%22%7C%22English%20girls%22%7C%22english%20school%22%7C%22English%20children%22%7C%22English%20teen%22%7C%22English%20adolescent%22%7C%22English%20young%20people%22%7C%22English%20schoolchild%22,primary_topic.id%3At10010&page=1  search 4 refine by type (type = book chapter, book, dissertation, report or other)  search for children/obesity concepts in title and abstract qualitative in full text AND UK country field 140 results  https://openalex.org/works?filter=title_and_abstract.search%3A%22obese%22%7C%22overweight%22%7C%22obesity%22%7C%22body%20image%22%7Cfat%7Cfatness%7Cthin%7Cthinness%7C%22Height%22%7Cweight%7Cbodyweight%7C%22body%20hatred%22%7C%22body%20positive%22%7C%22thin%20ideal%22%7C%22fat%20ideal%22%7C%22body%20esteem%22%7C%22body%20ideal%22%7C%22ideal%20body%22%7C%22body%20shape%22%7C%22body%20size%22%7C%22body%20sizes%22%7C%22weight%20bias%22%7C%22being%20fat%22%7C%22being%20thin%22%7C%22anti%20fat%22%7C%22body%20shame%22,default.search%3Aqualitative%7C%22focus%20group%22%7C%22focus%20groups%22%7Cquestionnaire%7Cquestionnaires%7Cinterview%7Cinterviews%7C%22experiences%22%7C%22lived%20experience%22%7C%22perceptions%22%7C%22perspective%22%7C%22perspectives%22%7C%22attitude%22%7C%22attitudes%22%7C%22beliefs%22%7Cdiary%7Cdiaries,default.search%3Aqualitative%7C%22focus%20group%22%7C%22focus%20groups%22%7C%22semi%20structured%22%7Csemistructured%7Cunstructured%7Cinformal%7Cin-depth%7Cindepth%7Copen%7C%22open%20ended%22%7C%22experiences%22%7C%22lived%20experience%22%7C%22perceptions%22%7C%22perspective%22%7C%22perspectives%22%7C%22attitude%22%7C%22attitudes%22%7C%22beliefs%22%7Cdiary%7Cdiaries,title_and_abstract.search%3A%22young%20people%22%7Cadolescent%7Cyouth%7Cpubertal%7Cpubescent%7C%22pre%20adolescent%22%7C%22Pre%20pubescent%22%7C%22pre%20pubertal%22%7Cteen%7Cpreteen%7Ctweens%7Ctweenage%7Cyouth%7Cyouths%7Cschoolboy%7Cschoolgirl%7C%22school%20aged%22%7C%22young%20person%22%7Cjuvenile%7C%22Boy%22%7C%22boys%22%7C%22children%22%7C%22child%27s%22%7C%22Girl%22%7C%22girls%22%7C%22Minors%22%7C%22preadolescent%22%7C%22Prepubescent%22%7C%22schoolchild%22%7Cpubescent%7C%22early%20adolescent%22,authorships.countries%3Acountries%2Fgb,type:types/book-chapter\|types/book\|types/dissertation\|types/report\|types/other   Search 5 repeated search 4 but replace country field with the full text search for country names and searched obesity in title only = 177 results   https://openalex.org/works?filter=display_name.search%3A%22obese%22%7C%22overweight%22%7C%22obesity%22%7C%22body%20image%22%7Cfat%7Cfatness%7Cthin%7Cthinness%7C%22Height%22%7Cweight%7Cbodyweight%7C%22body%20hatred%22%7C%22body%20positive%22%7C%22thin%20ideal%22%7C%22fat%20ideal%22%7C%22body%20esteem%22%7C%22body%20ideal%22%7C%22ideal%20body%22%7C%22body%20shape%22%7C%22body%20size%22%7C%22body%20sizes%22%7C%22weight%20bias%22%7C%22being%20fat%22%7C%22being%20thin%22%7C%22anti%20fat%22%7C%22body%20shame%22,default.search%3Aqualitative%7C%22focus%20group%22%7C%22focus%20groups%22%7Cquestionnaire%7Cquestionnaires%7Cinterview%7Cinterviews%7C%22experiences%22%7C%22lived%20experience%22%7C%22perceptions%22%7C%22perspective%22%7C%22perspectives%22%7C%22attitude%22%7C%22attitudes%22%7C%22beliefs%22%7Cdiary%7Cdiaries,default.search%3Aqualitative%7C%22focus%20group%22%7C%22focus%20groups%22%7C%22semi%20structured%22%7Csemistructured%7Cunstructured%7Cinformal%7Cin-depth%7Cindepth%7Copen%7C%22open%20ended%22%7C%22experiences%22%7C%22lived%20experience%22%7C%22perceptions%22%7C%22perspective%22%7C%22perspectives%22%7C%22attitude%22%7C%22attitudes%22%7C%22beliefs%22%7Cdiary%7Cdiaries,title_and_abstract.search%3A%22young%20people%22%7Cadolescent%7Cyouth%7Cpubertal%7Cpubescent%7C%22pre%20adolescent%22%7C%22Pre%20pubescent%22%7C%22pre%20pubertal%22%7Cteen%7Cpreteen%7Ctweens%7Ctweenage%7Cyouth%7Cyouths%7Cschoolboy%7Cschoolgirl%7C%22school%20aged%22%7C%22young%20person%22%7Cjuvenile%7C%22Boy%22%7C%22boys%22%7C%22children%22%7C%22child%27s%22%7C%22Girl%22%7C%22girls%22%7C%22Minors%22%7C%22preadolescent%22%7C%22Prepubescent%22%7C%22schoolchild%22%7Cpubescent%7C%22early%20adolescent%22,default.search%3AGB%7CBritain%7CUK%7C%22United%20Kingdom%22%7CEngland%7C%22Northern%20Ireland%22%7C%22Northern%20Irish%22%7C%22North%20Ireland%22%7C%22North%20Irish%22%7CScotland%7CScottish%7CWales%7CWelsh%7C%22english%20boys%22%7C%22English%20girls%22%7C%22english%20school%22%7C%22English%20children%22%7C%22English%20teen%22%7C%22English%20adolescent%22%7C%22English%20young%20people%22%7C%22English%20schoolchild%22,type%3Atypes%2Fbook-chapter%7Ctypes%2Fbook%7Ctypes%2Fdissertation%7Ctypes%2Freport%7Ctypes%2Fother  Search 6 as search 5 but replace obesity title search with obesity topic search: 186 records  https://openalex.org/works?filter=primary_topic.id%3At10010,default.search%3Aqualitative%7C%22focus%20group%22%7C%22focus%20groups%22%7Cquestionnaire%7Cquestionnaires%7Cinterview%7Cinterviews%7C%22experiences%22%7C%22lived%20experience%22%7C%22perceptions%22%7C%22perspective%22%7C%22perspectives%22%7C%22attitude%22%7C%22attitudes%22%7C%22beliefs%22%7Cdiary%7Cdiaries,default.search%3Aqualitative%7C%22focus%20group%22%7C%22focus%20groups%22%7C%22semi%20structured%22%7Csemistructured%7Cunstructured%7Cinformal%7Cin-depth%7Cindepth%7Copen%7C%22open%20ended%22%7C%22experiences%22%7C%22lived%20experience%22%7C%22perceptions%22%7C%22perspective%22%7C%22perspectives%22%7C%22attitude%22%7C%22attitudes%22%7C%22beliefs%22%7Cdiary%7Cdiaries,title_and_abstract.search%3A%22young%20people%22%7Cadolescent%7Cyouth%7Cpubertal%7Cpubescent%7C%22pre%20adolescent%22%7C%22Pre%20pubescent%22%7C%22pre%20pubertal%22%7Cteen%7Cpreteen%7Ctweens%7Ctweenage%7Cyouth%7Cyouths%7Cschoolboy%7Cschoolgirl%7C%22school%20aged%22%7C%22young%20person%22%7Cjuvenile%7C%22Boy%22%7C%22boys%22%7C%22children%22%7C%22child%27s%22%7C%22Girl%22%7C%22girls%22%7C%22Minors%22%7C%22preadolescent%22%7C%22Prepubescent%22%7C%22schoolchild%22%7Cpubescent%7C%22early%20adolescent%22,default.search:GB\|Britain\|UK\|"United Kingdom"\|England\|"Northern Ireland"\|"Northern Irish"\|"North Ireland"\|"North Irish"\|Scotland\|Scottish\|Wales\|Welsh\|"english boys"\|"English girls"\|"english school"\|"English children"\|"English teen"\|"English adolescent"\|"English young people"\|"English schoolchild",type:types/book-chapter\|types/book\|types/dissertation\|types/report\|types/other   search 7 - 48 records  https://openalex.org/works?filter=display_name.search:"fat"\|weight\|thin\|height\|muscle\|muscular\|body,display_name.search:stereotype\|Stigma\|stigmatise\|stigmatize\|discriminate\|discrimination\|prejudice\|bully\|teasing\|cyberbully\|dissatisfaction\|dissatisfied\|anxiety\|anxious\|angst\|"peer pressure"\|feelings\|worries\|worry\|concern\|concerns\|"over concern"\|emotions\|emotion\|dysmorphic\|dismorphic\|dysmorphia\|dismorphia,title_and_abstract.search:qualitative\|"focus group"\|"focus groups"\|questionnaire\|questionnaires\|interview\|interviews\|"experiences"\|"lived experience"\|"perceptions"\|"perspective"\|"perspectives"\|"attitude"\|"attitudes"\|"beliefs"\|diary\|diaries,title_and_abstract.search:qualitative\|"focus group"\|"focus groups"\|"semi structured"\|semistructured\|unstructured\|informal\|in-depth\|indepth\|open\|"open ended"\|"experiences"\|"lived experience"\|"perceptions"\|"perspective"\|"perspectives"\|"attitude"\|"attitudes"\|"beliefs"\|diary\|diaries,display_name.search:"young people"\|adolescent\|youth\|pubertal\|pubescent\|"pre adolescent"\|"Pre pubescent"\|"pre pubertal"\|teen\|preteen\|tweens\|tweenage\|youth\|youths\|schoolboy\|schoolgirl\|"school aged"\|"young person"\|juvenile\|"Boy"\|"boys"\|"children"\|"child's"\|"Girl"\|"girls"\|"Minors"\|"preadolescent"\|"Prepubescent"\|"schoolchild"\|pubescent\|"early adolescent",default.search:GB\|Britain\|UK\|"United Kingdom"\|England\|"Northern Ireland"\|"Northern Irish"\|"North Ireland"\|"North Irish"\|Scotland\|Scottish\|Wales\|Welsh\|"english boys"\|"English girls"\|"english school"\|"English children"\|"English teen"\|"English adolescent"\|"English young people"\|"English schoolchild"  search 8 - remove terms for country and apply country filter 30 records  https://openalex.org/works?filter=display_name.search:"fat"\|weight\|thin\|height\|muscle\|muscular\|body,display_name.search:stereotype\|Stigma\|stigmatise\|stigmatize\|discriminate\|discrimination\|prejudice\|bully\|teasing\|cyberbully\|dissatisfaction\|dissatisfied\|anxiety\|anxious\|angst\|"peer pressure"\|feelings\|worries\|worry\|concern\|concerns\|"over concern"\|emotions\|emotion\|dysmorphic\|dismorphic\|dysmorphia\|dismorphia,title_and_abstract.search%3Aqualitative%7C%22focus%20group%22%7C%22focus%20groups%22%7Cquestionnaire%7Cquestionnaires%7Cinterview%7Cinterviews%7C%22experiences%22%7C%22lived%20experience%22%7C%22perceptions%22%7C%22perspective%22%7C%22perspectives%22%7C%22attitude%22%7C%22attitudes%22%7C%22beliefs%22%7Cdiary%7Cdiaries,title_and_abstract.search%3Aqualitative%7C%22focus%20group%22%7C%22focus%20groups%22%7C%22semi%20structured%22%7Csemistructured%7Cunstructured%7Cinformal%7Cin-depth%7Cindepth%7Copen%7C%22open%20ended%22%7C%22experiences%22%7C%22lived%20experience%22%7C%22perceptions%22%7C%22perspective%22%7C%22perspectives%22%7C%22attitude%22%7C%22attitudes%22%7C%22beliefs%22%7Cdiary%7Cdiaries,display_name.search%3A%22young%20people%22%7Cadolescent%7Cyouth%7Cpubertal%7Cpubescent%7C%22pre%20adolescent%22%7C%22Pre%20pubescent%22%7C%22pre%20pubertal%22%7Cteen%7Cpreteen%7Ctweens%7Ctweenage%7Cyouth%7Cyouths%7Cschoolboy%7Cschoolgirl%7C%22school%20aged%22%7C%22young%20person%22%7Cjuvenile%7C%22Boy%22%7C%22boys%22%7C%22children%22%7C%22child%27s%22%7C%22Girl%22%7C%22girls%22%7C%22Minors%22%7C%22preadolescent%22%7C%22Prepubescent%22%7C%22schoolchild%22%7Cpubescent%7C%22early%20adolescent%22,authorships.countries%3Acountries%2Fgb&page=1  Search 9 ADD IN BODY IMAGE TOPIC - 24 records  https://openalex.org/works?filter=title_and_abstract.search%3Aqualitative%7C%22focus%20group%22%7C%22focus%20groups%22%7Cquestionnaire%7Cquestionnaires%7Cinterview%7Cinterviews%7C%22experiences%22%7C%22lived%20experience%22%7C%22perceptions%22%7C%22perspective%22%7C%22perspectives%22%7C%22attitude%22%7C%22attitudes%22%7C%22beliefs%22%7Cdiary%7Cdiaries,title_and_abstract.search%3Aqualitative%7C%22focus%20group%22%7C%22focus%20groups%22%7C%22semi%20structured%22%7Csemistructured%7Cunstructured%7Cinformal%7Cin-depth%7Cindepth%7Copen%7C%22open%20ended%22%7C%22experiences%22%7C%22lived%20experience%22%7C%22perceptions%22%7C%22perspective%22%7C%22perspectives%22%7C%22attitude%22%7C%22attitudes%22%7C%22beliefs%22%7Cdiary%7Cdiaries,display_name.search%3A%22young%20people%22%7Cadolescent%7Cyouth%7Cpubertal%7Cpubescent%7C%22pre%20adolescent%22%7C%22Pre%20pubescent%22%7C%22pre%20pubertal%22%7Cteen%7Cpreteen%7Ctweens%7Ctweenage%7Cyouth%7Cyouths%7Cschoolboy%7Cschoolgirl%7C%22school%20aged%22%7C%22young%20person%22%7Cjuvenile%7C%22Boy%22%7C%22boys%22%7C%22children%22%7C%22child%27s%22%7C%22Girl%22%7C%22girls%22%7C%22Minors%22%7C%22preadolescent%22%7C%22Prepubescent%22%7C%22schoolchild%22%7Cpubescent%7C%22early%20adolescent%22,default.search%3AGB%7CBritain%7CUK%7C%22United%20Kingdom%22%7CEngland%7C%22Northern%20Ireland%22%7C%22Northern%20Irish%22%7C%22North%20Ireland%22%7C%22North%20Irish%22%7CScotland%7CScottish%7CWales%7CWelsh%7C%22english%20boys%22%7C%22English%20girls%22%7C%22english%20school%22%7C%22English%20children%22%7C%22English%20teen%22%7C%22English%20adolescent%22%7C%22English%20young%20people%22%7C%22English%20schoolchild%22,primary_topic.id%3At12663&page=1  search 10 refine by type (type = book chapter, book, dissertation, report or other)  65 results  https://openalex.org/works?filter=title_and_abstract.search:"fat"\|weight\|thin\|height\|muscle\|muscular\|body,title_and_abstract.search:stereotype\|Stigma\|stigmatise\|stigmatize\|discriminate\|discrimination\|prejudice\|bully\|teasing\|cyberbully\|dissatisfaction\|dissatisfied\|anxiety\|anxious\|angst\|"peer pressure"\|feelings\|worries\|worry\|concern\|concerns\|"over concern"\|emotions\|emotion\|dysmorphic\|dismorphic\|dysmorphia\|dismorphia ,default.search%3Aqualitative%7C%22focus%20group%22%7C%22focus%20groups%22%7Cquestionnaire%7Cquestionnaires%7Cinterview%7Cinterviews%7C%22experiences%22%7C%22lived%20experience%22%7C%22perceptions%22%7C%22perspective%22%7C%22perspectives%22%7C%22attitude%22%7C%22attitudes%22%7C%22beliefs%22%7Cdiary%7Cdiaries,default.search%3Aqualitative%7C%22focus%20group%22%7C%22focus%20groups%22%7C%22semi%20structured%22%7Csemistructured%7Cunstructured%7Cinformal%7Cin-depth%7Cindepth%7Copen%7C%22open%20ended%22%7C%22experiences%22%7C%22lived%20experience%22%7C%22perceptions%22%7C%22perspective%22%7C%22perspectives%22%7C%22attitude%22%7C%22attitudes%22%7C%22beliefs%22%7Cdiary%7Cdiaries,title_and_abstract.search%3A%22young%20people%22%7Cadolescent%7Cyouth%7Cpubertal%7Cpubescent%7C%22pre%20adolescent%22%7C%22Pre%20pubescent%22%7C%22pre%20pubertal%22%7Cteen%7Cpreteen%7Ctweens%7Ctweenage%7Cyouth%7Cyouths%7Cschoolboy%7Cschoolgirl%7C%22school%20aged%22%7C%22young%20person%22%7Cjuvenile%7C%22Boy%22%7C%22boys%22%7C%22children%22%7C%22child%27s%22%7C%22Girl%22%7C%22girls%22%7C%22Minors%22%7C%22preadolescent%22%7C%22Prepubescent%22%7C%22schoolchild%22%7Cpubescent%7C%22early%20adolescent%22,authorships.countries%3Acountries%2Fgb,type:types/book-chapter\|types/book\|types/dissertation\|types/report\|types/other   Search 11 630 results   https://openalex.org/works?filter=title_and_abstract.search:"fat"\|weight\|thin\|height\|muscle\|muscular\|body,title_and_abstract.search:stereotype\|Stigma\|stigmatise\|stigmatize\|discriminate\|discrimination\|prejudice\|bully\|teasing\|cyberbully\|dissatisfaction\|dissatisfied\|anxiety\|anxious\|angst\|"peer pressure"\|feelings\|worries\|worry\|concern\|concerns\|"over concern"\|emotions\|emotion\|dysmorphic\|dismorphic\|dysmorphia\|dismorphia,default.search%3Aqualitative%7C%22focus%20group%22%7C%22focus%20groups%22%7Cquestionnaire%7Cquestionnaires%7Cinterview%7Cinterviews%7C%22experiences%22%7C%22lived%20experience%22%7C%22perceptions%22%7C%22perspective%22%7C%22perspectives%22%7C%22attitude%22%7C%22attitudes%22%7C%22beliefs%22%7Cdiary%7Cdiaries,default.search%3Aqualitative%7C%22focus%20group%22%7C%22focus%20groups%22%7C%22semi%20structured%22%7Csemistructured%7Cunstructured%7Cinformal%7Cin-depth%7Cindepth%7Copen%7C%22open%20ended%22%7C%22experiences%22%7C%22lived%20experience%22%7C%22perceptions%22%7C%22perspective%22%7C%22perspectives%22%7C%22attitude%22%7C%22attitudes%22%7C%22beliefs%22%7Cdiary%7Cdiaries,title_and_abstract.search%3A%22young%20people%22%7Cadolescent%7Cyouth%7Cpubertal%7Cpubescent%7C%22pre%20adolescent%22%7C%22Pre%20pubescent%22%7C%22pre%20pubertal%22%7Cteen%7Cpreteen%7Ctweens%7Ctweenage%7Cyouth%7Cyouths%7Cschoolboy%7Cschoolgirl%7C%22school%20aged%22%7C%22young%20person%22%7Cjuvenile%7C%22Boy%22%7C%22boys%22%7C%22children%22%7C%22child%27s%22%7C%22Girl%22%7C%22girls%22%7C%22Minors%22%7C%22preadolescent%22%7C%22Prepubescent%22%7C%22schoolchild%22%7Cpubescent%7C%22early%20adolescent%22,default.search%3AGB%7CBritain%7CUK%7C%22United%20Kingdom%22%7CEngland%7C%22Northern%20Ireland%22%7C%22Northern%20Irish%22%7C%22North%20Ireland%22%7C%22North%20Irish%22%7CScotland%7CScottish%7CWales%7CWelsh%7C%22english%20boys%22%7C%22English%20girls%22%7C%22english%20school%22%7C%22English%20children%22%7C%22English%20teen%22%7C%22English%20adolescent%22%7C%22English%20young%20people%22%7C%22English%20schoolchild%22,type%3Atypes%2Fbook-chapter%7Ctypes%2Fbook%7Ctypes%2Fdissertation%7Ctypes%2Freport%7Ctypes%2Fother  Search 12 5 results  https://openalex.org/works?filter=primary_topic.id%3At12663,default.search%3Aqualitative%7C%22focus%20group%22%7C%22focus%20groups%22%7Cquestionnaire%7Cquestionnaires%7Cinterview%7Cinterviews%7C%22experiences%22%7C%22lived%20experience%22%7C%22perceptions%22%7C%22perspective%22%7C%22perspectives%22%7C%22attitude%22%7C%22attitudes%22%7C%22beliefs%22%7Cdiary%7Cdiaries,default.search%3Aqualitative%7C%22focus%20group%22%7C%22focus%20groups%22%7C%22semi%20structured%22%7Csemistructured%7Cunstructured%7Cinformal%7Cin-depth%7Cindepth%7Copen%7C%22open%20ended%22%7C%22experiences%22%7C%22lived%20experience%22%7C%22perceptions%22%7C%22perspective%22%7C%22perspectives%22%7C%22attitude%22%7C%22attitudes%22%7C%22beliefs%22%7Cdiary%7Cdiaries,title_and_abstract.search%3A%22young%20people%22%7Cadolescent%7Cyouth%7Cpubertal%7Cpubescent%7C%22pre%20adolescent%22%7C%22Pre%20pubescent%22%7C%22pre%20pubertal%22%7Cteen%7Cpreteen%7Ctweens%7Ctweenage%7Cyouth%7Cyouths%7Cschoolboy%7Cschoolgirl%7C%22school%20aged%22%7C%22young%20person%22%7Cjuvenile%7C%22Boy%22%7C%22boys%22%7C%22children%22%7C%22child%27s%22%7C%22Girl%22%7C%22girls%22%7C%22Minors%22%7C%22preadolescent%22%7C%22Prepubescent%22%7C%22schoolchild%22%7Cpubescent%7C%22early%20adolescent%22,default.search:GB\|Britain\|UK\|"United Kingdom"\|England\|"Northern Ireland"\|"Northern Irish"\|"North Ireland"\|"North Irish"\|Scotland\|Scottish\|Wales\|Welsh\|"english boys"\|"English girls"\|"english school"\|"English children"\|"English teen"\|"English adolescent"\|"English young people"\|"English schoolchild",type:types/book-chapter\|types/book\|types/dissertation\|types/report\|types/other |
| Items coded | 1529 |
| Uploaded documents | 17 |
| Masters of duplicates | 230 |
| Deleted items | 412 |
| Outcomes | 0 |
| Import filter | RIS |
| Is deleted? | false |

| Source name | **Lens** |
| --- | --- |
| Database name/platform | **lens.org** |
| Date of search | Feb 5, 2025 |
| Date of import | Mar 3, 2025 |
| Number items | 1209 |
| Duplicates | 682 |
| Description |  |
| Notes |  |
| Search string | Four searches comprising of a focussed search across all types of records and a broader search to find grey literature. Deduplicated the results before uploading to EPPI-Reviewer.  Results = 1,364 from all searches 1,209 after de-duplication All within year limit 2008-    ((title:("obese" OR "overweight" OR "obesity" OR "body image" OR fat OR fatness OR thin OR thinness OR "Height" OR weight OR bodyweight OR "body hatred" OR "body positive" OR "thin ideal*" OR "fat ideal*" OR "body esteem" OR "body ideal" OR "ideal body" OR "body shape*" OR "body size" OR "body sizes" OR "weight bias*" OR "being fat" OR "being thin" OR "anti fat" OR "body shame" OR "body positive" )) AND (title:(qualitative OR "focus group" OR "focus groups" OR questionnaire OR questionnaires OR interview OR interviews OR "experiences" OR "lived experience*" OR "perceptions" OR "perspective" OR "perspectives" OR "attitude" OR "attitudes" OR "beliefs" OR diary OR diaries) OR abstract:(qualitative OR "focus group" OR "focus groups" OR questionnaire OR questionnaires OR interview OR interviews OR "experiences" OR "lived experience*" OR "perceptions" OR "perspective" OR "perspectives" OR "attitude" OR "attitudes" OR "beliefs" OR diary OR diaries) OR keyword:(qualitative OR "focus group" OR "focus groups" OR questionnaire OR questionnaires OR interview OR interviews) OR field_of_study:(qualitative OR "focus group" OR "focus groups" OR questionnaire OR questionnaires OR interview OR interviews)) AND (title:(qualitative OR "focus group" OR "focus groups" OR "semi structured" OR semistructured OR unstructured OR informal OR in-depth OR indepth OR open OR "open ended" OR "experiences" OR "lived experience*" OR "perceptions" OR "perspective" OR "perspectives" OR "attitude" OR "attitudes" OR "beliefs" OR diary OR diaries) OR abstract:(qualitative OR "focus group" OR "focus groups" OR "semi structured" OR semistructured OR unstructured OR informal OR in-depth OR indepth OR open OR "open ended") OR keyword:(qualitative OR "focus group" OR "focus groups" OR "semi structured" OR semistructured OR unstructured OR informal OR in-depth OR indepth OR open OR "open ended") OR field_of_study:(qualitative OR "focus group" OR "focus groups" OR "semi structured" OR semistructured OR unstructured OR informal OR in-depth OR indepth OR open OR "open ended")) AND (title:("young people*" OR adolescent* OR youth OR pubertal OR pubescent OR "pre adolescent" OR "Pre pubescent" OR "pre pubertal" OR teen* OR preteen* OR tweens OR tweenage* OR youth OR youths OR schoolboy* OR schoolgirl* OR "school aged" OR "young person*" OR juvenile* OR "Boy" OR "boys" OR ("child" NOT ("child birth" OR childbirth)) OR "children*" OR "child's" OR "Girl" OR "girls" OR "Minors" OR "preadolescent" OR "Prepubescent" OR "schoolchild*" OR pubescent OR "early adolescent*") OR abstract: ("young people*" OR adolescent* OR youth OR pubertal OR pubescent OR "pre adolescent" OR "Pre pubescent" OR "pre pubertal" OR teen* OR preteen* OR tweens OR tweenage* OR youth OR youths OR schoolboy* OR schoolgirl* OR "school aged" OR "young person*" OR juvenile* OR "Boy" OR "boys" OR ("child" NOT ("child birth" OR childbirth)) OR "children*" OR "child's" OR "Girl" OR "girls" OR "Minors" OR "preadolescent" OR "Prepubescent" OR "schoolchild*" OR pubescent OR "early adolescent*")) AND (GB OR Britain* OR (British* NOT "British Columbia") OR UK OR "United Kingdom*" OR (England* NOT "New England") OR "Northern Ireland*" OR "Northern Irish*" OR "North Ireland*" OR "North Irish*" OR Scotland* OR Scottish* OR ((Wales OR "South Wales") NOT "New South Wales") OR Welsh* OR "english boys" OR "English girls" OR "english school*" OR "English children*" OR "English teen*" OR "English adolescent*" OR "English young people*" OR "English schoolchild*"))  866 records (lens export 1)   ((abstract:("obese" OR "overweight" OR "obesity" OR "body image" OR fat OR fatness OR thin OR thinness OR "Height" OR weight OR bodyweight OR "body hatred" OR "body positive" OR "thin ideal*" OR "fat ideal*" OR "body esteem" OR "body ideal" OR "ideal body" OR "body shape*" OR "body size" OR "body sizes" OR "weight bias*" OR "being fat" OR "being thin" OR "anti fat" OR "body shame" OR "body positive" )) AND (title:(qualitative OR "focus group" OR "focus groups" OR questionnaire OR questionnaires OR interview OR interviews OR "experiences" OR "lived experience*" OR "perceptions" OR "perspective" OR "perspectives" OR "attitude" OR "attitudes" OR "beliefs" OR diary OR diaries) OR abstract:(qualitative OR "focus group" OR "focus groups" OR questionnaire OR questionnaires OR interview OR interviews OR "experiences" OR "lived experience*" OR "perceptions" OR "perspective" OR "perspectives" OR "attitude" OR "attitudes" OR "beliefs" OR diary OR diaries) OR keyword:(qualitative OR "focus group" OR "focus groups" OR questionnaire OR questionnaires OR interview OR interviews) OR field_of_study:(qualitative OR "focus group" OR "focus groups" OR questionnaire OR questionnaires OR interview OR interviews)) AND (title:(qualitative OR "focus group" OR "focus groups" OR "semi structured" OR semistructured OR unstructured OR informal OR in-depth OR indepth OR open OR "open ended" OR "experiences" OR "lived experience*" OR "perceptions" OR "perspective" OR "perspectives" OR "attitude" OR "attitudes" OR "beliefs" OR diary OR diaries) OR abstract:(qualitative OR "focus group" OR "focus groups" OR "semi structured" OR semistructured OR unstructured OR informal OR in-depth OR indepth OR open OR "open ended") OR keyword:(qualitative OR "focus group" OR "focus groups" OR "semi structured" OR semistructured OR unstructured OR informal OR in-depth OR indepth OR open OR "open ended") OR field_of_study:(qualitative OR "focus group" OR "focus groups" OR "semi structured" OR semistructured OR unstructured OR informal OR in-depth OR indepth OR open OR "open ended")) AND (title:("young people*" OR adolescent* OR youth OR pubertal OR pubescent OR "pre adolescent" OR "Pre pubescent" OR "pre pubertal" OR teen* OR preteen* OR tweens OR tweenage* OR youth OR youths OR schoolboy* OR schoolgirl* OR "school aged" OR "young person*" OR juvenile* OR "Boy" OR "boys" OR ("child" NOT ("child birth" OR childbirth)) OR "children*" OR "child's" OR "Girl" OR "girls" OR "Minors" OR "preadolescent" OR "Prepubescent" OR "schoolchild*" OR pubescent OR "early adolescent*") OR abstract: ("young people*" OR adolescent* OR youth OR pubertal OR pubescent OR "pre adolescent" OR "Pre pubescent" OR "pre pubertal" OR teen* OR preteen* OR tweens OR tweenage* OR youth OR youths OR schoolboy* OR schoolgirl* OR "school aged" OR "young person*" OR juvenile* OR "Boy" OR "boys" OR ("child" NOT ("child birth" OR childbirth)) OR "children*" OR "child's" OR "Girl" OR "girls" OR "Minors" OR "preadolescent" OR "Prepubescent" OR "schoolchild*" OR pubescent OR "early adolescent*")) AND (GB OR Britain* OR (British* NOT "British Columbia") OR UK OR "United Kingdom*" OR (England* NOT "New England") OR "Northern Ireland*" OR "Northern Irish*" OR "North Ireland*" OR "North Irish*" OR Scotland* OR Scottish* OR ((Wales OR "South Wales") NOT "New South Wales") OR Welsh* OR "english boys" OR "English girls" OR "english school*" OR "English children*" OR "English teen*" OR "English adolescent*" OR "English young people*" OR "English schoolchild*"))  refined by doc type: Unknown, dissertation, book chapter, book, report, 213 (lens export 2)    ((abstract:("fat" OR weight OR thin OR height OR muscle* OR muscular* OR body) AND (abstract: (stereotype* OR Stigma OR stigmati* OR discriminate* OR discrimination* OR prejudice* OR bully* OR teasing OR cyberbull* OR dissatisfaction OR dissatisfied OR anxiety OR anxious OR angst OR "peer pressure" OR feelings OR worries OR worry OR concern OR concerns OR "over concern" OR emotions OR emotion OR dysmorph* OR dismorph*))) AND (title:(qualitative OR "focus group" OR "focus groups" OR questionnaire OR questionnaires OR interview OR interviews OR "experiences" OR "lived experience*" OR "perceptions" OR "perspective" OR "perspectives" OR "attitude" OR "attitudes" OR "beliefs" OR diary OR diaries) OR abstract:(qualitative OR "focus group" OR "focus groups" OR questionnaire OR questionnaires OR interview OR interviews OR "experiences" OR "lived experience*" OR "perceptions" OR "perspective" OR "perspectives" OR "attitude" OR "attitudes" OR "beliefs" OR diary OR diaries) OR keyword:(qualitative OR "focus group" OR "focus groups" OR questionnaire OR questionnaires OR interview OR interviews) OR field_of_study:(qualitative OR "focus group" OR "focus groups" OR questionnaire OR questionnaires OR interview OR interviews)) AND (title:(qualitative OR "focus group" OR "focus groups" OR "semi structured" OR semistructured OR unstructured OR informal OR in-depth OR indepth OR open OR "open ended" OR "experiences" OR "lived experience*" OR "perceptions" OR "perspective" OR "perspectives" OR "attitude" OR "attitudes" OR "beliefs" OR diary OR diaries) OR abstract:(qualitative OR "focus group" OR "focus groups" OR "semi structured" OR semistructured OR unstructured OR informal OR in-depth OR indepth OR open OR "open ended") OR keyword:(qualitative OR "focus group" OR "focus groups" OR "semi structured" OR semistructured OR unstructured OR informal OR in-depth OR indepth OR open OR "open ended") OR field_of_study:(qualitative OR "focus group" OR "focus groups" OR "semi structured" OR semistructured OR unstructured OR informal OR in-depth OR indepth OR open OR "open ended")) AND (title:("young people*" OR adolescent* OR youth OR pubertal OR pubescent OR "pre adolescent" OR "Pre pubescent" OR "pre pubertal" OR teen* OR preteen* OR tweens OR tweenage* OR youth OR youths OR schoolboy* OR schoolgirl* OR "school aged" OR "young person*" OR juvenile* OR "Boy" OR "boys" OR ("child" NOT ("child birth" OR childbirth)) OR "children*" OR "child's" OR "Girl" OR "girls" OR "Minors" OR "preadolescent" OR "Prepubescent" OR "schoolchild*" OR pubescent OR "early adolescent*") OR abstract: ("young people*" OR adolescent* OR youth OR pubertal OR pubescent OR "pre adolescent" OR "Pre pubescent" OR "pre pubertal" OR teen* OR preteen* OR tweens OR tweenage* OR youth OR youths OR schoolboy* OR schoolgirl* OR "school aged" OR "young person*" OR juvenile* OR "Boy" OR "boys" OR ("child" NOT ("child birth" OR childbirth)) OR "children*" OR "child's" OR "Girl" OR "girls" OR "Minors" OR "preadolescent" OR "Prepubescent" OR "schoolchild*" OR pubescent OR "early adolescent*")) AND (GB OR Britain* OR (British* NOT "British Columbia") OR UK OR "United Kingdom*" OR (England* NOT "New England") OR "Northern Ireland*" OR "Northern Irish*" OR "North Ireland*" OR "North Irish*" OR Scotland* OR Scottish* OR ((Wales OR "South Wales") NOT "New South Wales") OR Welsh* OR "english boys" OR "English girls" OR "english school*" OR "English children*" OR "English teen*" OR "English adolescent*" OR "English young people*" OR "English schoolchild*"))  refined by doc type: Unknown, dissertation, book chapter, book, report, 236 (lens export 3)   ((title:("fat" OR weight OR thin OR height OR muscle* OR muscular* OR body) AND (title: (stereotype* OR Stigma OR stigmati* OR discriminate* OR discrimination* OR prejudice* OR bully* OR teasing OR cyberbull* OR dissatisfaction OR dissatisfied OR anxiety OR anxious OR angst OR "peer pressure" OR feelings OR worries OR worry OR concern OR concerns OR "over concern" OR emotions OR emotion OR dysmorph* OR dismorph*))) AND (title:(qualitative OR "focus group" OR "focus groups" OR questionnaire OR questionnaires OR interview OR interviews OR "experiences" OR "lived experience*" OR "perceptions" OR "perspective" OR "perspectives" OR "attitude" OR "attitudes" OR "beliefs" OR diary OR diaries) OR abstract:(qualitative OR "focus group" OR "focus groups" OR questionnaire OR questionnaires OR interview OR interviews OR "experiences" OR "lived experience*" OR "perceptions" OR "perspective" OR "perspectives" OR "attitude" OR "attitudes" OR "beliefs" OR diary OR diaries) OR keyword:(qualitative OR "focus group" OR "focus groups" OR questionnaire OR questionnaires OR interview OR interviews) OR field_of_study:(qualitative OR "focus group" OR "focus groups" OR questionnaire OR questionnaires OR interview OR interviews)) AND (title:(qualitative OR "focus group" OR "focus groups" OR "semi structured" OR semistructured OR unstructured OR informal OR in-depth OR indepth OR open OR "open ended" OR "experiences" OR "lived experience*" OR "perceptions" OR "perspective" OR "perspectives" OR "attitude" OR "attitudes" OR "beliefs" OR diary OR diaries) OR abstract:(qualitative OR "focus group" OR "focus groups" OR "semi structured" OR semistructured OR unstructured OR informal OR in-depth OR indepth OR open OR "open ended") OR keyword:(qualitative OR "focus group" OR "focus groups" OR "semi structured" OR semistructured OR unstructured OR informal OR in-depth OR indepth OR open OR "open ended") OR field_of_study:(qualitative OR "focus group" OR "focus groups" OR "semi structured" OR semistructured OR unstructured OR informal OR in-depth OR indepth OR open OR "open ended")) AND (title:("young people*" OR adolescent* OR youth OR pubertal OR pubescent OR "pre adolescent" OR "Pre pubescent" OR "pre pubertal" OR teen* OR preteen* OR tweens OR tweenage* OR youth OR youths OR schoolboy* OR schoolgirl* OR "school aged" OR "young person*" OR juvenile* OR "Boy" OR "boys" OR ("child" NOT ("child birth" OR childbirth)) OR "children*" OR "child's" OR "Girl" OR "girls" OR "Minors" OR "preadolescent" OR "Prepubescent" OR "schoolchild*" OR pubescent OR "early adolescent*") OR abstract: ("young people*" OR adolescent* OR youth OR pubertal OR pubescent OR "pre adolescent" OR "Pre pubescent" OR "pre pubertal" OR teen* OR preteen* OR tweens OR tweenage* OR youth OR youths OR schoolboy* OR schoolgirl* OR "school aged" OR "young person*" OR juvenile* OR "Boy" OR "boys" OR ("child" NOT ("child birth" OR childbirth)) OR "children*" OR "child's" OR "Girl" OR "girls" OR "Minors" OR "preadolescent" OR "Prepubescent" OR "schoolchild*" OR pubescent OR "early adolescent*")) AND (GB OR Britain* OR (British* NOT "British Columbia") OR UK OR "United Kingdom*" OR (England* NOT "New England") OR "Northern Ireland*" OR "Northern Irish*" OR "North Ireland*" OR "North Irish*" OR Scotland* OR Scottish* OR ((Wales OR "South Wales") NOT "New South Wales") OR Welsh* OR "english boys" OR "English girls" OR "english school*" OR "English children*" OR "English teen*" OR "English adolescent*" OR "English young people*" OR "English schoolchild*"))  (lens export 4) 49 records |
| Items coded | 527 |
| Uploaded documents | 6 |
| Masters of duplicates | 27 |
| Deleted items | 682 |
| Outcomes | 0 |
| Import filter | RIS |
| Is deleted? | false |

| Source name | **GoogleScholar** |
| --- | --- |
| Database name/platform |  |
| Date of search | Mar 3, 2025 |
| Date of import | Mar 3, 2025 |
| Number items | 1915 |
| Duplicates | 348 |
| Description |  |
| Notes |  |
| Search string | 1. weight qualitative children UK downloaded first 500 2. "body image" qualitative children UK downloaded first 500 3. "body size" qualitative children UK downloaded first 500 4. weight experiences children UK downloaded first 500 5. "body image" experiences children UK downloaded first 500 6. "body size" experiences children UK downloaded first 500  applied date limit after downloading and deduplicated the results before uploading to EPPI-Reviewer |
| Items coded | 1568 |
| Uploaded documents | 15 |
| Masters of duplicates | 23 |
| Deleted items | 348 |
| Outcomes | 0 |
| Import filter | RIS |
| Is deleted? | false |
