# Supplementary material for: Children’s views of obesity, body size and weight: systematic review of UK qualitative evidence
Source: J Epidemiol Community Health. 2026 Jan 27;80(6):e225045. doi: 10.1136/jech-2025-225045 (PMC13217130; doi:10.1136/jech-2025-225045)
Supplement: online supplemental file 2 [file jech-80-6-s002.docx]

***Children’s views of obesity, body size and weight: Systematic review of UK qualitative evidence. Appendix***

**Results of quality assessment (CASP tool for qualitative studies)**

| **Study identifier** | **1. Aims** | **2. Quals appropriate?** | **3. Design** | **4. Recruitment** | **5. Data collection** | **6. Relationship** | **7. Ethics** | **8. Analysis** | **9. Findings** | **10. Value** |
| --- | --- | --- | --- | --- | --- | --- | --- | --- | --- | --- |
| Baxter | Yes | Yes | CT | Yes | Yes | Yes | Yes | CT | CT | CT |
| Bell | Yes | Yes | Yes | Yes | Yes | Yes | CT | Yes | Yes | Yes |
| Blood | Yes | Yes | Yes | Yes | Yes | No | Yes | Yes | CT | Yes |
| Bromfield | Yes | Yes | Yes | CT | Yes | Yes | Yes | Yes | CT | Yes |
| Charsley | Yes | Yes | CT | Yes | Yes | No | Yes | CT | CT | Yes |
| Clark | CT | Yes | Yes | CT | CT | CT | CT | CT | Yes | CT |
| Conway | Yes | CT | Yes | CT | CT | CT | CT | Yes | Yes | CT |
| Cowley | Yes | Yes | Yes | CT | Yes | CT | CT | Yes | Yes | Yes |
| Dearing | Yes | Yes | Yes | Yes | Yes | CT | Yes | Yes | Yes | Yes |
| Fairbrother | Yes | Yes | Yes | Yes | Yes | Yes | Yes | Yes | Yes | Yes |
| Fielden | Yes | Yes | Yes | CT | Yes | CT | CT | CT | CT | Yes |
| Gemmell | Yes | Yes | Yes | CT | Yes | CT | Yes | Yes | Yes | Yes |
| Gillison | Yes | Yes | Yes | CT | Yes | CT | Yes | CT | No | CT |
| Goldthorpe | Yes | Yes | Yes | No | CT | No | CT | Yes | Yes | CT |
| Hall | Yes | Yes | CT | CT | CT | Yes | Yes | CT | CT | CT |
| Harrold | Yes | CT | CT | CT | CT | CT | CT | Yes | CT | Yes |
| Herbert | Yes | Yes | Yes | CT | Yes | CT | Yes | Yes | Yes | Yes |
| Hooper | Yes | Yes | Yes | Yes | Yes | Yes | Yes | Yes | Yes | Yes |
| Kamal | Yes | Yes | Yes | CT | Yes | CT | Yes | Yes | Yes | Yes |
| Kesten | Yes | Yes | Yes | Yes | Yes | CT | Yes | Yes | Yes | Yes |
| Kumari | Yes | Yes | Yes | Yes | CT | CT | Yes | Yes | Yes | Yes |
| Lewis | Yes | Yes | Yes | Yes | Yes | CT | CT | Yes | Yes | Yes |
| Mansfield | Yes | Yes | Yes | Yes | Yes | No | Yes | Yes | CT | Yes |
| Miller | Yes | Yes | Yes | CT | Yes | CT | CT | CT | Yes | CT |
| Monaghan | No | Yes | CT | No | CT | No | CT | Yes | CT | No |
| Murphy | Yes | Yes | Yes | Yes | Yes | Yes | Yes | Yes | Yes | Yes |
| Newson | CT | Yes | Yes | CT | Yes | CT | CT | Yes | CT | Yes |
| Nnyanzi | Yes | Yes | Yes | CT | Yes | Yes | CT | Yes | Yes | Yes |
| Ogden | Yes | Yes | Yes | Yes | Yes | CT | CT | Yes | Yes | Yes |
| Paddock | Yes | Yes | Yes | CT | Yes | Yes | CT | Yes | Yes | Yes |
| Palmer | Yes | Yes | Yes | Yes | Yes | Yes | Yes | Yes | Yes | Yes |
| Rich | CT | Yes | Yes | CT | CT | CT | CT | CT | Yes | CT |
| Willett | CT | Yes | Yes | CT | CT | Yes | CT | CT | Yes | CT |
| Windram-Geddes | Yes | Yes | Yes | No | Yes | Yes | Yes | CT | Yes | Yes |
