# Supplementary material for: Children’s views of obesity, body size and weight: systematic review of UK qualitative evidence
Source: J Epidemiol Community Health. 2026 Jan 27;80(6):e225045. doi: 10.1136/jech-2025-225045 (PMC13217130; doi:10.1136/jech-2025-225045)
Supplement: online supplemental file 3 [file jech-80-6-s003.docx]

***Children’s views of obesity, body size and weight: Systematic review of UK qualitative evidence. Appendix***

GRADE-Cerqual assessment

|  | **Review finding** | **Studies contribut–ing to the review finding** | **Methodo–logical Limitations** | **Adequacy of Data** | **Coherence** | **Relevance** | **CERQual Assess–ment** | **Explanation of confidence in the evidence assessment** |
| --- | --- | --- | --- | --- | --- | --- | --- | --- |
| 1 | Health impacts of weight. Children see overweight and obesity as 'unhealthy' in a general sense, and identify specific poorer health outcomes. | Baxter, Charsley, Fairbrother, Fielden, Hooper, Kumari, Mansfield, Newson, Palmer, Rich | Minor concerns around ethics (3 studies), data analysis (4 studies) and findings (4 studies) | No or very minor concerns. Data come from a range of populations (age, weight status) | Minor concerns. While there are different views underlying the link, the main finding is the link itself rather than participants' causal theories of health outcomes (which are often vague or absent) | Minor concerns. Data on general concepts of health were outside the scope of this review. | High | Minor concerns on methodology, adequacy and coherence but no major limitations |
| 2 | Appearance. Children feel that overweight 'looks bad' or is less attractive. | Baxter, Bell, Cowley, Hooper, Mansfield, Newson | No or very minor concerns | Major concerns. There is a disproportion between the sparse data directly addressing this theme, and the focus on it by several study authors. | Moderate concerns. The findings are not unidirectional and it is unclear how far appearance is an independent driver of perceptions | No or very minor concerns | Low | Concerns around adequacy and coherence, and in general limited data |
| 3 | Health impacts of weight. Children think that underweight can also have negative health impacts. | Baxter, Cowley, Fairbrother, Fielden, Hooper, Monaghan | Minor concerns around sampling and recruitment (3 studies), ethics (3 studies) and findings (3 studies) | Moderate concerns. Data are relatively sparse and appear to come from only one or two participants per study | Moderate concerns as there appear to be a range of different perceptions underlying this finding | No or very minor concerns | Low | Concerns around adequacy and coherence, and in general limited data |
| 4 | Physical abilities. Children without overweight think that people with overweight and obesity have limited capacities to engage in physical activity or sport, and more generally to participate in 'normal' life. | Baxter, Bromfield, Charsley, Dearing, Fairbrother, Kamal, Mansfield, Murphy, Palmer, Rich | Minor concerns around sampling and recruitment (3 studies), data analysis (3 studies) and findings (3 studies) | Minor concerns. Data come from across the age range. The theme is mostly not explicitly a focus for primary study authors but the data are rich | No or very minor concerns. Perceptions appear reasonably consistent | No or very minor concerns | High | Minor concerns on methodology, adequacy and coherence but no major limitations |
| 5 | Social and emotional impacts. Children without overweight think that those with overweight are likely to be teased, bullied or called names, and sometimes to be excluded from social activities. | Baxter, Charsley, Clark, Cowley, Fairbrother, Gillison, Hall, Hooper, Kesten, Mansfield, Murphy | Minor concerns around sampling and recruitment (4 studies), data analysis (5 studies) and findings (4 studies) | No or very minor concerns. Data come from across the age range. Data are rich and refer to specific experiences. Participants recognise prevalence of bullying even when personally rejecting it | No or very minor concerns. The finding is consistent with the data from children with overweight or obesity (finding 12). | No or very minor concerns | High | Minor concerns on methodology, adequacy and coherence but no major limitations |
| 6 | Causes - dietary. Children see overeating as a main cause of overweight. | Baxter, Charsley, Cowley, Fairbrother, Fielden, Gemmell, Gillison, Kamal, Kesten, Kumari, Mansfield, Murphy, Rich, Windram-Geddes | Moderate concerns around sampling and recruitment (7 studies), ethics (3 studies), data analysis (6 studies) and findings (4 studies) | Minor concerns. Data are not very rich but that is the nature of the theme, and come from a range of populations | Minor concerns. Findings are generally consistent although different food types are mentioned. | Minor concerns. Data on diet as such were outside the scope of this review | Moderate | Some concerns around methodology, but only minor limitations otherwise |
| 7 | Causes - physical activity. Children see the lack of physical activity as a cause of overweight. | Baxter, Bell, Clark, Fairbrother, Fielden, Gemmell, Gillison, Herbert, Hooper, Kamal, Kesten, Mansfield, Rich, Windram-Geddes | Moderate concerns around sampling and recruitment (8 studies), data analysis (6 studies) and findings (3 studies) | Minor concerns. Data are reasonably rich. Only one study explores underlying theories in detail, but that is not the main point of the finding | Minor concerns. There is sometimes unclarity as to whether participants are referring to lack of activity as a cause or a consequence of overweight, but this is a finding in itself | Minor concerns. Data on physical activity as such were outside the scope of this review | Moderate | Some concerns around methodology, but only minor limitations otherwise |
| 8 | Body ideals. Some children want to lose weight or become more muscular, but most reject extremely underweight body shapes. | Clark, Fairbrother, Kesten, Monaghan, Palmer, Rich, Windram-Geddes | Moderate concerns around sampling and recruitment (4 studies), data collection (3 studies), ethics (3 studies) and data analysis (3 studies) | Major concerns. Most of the data come from older girls, and some data raise concerns about social desirability bias and/or researchers' self-positioning (in studies with an explicit focus on this theme) | Moderate concerns. Expressed ideals are somewhat varied (outside the studies explicitly focusing on this theme) and may depend on context, and it is unclear how far participants subscribe to specific body ideals | No or very minor concerns | Low | Concerns around methodology, adequacy and coherence |
| 9 | Experiences of weight loss. Children report trying to lose weight, sometimes leading to disordered eating (either for themselves or for peers). | Blood, Clark, Fairbrother, Kesten, Miller, Monaghan, Nnyanzi, Rich, Willett, Windram-Geddes | Moderate concerns around sampling and recruitment (7 studies), data collection (4 studies), ethics (7 studies) and data analysis (5 studies) | Moderate concerns. Data are fairly sparse and not explored in depth. | Moderate concerns. Some data suggest disparity between reports of participants' own behaviour and their perceptions of others' (this is arguably a finding in itself). A few participants report aiming to gain weight. | Moderate concerns. Data on disordered eating as such were outside the scope of this review | Low | Concerns around methodology, adequacy and coherence |
| 10 | Body ideals. Media and social media present body ideals which are sometimes unrealistic, promote weight loss, and also represent extreme obesity in a sensationalistic way. | Bell, Conway, Fairbrother, Hall, Herbert, Kesten, Kumari, Mansfield, Miller, Monaghan, Paddock, Rich, Willett | Moderate concerns around sampling and recruitment (8 studies), data collection (6 studies), ethics (5 studies) and data analysis (4 studies) | Moderate concerns. Data are fairly sparse and not explored in depth, or related to specific experiences. Data mostly come from older children | Moderate concerns. Participants were referring to a range of different types of content, and data which report experiences in more depth diverge from generic high-level perceptions | No or very minor concerns | Low | Concerns around methodology, adequacy and coherence |
| 11 | "Fat talk". Children report parents encouraging them to lose (or gain) weight. They observe that adults around them are often dissatisfied with their body shape, or have negative attitudes to overweight generally. | Charsley, Clark, Fairbrother, Fielden, Gemmell, Herbert, Kesten, Kumari, Lewis, Monaghan, Newson, Palmer | Moderate concerns around sampling and recruitment (6 studies), data collection (3 studies), ethics (4 studies), data analysis (3 studies) and findings (3 studies) | Minor concerns. Data are reasonably rich and come from a range of populations | Minor concerns. Findings are generally consistent. A few participants report being encouraged to gain weight. | No or very minor concerns | Moderate | Some concerns around methodology, but only minor limitations otherwise |
| 12 | Experiences of weight-related bullying. Children with overweight or obesity report extensive experiences of weight-related bullying, sometimes with serious impacts on wellbeing. | Gemmell, Hall, Kumari, Newson | Moderate concerns around sampling and recruitment (3 studies) | Minor concerns. Data are rich and refer to specific experiences. The studies all recruited from community-based weight management interventions. Most data concern older children | No or very minor concerns. This finding is consistent with the data from children without overweight (finding 5). | No or very minor concerns | Moderate | Some concerns around methodology, but only minor limitations otherwise |
| 13 | Experiences of physical activity. Children with overweight or obesity report that others' attitudes can be a barrier to participating in activities. | Gemmell, Hall, Kumari, Newson | Moderate concerns around sampling and recruitment (3 studies) | Moderate concerns. The data are fairly sparse but come from observations as well as interviews. Issues with sampling and population as for finding 12 | Moderate concerns. Participants' experiences were variable. | No or very minor concerns | Low | Concerns around methodology, adequacy and coherence |
