# Supplementary material for: Children’s views of obesity, body size and weight: systematic review of UK qualitative evidence
Source: J Epidemiol Community Health. 2026 Jan 27;80(6):e225045. doi: 10.1136/jech-2025-225045 (PMC13217130; doi:10.1136/jech-2025-225045)
Supplement: online supplemental file 4 [file jech-80-6-s004.docx]

***Children’s views of obesity, body size and weight: Systematic review of UK qualitative evidence. Appendix***

Studies excluded at full-text screening

| **Reference** | **Exclusion criterion** |
| --- | --- |
| Benninghoven Dieter and Tetsch Nina; Jantschek Gunter;. (2008). Patients with eating disorders and their siblings. An investigation of body image perceptions.. European child & adolescent psychiatry, 17(2), pp.118-26. | EX1 |
| Adams M and Robling M; Grainger J; Tomlins J; Johnson A; Morris S; Velangi M; Jenney M;. 2016. "Quality of life Evaluation in patients receiving Steroids (the QuESt tool): initial development in children and young people with acute lymphoblastic leukaemia.". Archives of disease in childhood 101(3):241-6. | EX2 |
| Allen Kerry. 2011. "Managing Prader-Willi syndrome in families: an embodied exploration.". Social science & medicine (1982) 72(4):460-8. | EX3 |
| Anonymous. 2017. "Somebody like me: a report investigating the impact of body image anxiety on young people in the UK". :44. | EX2 |
| Arai L and Panca M; Morris S; Curtis-Tyler K; Lucas PJ;. 2015. Time, monetary and other costs of participation in family-based child weight management interventions: qualitative and systematic review evidence. : journals.plos.org. | EX3 |
| Banks Jonathan and Cramer Helen; Sharp Deborah J; Shield Julian Ph; Turner Katrina M;. 2014. "Identifying families' reasons for engaging or not engaging with childhood obesity services: A qualitative study.". Journal of child health care : for professionals working with children in the hospital and community 18(2):101-10. | EX3 |
| Bauer Katherine W and Branch Jacqueline M; Appugliese Danielle P; Pesch Megan H; Miller Alison L; Lumeng Julie C; Kaciroti Niko;. 2021. "Emerging Ideas. How Do Low-Income Mothers Talk to Children About Weight and Body Shape?". Family Relations 70:1477-1484. | EX2 |
| Bell Sarah L and Audrey Suzanne; Cooper Ashley R; Noble Sian; Campbell Rona;. 2017. "Lessons from a peer-led obesity prevention programme in English schools.". Health promotion international 32(2):250-259. | EX3 |
| Bhagat Krishna and Howard Donna E;. 2018. "The Dominant Obesity Discourse Versus Children’s Conceptualizations of Health: A Comparison Through Dialogue and Drawings". Qualitative Health Research 28:1157-1170. | EX5 |
| Boddy Lynne M and Knowles Zoe R; Davies Ian G; Warburton Genevieve L; Mackintosh Kelly A; Houghton Laura; Fairclough Stuart J;. 2012. "Using formative research to develop the healthy eating component of the CHANGE! school-based curriculum intervention.". BMC public health 12:710. | EX3 |
| Braden Abby and Crow Scott; Boutelle Kerri;. (2014). Child self-reported motivations for weight loss: impact of personal vs. social/familial motives on family-based behavioral weight loss treatment outcomes. Eating and Weight Disorders - Studies on Anorexia Bulimia and Obesity, 20(2), pp.205-213. | EX1 |
| Brockman Rowan and Fox Kenneth; Jago Russell;. 2011. "What is the meaning and nature of active play for today's children in the UK?". International Journal of Behavioral Nutrition and Physical Activity 8:15-15. | EX3 |
| Brockman Rowan and Jago Russell; Fox Kenneth; Thompson Janice; Cartwright Kim; Page Angie;. 2009. ""Get off the sofa and go and play": Family and socioeconomic influences on the physical activity of 10–11 year old children". BMC Public Health 9:. | EX3 |
| Burnette C Blair and Kwitowski Melissa A; Mazzeo Suzanne E;. 2017. ""I don't need people to tell me I'm pretty on social media:" A qualitative study of social media and body image in early adolescent girls.". Body image 23:114-125. | EX5 |
| Calvert S and Dempsey RC; Povey R;. 2020. "A qualitative study investigating food choices and perceived psychosocial influences on eating behaviours in secondary school students". BRITISH FOOD JOURNAL 122(4):1027-1039. | EX3 |
| Catalbas Meltem. 2022. "A Focused Ethnographic Study of Children's Perspectives and Experiences of Being Obese and Participating in an Obesity Intervention Program". , University of Sheffield (United Kingdom). | EX2 |
| Clark MI and Spence JC; Holt NL;. 2011. "In the shoes of young adolescent girls: Understanding physical activity experiences through interpretive description". Qualitative research in sport … :. | EX5 |
| Clarke Joanne L and Griffin Tania L; Lancashire Emma R; Adab Peymane; Parry Jayne M; Pallan Miranda J;. 2015. "Parent and child perceptions of school-based obesity prevention in England: a qualitative study.". BMC public health 15:1224. | EX3 |
| Clarke Joanne Louise. 2016. "The role of the primary school in preventing childhood obesity". , University of Birmingham. | EX3 |
| Clarke Rebecca and Heath Gemma; Nagakumar Prasad; Pattison Helen; Farrow Claire;. 2022. ""He's not fat, he just has asthma": a qualitative study exploring weight management in families living with pediatric asthma.". The Journal of asthma : official journal of the Association for the Care of Asthma 59(9):1750-1757. | EX2 |
| Clarke Rebecca. 2020. "Weight Management in Children Who Have Asthma and Comorbid Overweight/Obesity". , Aston University (United Kingdom). | EX3 |
| Copperstone CS and Douglas FCG; Craig LCA; Jackson DM;. 2018. "Parents and Schoolchildren Talking about Food and Drink Choices - A Focus Group Study". HEALTH BEHAVIOR AND POLICY REVIEW 5(1):28-39. | EX3 |
| Cox Jennifer and Searle Aidan; Thornton Gail; Hamilton‐Shield Julian; Hinton Elanor;. 2022. "Integrating COM-B and the Person-Based Approach to develop an ACT based therapy programme to raise self-determination in adolescents with obesity". Research Square (Research Square) :. | EX3 |
| Cox Jennifer S and Searle Aidan J; Hinton Elanor C; Giri Dinesh; Shield Julian P H;. 2021. "Perceptions of non-successful families attending a weight-management clinic.". Archives of disease in childhood 106(4):377-382. | EX2 |
| Craddock N and Smith HG; Garbett KM; Alleva JM;. 2025. Functionality appreciation in young children. : Elsevier. | EX3 |
| Credos. (2016). Picture of health?. , , pp.23. | EX1 |
| Curtis Penny. 2008. "The experience of young people with obesity in secondary school: some implications for the healthy school agenda". Health and Social Care in the Community, Vol 16 No 4 Jul 2008 :9. | EX2 |
| Damant Estelle. 2020. "Grade A health: an exploration of the social construction of health and ability within secondary school physical education". :. | EX2 |
| Datta N and Foukal M; Erwin S; Hopkins H; Tchanturia K; Zucker N;. 2021. "A mixed-methods approach to conceptualizing friendships in anorexia nervosa". PLOS ONE 16(9):. | EX3 |
| Dohnt H K and Tiggemann M;. (2008). Promoting positive body image in young girls: An evaluation of 'Shapesville'. European Eating Disorders Review, 16(3), pp.222-233. | EX1 |
| Drage Lucy Amelia. 2014. "Body esteem and education: How does body esteem develop in children and young people and what can schools do to promote positive body esteem?". , University of Exeter (United Kingdom). | EX3 |
| Drummond Murray. 2012. "Boys' Bodies in Early Childhood". Australasian Journal of Early Childhood 37(4):107-114. | EX5 |
| Edwards S and Skouteris H; Rutherford L; Cutter-Mackenzie A;. 2013. "'It's All About Ben10[TM]': Children's Play, Health and Sustainability Decisions in the Early Years". Early Child Development and Care 183(2):280-293. | EX2 |
| Eyre E. 2014. "Environmental influences on physical activity and weight status in children from deprived multi-ethnic backgrounds in coventry". , Coventry University (United Kingdom). | EX3 |
| Eyre Emma and Duncan Michael; Birch Samantha; Cox Val;. 2013. "Environmental and school influences on physical activity in South Asian children from low socio-economic backgrounds". Journal of Child Health Care 19:345-358. | EX3 |
| Fairbrother Hannah and Ellis Katie;. (2016). Everyday Family Food Practices. | EX1 |
| Farman R and Fitzgerald H; Radley D;. 2019. "Weight management provision in a special school: Experiences of disabled children and their families". 26th European Congress on Obesity. Glasgow United Kingdom. 12(Supplement 1):268. | EX3 |
| Flint Tori K. 2020. "Children's Critical Reflections on Gender and Beauty through Responsive Play in the Classroom Context". Early Childhood Education Journal 48(6):739-749. | EX5 |
| Freire Kate and Pope Rodney; Coyle Julia;. 2018. "What are the drivers of cross-generational physical activity? Exploring the experiences of children and parents". Journal of Public Health 27:591-601. | EX3 |
| Fremont Ettya R and Friedrich Elizabeth A; Grimberg Adda; Miller Victoria A;. 2024. "Youth and parent perceptions of youth decision-making roles regarding evaluation for short stature". Children's Health Care 53:148-162. | EX3 |
| Gadsby E W and Hotham S; Eida T; Lawrence C; Merritt R;. 2020. "Impact of a community-based pilot intervention to tackle childhood obesity: a 'whole-system approach' case study.". BMC public health 20(1):1818. | EX3 |
| Girlguiding. (2023). Girls' attitudes survey 2023: girls' lives over 15 years.. London: Girlguiding, pp.35. Available at: https://www.girlguiding.org.uk/globalassets/docs-and-resources/research-and-campaigns/girls-attitudes-survey-2023.pdf. | EX1 |
| Grimmett Chloe and Croker Helen; Carnell Susan; Wardle Jane;. 2008. "Telling parents their child's weight status: psychological impact of a weight-screening program.". Pediatrics 122(3):e682-8. | EX3 |
| Guest E and Jarman H; Sharratt N; Williamson H; White P; Harcourt D; Slater A;. 2021. "'Everybody's Different: The Appearance Game'. A randomised controlled trial evaluating an appearance-related board game intervention with children aged 9-11 years.". Body image 36:34-44. | EX3 |
| Handford Charlotte M and Rapee Ronald M; Fardouly Jasmine;. (2018). The influence of maternal modeling on body image concerns and eating disturbances in preadolescent girls.. Behaviour research and therapy, 100, pp.17-23. | EX1 |
| Harding Sarah and Smith Laura Mazzoli;. 2022. "Freedom through constraint: Young women's embodiment, space and wellbeing during lockdown.". Wellbeing, space and society 3:100101. | EX2 |
| Harkensee Christian and Andrew Rachel;. 2021. "Health needs of accompanied refugee and asylum-seeking children in a UK specialist clinic.". Acta paediatrica (Oslo, Norway : 1992) 110(8):2396-2404. | EX2 |
| Harriger JA and Thompson JK;. (2012). Psychological consequences of obesity: Weight bias and body image in overweight and obese youth. International Review of Psychiatry, , pp.. | EX1 |
| Harriger Jennifer and Trammell Janet; Wick Madeline; Luedke Madeline;. (2019). Gender and age differences in pre-schoolers' weight bias beliefs and behavioural intentions.. The British journal of developmental psychology, 37(4), pp.461-465. | EX1 |
| Harrison Sarah and Rowlinson Madaleine; Hill Andrew J;. (2016). "No fat friend of mine": Young children's responses to overweight and disability.. Body image, 18, pp.65-73. | EX1 |
| Hart CS and Page A;. 2020. "The capability approach and school food education and culture in England: 'gingerbread men ain't gonna get me very far'". CAMBRIDGE JOURNAL OF EDUCATION 50(6):673-693. | EX3 |
| Harwood Valerie. 2012. "Neither Good nor Useful: Looking Ad Vivum in Children's Assessments of Fat and Healthy Bodies". Discourse: Studies in the Cultural Politics of Education 33(5):693-711. | EX5 |
| Henderson Emily Jacqueline. 2010. "Adiposity in british pakistani and white british school children aged 7-11 years living in middlesbrough, uk: associations with ethnicity, generation, and birth weight". , University of Durham (United Kingdom). | EX3 |
| Hill J. 2015. "'If you miss the ball, you look like a total muppet!'Boys investing in their bodies in physical education and sport". Sport, Education and Society :. | EX2 |
| Hill Joanne. 2015. "Girls' Active Identities: Navigating Othering Discourses of Femininity, Bodies and Physical Education". Gender and Education 27(6):666-684. | EX2 |
| Holt Nicholas L and Neely Kacey C; Newton Amanda S; Knight Camilla J; Rasquinha Allison; Ambler Kathryn A; Spence John C; Ball Geoff D C;. 2015. "Families' Perceptions of and Experiences Related to a Pediatric Weight Management Intervention: A Qualitative Study.". Journal of nutrition education and behavior 47(5):427-31.e1. | EX3 |
| Holub SC. (2008). Individual differences in the anti-fat attitudes of preschool-children: The importance of perceived body size. Body image, , pp.. | EX1 |
| Holub Shayla C and Tan Cin Cin; Patel Sanobar L;. (2011). Factors associated with mothers' obesity stigma and young children's weight stereotypes. Journal of Applied Developmental Psychology, 32, pp.118-126. | EX1 |
| Howells Kristy and Bowen J; Mannion K; McMartin C;. 2019. "The adoption of daily mile as an active mile initiative: the children’s and teachers’ voices". : . | EX3 |
| Institute Joanna Briggs. (2011). Children's views about obesity, body size, shape and weight; Inequalities and the mental health of young people: a systematic review of secondary school‐based …. Journal of Advanced Nursing, , pp.. | EX1 |
| Jassi Amita D and Baloch Aysha; Thomas-Smith Kike; Lewis Angela;. 2020. "Family accommodation in pediatric body dysmorphic disorder: A qualitative study.". Bulletin of the Menninger Clinic 84(4):319-336. | EX3 |
| Jones Catrin P and Armstrong-Moore Roxanne; Penney Tarra L; Cummins Steven; Armitage Sofie; Adams Jean; White Martin;. 2022. "Adolescents' perspectives on soft drinks after the introduction of the UK Soft Drinks Industry Levy: A focus group study using reflexive thematic analysis.". Appetite 179:106305. | EX3 |
| Keenaghan S and Polaskova M; Thurlbeck S;. (2020). Alice in Wonderland: The effects of body size and movement on children's size perception and bodily awareness in virtual reality. : scholar.archive.org. | EX1 |
| Kehler Michael P. H. D and Atkinson Michael P. H. D;. 2015. "The Space Between: Negotiating Male Subjectivities in Physical Education Research". International Journal of Men's Health 14:259-272. | EX5 |
| Kemp BJ and Thompson DR; McGuigan K; Watson CJ; Heron N; Woodside J; Devaney FK; Harrison N; Neill DT; Cutting D; Ski CF;. 2023. "Families' expectations of an eHealth family-based cardiovascular disease-risk reduction programme". EUROPEAN JOURNAL OF CARDIOVASCULAR NURSING 22(1):82-88. | EX3 |
| Ketteridge Asha. 2008. "Exploring the reasons why adolescents participate in physical activity and identifying strategies that facilitate their involvement in such activity". Australian Occupational Therapy Journal 55(4):. | EX3 |
| Kilmurray M and Collins SC; Caterson ID; Hill AJ;. (2020). Is Weight Bias Evident in Peer Interactions Between Young and Older Children?. OBESITY, 28(2), pp.333-338. | EX1 |
| Kilmurray Michelle. (2017). Peer-to-Peer Responses to Body Shape in Young Children. . University of Leeds. | EX1 |
| Kime N H and McKenna J; Griffiths C; Rivett M; Gately P;. 2018. "A qualitative evaluation of healthy weight services in a local authority in England". Health Education Journal 77(8):939-951. | EX2 |
| Kirby Joanna and Tibbins Carly; Callens Claire; Lang Beckie; Thorogood Margaret; Tigbe William; Robertson Wendy;. 2012. "Young People's Views on Accelerometer Use in Physical Activity Research: Findings from a User Involvement Investigation.". ISRN obesity 2012:948504. | EX3 |
| Kirby Joanne. 2013. "Influences on young people's physical activity in Scotland : a socio-ecological approach". , . | EX3 |
| Kornilaki Ekaterina N. (2015). Obesity Bias in Children: The Role of Actual and Perceived Body Size. Infant and Child Development (Online), 24, pp.365-378. | EX1 |
| Krayer A. 2008. "Social comparison and body image in adolescence: a grounds theory approach". Health Education Research 23(5):. | EX2 |
| Ktenidis Antonios. 2020. "‘Short’ Stories of Young People with Restricted Growth of Their Schooling Experiences (Secondary Education) in the United Kingdom". , . | EX2 |
| Land Nicole. (2022). Tending, Counting and Fitting with Post-Developmental Fat(s) in Early Childhood Education. Contemporary Issues in Early Childhood, 23(1), pp.80-95. | EX1 |
| Law Catherine and Cole Tim; Cummins Steven; Fagg James; Morris Stephen; Roberts Helen;. 2014. "A pragmatic evaluation of a family-based intervention for childhood overweight and obesity". :. | EX3 |
| Lee Pi‐Hsia and Lai Hsiang-Ru; Chou Yu‐Hua; Chang Lu‐I; Chang Wen-Ying;. 2009. "Perceptions of Exercise in Obese School-Aged Children". Journal of Nursing Research 17:170-178. | EX5 |
| Lewis K and Fraser C; Manby M;. 2014. "'Is it worth it?'A qualitative study of the beliefs of overweight and obese physically active children". Journal of Physical … :. | EX3 |
| Lewis Kiara. 2015. "Engaging children and young people in physical activity". , University of Huddersfield (United Kingdom). | EX3 |
| Lloyd Jenny and Dean Sarah; Creanor Siobhan; Abraham Charles; Hillsdon Melvyn; Ryan Emma; Wyatt Katrina M;. 2017. "Intervention fidelity in the definitive cluster randomised controlled trial of the Healthy Lifestyles Programme (HeLP) trial: findings from the process evaluation.". The international journal of behavioral nutrition and physical activity 14(1):163. | EX3 |
| Lunde C and Gattario KH;. 2017. "Performance or appearance? Young female sport participants' body negotiations". Body image :. | EX5 |
| Lyles Annmarie M. S. R. N and Riesch Susan K. PhD R. N. Faan; Sanders Linda M. P. H; Sass-DeRuyter Suzanne M. A. P. N. P; Birchmeier Becky M. S. R. N; Kotula Kelly B. S. R. N;. 2012. "How Do Treatment-Seeking Overweight Youth and Their Parents Describe Weight Promoting Factors in Their Family?". Journal of Community Health Nursing 29:187. | EX5 |
| Macdiarmid Jennie I and Wills Wendy J; Masson Lindsey F; Craig Leone C A; Bromley Catherine; McNeill Geraldine;. (2015). Food and drink purchasing habits out of school at lunchtime: a national survey of secondary school pupils in Scotland.. The international journal of behavioral nutrition and physical activity, 12, pp.98. | EX1 |
| Mackintosh KA and Knowles ZR; Ridgers ND; Fairclough SJ;. 2011. "Using formative research to develop CHANGE!: a curriculum-based physical activity promoting intervention". BMC PUBLIC HEALTH 11:. | EX3 |
| Martin Anne. 2014. "Lifestyle interventions to improve educational attainment in overweight or obese children". , The University of Edinburgh (United Kingdom). | EX2 |
| Matheson Emily and Schneider Jennifer; Tinoco Aline; Gentili Claudio; Silva-Breen Hannah; LaVoi Nicole; White Paul; Diedrichs Phillippa;. (2023). The co-creation, initial piloting, and protocol for a cluster randomised controlled trial of a coach-led positive body image intervention for girls in sport. BMC Public Health, 23, pp.. | EX1 |
| Maynard Maria and Baker Graham; Harding Seeromanie;. (2017). Exploring childhood obesity prevention among diverse ethnic groups in schools and places of worship: Recruitment, acceptability and feasibility of data collection and intervention components.. Preventive medicine reports, 6, pp.130-136. | EX1 |
| McDougall J and Duncan M J;. 2008. "Children, video games and physical activity: An exploratory study". International Journal on Disability and Human Development 7(1):89-94. | EX3 |
| McEvoy Claire and Lawton Julia; Kee Frank; Young Ian; Woodside Jayne; McBratney J; McKinley Michelle;. 2014. "Adolescents' views about a proposed rewards intervention to promote healthy food choice in secondary school canteens". Health Education Research 29:799-811. | EX3 |
| McLaughlin Janice. 2017. "The medical reshaping of disabled bodies as a response to stigma and a route to normality". Medical Humanities 43:244. | EX3 |
| Monaghan Lee F. 2014. "Civilising recalcitrant boys' bodies: Pursuing social fitness through the anti-obesity offensive.". Sport, Education and Society 19(6):691-711. | EX2 |
| Morano M and Colella D; Capranica L;. (2011). Body image, perceived and actual physical abilities in normal-weight and overweight boys involved in individual and team sports. Journal of Sports Sciences, , pp.. | EX1 |
| Morgan K and Van Godwin J; Darwent K; Fildes A;. 2019. "Formative research to develop a school-based, community-linked physical activity role model programme for girls: CHoosing Active Role Models to INspire Girls (CHARMING)". BMC PUBLIC HEALTH 19:. | EX3 |
| Murray Aisling and Smith Scott; Dominie Mahala; Nikolajeva Milena; Porricelli Daniele; van Loggerenberg Francois; Ougrin Dennis; Lau Jennifer Y F;. 2024. "Children's emerging concepts of resilience: insights from using body mapping in an East London cohort sample of 7-10-year-old children.". Frontiers in psychology 15:1408771. | EX3 |
| Nabors Laura and Thomas Myra; Vaughn Lisa; Adams Ryan; Amaral Joe; Olsen Brian T;. 2011. "Children’s Attitudes About an Overweight or Non-overweight Weight Victim". Journal of Developmental and Physical Disabilities 23:87-98. | EX5 |
| Nally Sarah and Ridgers Nicola; Gallagher Alison; Murphy Marie; Salmon Jo; Carlin Angela;. 2022. "“When You Move You Have Fun”: Perceived Barriers, and Facilitators of Physical Activity From a Child's Perspective". Frontiers in Sports and Active Living 4:. | EX3 |
| O'Connell R and Brannen J;. 2014. "Children's food, power and control: Negotiations in families with younger children in England". CHILDHOOD-A GLOBAL JOURNAL OF CHILD RESEARCH 21(1):87-102. | EX3 |
| O'Connor Jacklin and Golley Rebecca; Perry Rebecca; Magarey Anthea; Truby Helen;. (2014). A longitudinal investigation of overweight children's body perception and satisfaction during a weight management program. Appetite, 85, pp.48-51. | EX1 |
| Owen Sarah E and Sharp Deborah J; Shield Julian P; Turner Katrina M;. 2009. "Childrens' and parents' views and experiences of attending a childhood obesity clinic: A qualitative study.". Primary Health Care Research and Development 10(3):236-244. | EX2 |
| Ozdamar ertekin and Zeynep; Atik Deniz;. 2013. "Children's perception of food and healthy eating: dynamics behind their food preferences". International journal of consumer studies 37:59-65. | EX3 |
| Pallan Miranda and Hurley Kiya L; Griffin Tania; Lancashire Emma; Blissett Jacqueline; Frew Emma; Gill Paramjit; Hemming Karla; Jackson Louise; Jolly Kate; McGee Eleanor; Parry Jayne; Thompson Janice L; Adab Peymane;. 2018. "A cluster-randomised feasibility trial of a children's weight management programme: the Child weigHt mANaGement for Ethnically diverse communities (CHANGE) study.". Pilot and feasibility studies 4:175. | EX3 |
| Paxton SJ and McLean SA; Rodgers RF;. (2022). “My critical filter buffers your app filter”: Social media literacy as a protective factor for body image. Body Image, , pp.. | EX1 |
| Pearce A and Kirk C; Cummins S; Collins M; Elliman D; Connolly A M; Law C;. 2009. "Gaining children's perspectives: a multiple method approach to explore environmental influences on healthy eating and physical activity.". Health & place 15(2):614-621. | EX3 |
| Perez Marisol, Kroon Van Diest and Ashley M; Smith Haylie; Sladek Michael R;. (2018). Body Dissatisfaction and Its Correlates in 5- to 7-Year-Old Girls: A Social Learning Experiment. Journal of Clinical Child and Adolescent Psychology, 47, pp.757-769. | EX1 |
| Pescott Claire. 2022. "“Me, My Selfie and I”: An Exploration of Subjectivity and Identity Portrayal in the Social Media Use of 10-And 11-Year-Olds". , University of South Wales (United Kingdom). | EX3 |
| Pescott Claire. 2024. "Self-presentation within children's digital spaces.". Children, young people and online harms: Conceptualisations, experiences and responses. :61-83. | EX3 |
| Pescud M and Pettigrew S; McGuigan MR; Newton RU;. 2010. "Factors influencing overweight children's commencement of and continuation in a resistance training program". BMC PUBLIC HEALTH 10:. | EX3 |
| Pickard Angela. 2013. "Ballet body belief: Perceptions of an ideal ballet body from young ballet dancers.". Research in Dance Education 14(1):3-19. | EX2 |
| Pilcher Jane. 2010. "What not to wear? Girls, clothing and 'showing' the body". Children and society 24:461-470. | EX3 |
| Pittson Helen and Wallace Louise;. (2011). Using intervention mapping to develop a family-based childhood weight management programme.. Journal of health services research & policy, 16 Suppl 1, pp.2-7. | EX1 |
| Pizzirani Bengianni et al;. (2022). Healthy lifestyle programs in out-of-home care: implementing preventative trauma-informed approaches at scale. Australian Social Work, 75(1), pp.5-18. | EX1 |
| Povey R and Cowap L; Gratton L;. 2016. "“They said I'ma square for eating them” Children's beliefs about fruit and vegetables in England". British Food Journal :. | EX3 |
| Pratt Keeley J and Lamson Angela L; Radley Sarah V;. 2015. "The Self-Reported Strengths and Concerns of Treatment-seeking Obese Youth and Their Caregivers". Children's Health Care 44:87. | EX5 |
| Puglisi L and Okely A; Vialle W; Pearson P;. 2010. "A naturalistic inquiry into the daily lives of obese children". 11th International Congress on Obesity, ICO 2010. Stockholm Sweden. 11(SUPPL. 1):426. | EX5 |
| Puglisi Lauren M and Okely Anthony D; Pearson Philip; Vialle Wilma;. 2013. "Understanding the day-to-day lives of obese children and their families.". Family & community health 36(1):42-50. | EX5 |
| Putter Kaila C and Jackson Ben; Thornton Ashleigh L; Willis Claire E; Goh Kong Min Bryce; Beauchamp Mark R; Benjanuvatra Nat; Dimmock James A; Budden Timothy;. 2022. "Perceptions of a family-based lifestyle intervention for children with overweight and obesity: a qualitative study on sustainability, self-regulation, and program optimization.". BMC Public Health 22(1):1-16. | EX3 |
| Rawlins E and Baker G; Maynard M; Harding S;. 2013. "Perceptions of healthy eating and physical activity in an ethnically diverse sample of young children and their parents: the DEAL prevention of obesity study.". Journal of human nutrition and dietetics : the official journal of the British Dietetic Association 26(2):132-44. | EX3 |
| Rich E. 2024. "A New Materialist Analysis of Health and Fitness Social Media, Gender and Body Disaffection: 'You Shouldn't Compare Yourself to Anyone… but Everyone Does'". YOUTH 4(2):700-717. | EX2 |
| Roberts Michele and Pettigrew Simone;. 2013. "Psychosocial influences on children's food consumption.". Psychology & Marketing 30(2):103-120. | EX5 |
| Robertson Wendy and Fleming Joanna; Kamal Atiya; Hamborg Thomas; Khan Kamran A; Griffiths Frances; Stewart-Brown Sarah; Stallard Nigel; Petrou Stavros; Simkiss Douglas; Harrison Elizabeth; Kim Sung Wook; Thorogood Margaret;. 2017. "Randomised controlled trial evaluating the effectiveness and cost-effectiveness of 'Families for Health', a family-based childhood obesity treatment intervention delivered in a community setting for ages 6 to 11 years.". Health technology assessment (Winchester, England) 21(1):1-180. | EX3 |
| Robertson Wendy. 2009. "An evaluation of ‘Families for Health’ : a new family-based intervention for the management of childhood obesity". , . | EX3 |
| Rodgers Rachel F and Wertheim Eleanor H; Damiano Stephanie R; Gregg Karen J; Paxton Susan J;. 2019. "A qualitative, prospective study of children's understanding of weight gain.". The British journal of developmental psychology 37(3):369-381. | EX5 |
| Russell Lucy and Alsop Rachel; Bradshaw Lucy; Clisby Suzanne; Smith Kerry;. 2017. "The state of girls' rights in the UK.". :168. | EX3 |
| Sahota Pinki and Christian Meaghan; Day Rhiannon; Cocks Kim;. 2019. "The feasibility and acceptability of a primary school-based programme targeting diet and physical activity: the PhunkyFoods Programme.". Pilot and feasibility studies 5:152. | EX3 |
| Sarigol Ordin and Yaprak; Karayurt Özgul; Unek Tarkan; Astarciolu Ibrahim;. 2017. "Pediatric liver transplant patients' transition to adulthood: Patient and parent experiences". Nursing and Health Sciences 19:393-399. | EX3 |
| Schell-Busey Natalie and Connell Nadine M; Kahle Lindsay L;. (2017). Weight, Perceptions, and Bullying: What Kind of Pounds Matter?. Journal of Child and Family Studies, 26, pp.2101-2113. | EX1 |
| Sherrington AM and Oakes S; Hunter-Jones P;. 2021. "Advertising healthy eating to young consumers: insights from English and Swedish adolescents". JOURNAL OF MARKETING MANAGEMENT 37(15-16):1624-1655. | EX3 |
| Shrewsbury Vanessa A and Baur Louise A; Nguyen Binh; Steinbeck Katharine;. (2013). Transition to adult care in adolescent obesity: a systematic review and why it is a neglected topic. International journal of obesity (2005), 38, pp.475-479. | EX1 |
| Sides Nicola and Pringle Andy; Newson Lisa;. 2024. "The lived experience of weight loss maintenance in young people.". Health expectations : an international journal of public participation in health care and health policy 27(1):e13955. | EX3 |
| Silver Joanna and Reavey Paula;. 2010. ""He's a good-looking chap aint he?": narrative and visualisations of self in body dysmorphic disorder". Social science & medicine (1982) 70:1641-1647. | EX2 |
| Sommer Rachel and Bullinger Monika; Chaplin John; Do Ju‐ky; Power Mick; Pleil Andreas; Quitmann Julia;. 2017. "Experiencing health-related quality of life in paediatric short stature - a cross-cultural analysis of statements from patients and parents.". Clinical Psychology & Psychotherapy 24(6):1370-1376. | EX5 |
| Spence S and Delve J; Stamp E; Matthews JNS; White M; Adamson AJ;. (2014). Did School Food and Nutrient-Based Standards in England Impact on 11-12Y Olds Nutrient Intake at Lunchtime and in Total Diet? Repeat Cross-Sectional Study. PLOS ONE, 9(11), pp.. | EX1 |
| Staniford Leanne Jane and Breckon Jeff David; Copeland Robert James; Hutchison Andrew;. 2011. "Key stakeholders' perspectives towards childhood obesity treatment: a qualitative study.". Journal of child health care : for professionals working with children in the hospital and community 15(3):230-44. | EX2 |
| Staniford LJ and Copeland RJ; Breckon JD;. 2019. "'What's the point when you only lose a pound?' Reasons for attrition from a multi-component childhood obesity treatment intervention: a qualitative inquiry". QUALITATIVE RESEARCH IN SPORT EXERCISE AND HEALTH 11(3):382-397. | EX3 |
| Stankov Ivana and Olds Timothy; Cargo Margaret;. 2012. "Overweight and obese adolescents: what turns them off physical activity?". International journal of behavioral nutrition and physical activity 9:1-15. | EX2 |
| Su Wei. 2021. "Preschool children's perceptions of other children based on body size". :. | EX5 |
| Swami Viren and Punshon Sarah; Paul Toni-Dee;. (2022). Promoting positive body image in children through theatre: An evaluation of Cinderella: the AWESOME Truth.. Body image, 42, pp.50-57. | EX1 |
| Thompson Claire and Cummins Steven; Brown Tim; Kyle Rosemary;. 2015. "What does it mean to be a 'picky eater'? A qualitative study of food related identities and practices.". Appetite 84:235-9. | EX2 |
| Thyne Maree and Robertson Kirsten; Thomas Tabitha; Ingram Mikaela;. 2016. ""It is amazing how complete is the delusion that beauty is goodness": expectancies associated with tween makeup ownership". International Journal of Consumer Studies 40:543-551. | EX3 |
| Tort-Nasarre G and Pollina-Pocallet M; Suquet Y Ferrer; Bravo M Ortega; Cartagena M Vilafranca; Artigues-Barberà E;. 2023. "Positive body image: a qualitative study on the successful experiences of adolescents, teachers and parents". International Journal of Qualitative Studies on Health and Well-Being 18:. | EX5 |
| Tremblay Line and Lovsin Tanya; Zecevic Cheryl; Larivière Michel;. (2011). Perceptions of self in 3–5-year-old children: A preliminary investigation into the early emergence of body dissatisfaction.. Body Image, 8(3), pp.287-292. | EX1 |
| Trimmer Rachel E and Mandy William P L; Muntoni Francesco; Maresh Kate E;. 2024. "Understanding anxiety experienced by young males with Duchenne muscular dystrophy: a qualitative focus group study.". Neuromuscular disorders : NMD 34:95-104. | EX3 |
| Trimmer Rachel. 2019. "Understanding Anxiety in Duchenne Muscular Dystrophy, Starting with the Perspectives of Boys with the Condition and Parents". , University of London, University College London (United Kingdom). | EX3 |
| Twiddy Maureen and Wilson Inga; Bryant Maria; Rudolf Mary;. 2012. "Lessons learned from a family-focused weight management intervention for obese and overweight children.". Public health nutrition 15(7):1310-7. | EX3 |
| Tylka Tracy L and Calogero Rachel M;. (2010). Fiction, fashion, and function revisited: An introduction to the special issue on gendered body image, Part II.. Sex Roles: A Journal of Research, 63(9-10), pp.601-608. | EX1 |
| Vamos E P and Lewis E; Junghans C; Hrobonova E; Dunsford E; Millett C;. (2016). Community-based pilot intervention to tackle childhood obesity: a whole-system approach.. Public health, 140, pp.109-118. | EX1 |
| van de Pas Kelly. 2024. "Severe obesity in youth". , . | EX3 |
| Visram S and Hall T D; Geddes L;. 2013. "Getting the balance right: qualitative evaluation of a holistic weight management intervention to address childhood obesity.". Journal of public health (Oxford, England) 35(2):246-54. | EX3 |
| Walker Kathleen and Caine-Bish Natalie; Wait Samantha;. 2009. "I like to jump on my trampoline: an analysis of drawings from 8- to 12-year-old children beginning a weight-management program.". Qualitative health research 19(7):907-17. | EX3 |
| Walker M and Mistry B; Amin R; McAdam L;. 2022. "A qualitative exploration of the priorities and experiences of children with Duchenne muscular dystrophy, their parents, and healthcare professionals around weight …". Disability and … :. | EX5 |
| Warburton Victoria E and Beaumont Lee C; Bishop Krystal C. M;. 2022. "Pre-adolescent children's understanding of health and being healthy: a multidimensional perspective from the UK". Health Education 122:519-534. | EX3 |
| Watson Libby A and Baker Martyn C; Chadwick Paul M;. 2016. "Kids just wanna have fun: Children's experiences of a weight management programme.". British journal of health psychology 21(2):407-20. | EX3 |
| Watson Paula M and Dugdill Lindsey; Pickering Katie; Hargreaves Jackie; Staniford Leanne J; Owen Stephanie; Murphy Rebecca C; Knowles Zoe R; Johnson Laura J; Cable N Timothy;. 2021. "Distinguishing factors that influence attendance and behaviour change in family-based treatment of childhood obesity: A qualitative study.". British journal of health psychology 26(1):67-89. | EX3 |
| Watson Paula Mary. 2012. "Feasibility Evaluation and Long-Term Follow Up of a Family-Based Behaviour Change Intervention for Overweight Children (GOALS)". , Liverpool John Moores University (United Kingdom). | EX3 |
| Webb Emma and Teague Bonnie; Farrar Matt; Farrar Victoria; Szinay Dorothy; Chan Li; Ken K; Jackson Ben; Naughton Felix; Wilson Jon; Wylie Sydney; Webb Emma;. 2024. "Views of adolescent patients and their families on the use of digital technology to support health behaviour change in young people under the care of a Complications of Excess Weight service (Preprint)". :. | EX2 |
| Webster Deborah. 2021. "An Exploration of the Online and Offline Social Networks of Post Primary School Pupils in Northern Ireland and Their Relationship with Subjective Wellbeing". , Queen's University Belfast (United Kingdom). | EX2 |
| White W J. 2017. "Novel approaches in adolescent obesity management". , University of London, University College London (United Kingdom). | EX3 |
| Whitelaw S and Smart E; Kopela J; Gibson T; King V;. 2011. "Developing social marketing capacity to address health issues.". Health Education 111(4):319-331. | EX2 |
| Women in Sport and Youth Sport Trust;. 2015. "The tipping point: confidence and attitudes in seven and eight year old girls - provisional findings". :9. | EX3 |
| Yeatts Paul E and Martin Scott B; Farren Gene L;. (2019). Adolescents’ psychological well-being and their perceptions of parental encouragement to control weight. Journal of Family Studies, 27, pp.607-620. | EX1 |
| Zigler C K and Ardalan K; Hernandez A; Caliendo A E; Magee K E; Terry M A; Mann C M; Torok K S;. 2020. "Exploring the impact of paediatric localized scleroderma on health-related quality of life: focus groups with youth and caregivers.". The British journal of dermatology 183(4):692-701. | EX3 |
