# Supplementary material for: Children’s views of obesity, body size and weight: systematic review of UK qualitative evidence
Source: J Epidemiol Community Health. 2026 Jan 27;80(6):e225045. doi: 10.1136/jech-2025-225045 (PMC13217130; doi:10.1136/jech-2025-225045)
Supplement: online supplemental file 5 [file jech-80-6-s005.docx]

***Children’s views of obesity, body size and weight: Systematic review of UK qualitative evidence***

Appendix: Coding frame

- Impacts of overweight / obesity / weight gain / loss
  - Physical abilities
  - Health impacts
  - Appearance / attractiveness
  - Social impacts (incl. bullying /teasing)
  - Emotional impacts
  - Reactions from others
  - Lack of impact
- Personal experiences / desires for oneself
  - Motivations for / experiences of attempted weight loss/gain
  - Disordered eating / fear of fatness
  - Dis/satisfaction with own body shape/weight
  - Body ideals
    - Media / social media
    - Comparison with peers
    - Rejection of thin ideal
  - Experiences of weight monitoring
- Attitudes of others / 'fat talk'
  - Teachers
  - Parents
  - Peers
  - Other / general
- Differences between groups
  - Gender
  - Ethnicity
  - SES
  - Age
- Negative views / stereotypes of overweight people
  - Lazy
  - Unhealthy
  - Unhappy / want to change
  - Unpopular
  - Unfit / physical ability
  - Greedy
  - Other
- Perceived causes of overweight / weight loss
  - Diet / ‘healthy’ foods
  - Physical activity
  - Social / environmental factors
  - Whose responsibility?
  - ‘Naturally’ fat/thin
  - Other
- Experiences of overweight children
  - Views of own body shape
  - Experiences of losing weight
  - Food behaviours / attitudes
  - Bullying, attitudes of others
  - Participation in activities
- Views relating to height
