# Supplementary material for: Children’s views of obesity, body size and weight: systematic review of UK qualitative evidence
Source: J Epidemiol Community Health. 2026 Jan 27;80(6):e225045. doi: 10.1136/jech-2025-225045 (PMC13217130; doi:10.1136/jech-2025-225045)
Supplement: online supplemental file 6 [file jech-80-6-s006.docx]

***Children’s views of obesity, body size and weight: Systematic review of UK qualitative evidence***

Appendix: Further detail on study characteristics

| **First author, reference(s)** | **Study focus** | **Data collection method** | **Analytical approach** | **Sampling approach** | **Sample size** | **Age** | **% female** | **Other information on sample/context** |
| --- | --- | --- | --- | --- | --- | --- | --- | --- |
| Baxter [14,15] | Understandings of weight change | Individual interviews (based on a picture storybook) | Thematic analysis; coding based on research aims | Sampling of schools NR.  All Reception / Year 1 children with parental consent obtained (100/220). | 100 | 4-6 | 38% | – |
| Bell [16] | Views of "fitspiration" content on social media | Focus groups | Reflexive thematic coding; inductive development of themes | Sampling of school NR. Implicitly, all pupils in selected year group invited to participate; participation rate 100% | 77 | 12-13 | 35% | Higher-SES school |
| Blood [17] | Experiences of school-based height and weight measurement | Individual interviews and focus groups | Thematic analysis | Sampling of school NR.  Implicitly, all year 6 pupils invited. | 12 | 10-11 | 75% | 75% BME sample |
| Bromfield [18] | Implementation of National Healthy Schools Programme relating to obesity | Focus group | Thematic analysis | Schools sampled from a 'services cluster' on the basis of existing relationships and because the cluster had an obesity strategy.  Children selected by school staff. | 6 (for the relevant section of the study for this review) | 9-11 | NR | Lower-SES area |
| Charsley [19,20] | Perceptions of fatness and other physical differences | Individual interviews (based on picture cards) | Thematic coding | Sampling of school NR (areas with different levels of affluence).  All children in Reception, Year 1 and Year 2 with parental consent (90/450), unless they did not consent or complete the task (n=5) | 85 | 4-7 | 49% | 39% BME sample |
| Clark [21,22] | Girls' experiences of sport and physical activity | Repeated individual and group interviews, observations at school PE lessons and sports clubs | Inductive, emergent coding | NR | 16 | 10-13 | 100% | – |
| Conway [23] | Views of food labelling and the NCMP | Focus groups | Framework analysis | Schools sampled for diversity of SES (% children eligible for free school meals).  Teachers selected children who had parental consent and were willing to participate. | 80 | 9-13 | 64% | 61% BME, most lower-SES sample |
| Cowley [24] | Attitudes to PE and physical activity | Focus groups | Thematic analysis | Purposive sampling of schools for diversity of socioeconomic and school characteristics. Sampling of individuals NR. | 39 | 12-13 | 44% | Medium-SES school |
| Dearing [25] | Pro-social behavioural intentions towards peers with obesity | Individual interviews (based on a storybook) | Thematic analysis without prior framework | Purposive sampling of schools for being average in terms of % pupils receiving pupil premium; 2 schools recruited from 35 approached; 2 others recruited through contacts.  Children in selected year groups invited to participate, with parental consent (29% response rate) | 72 | 4-6 | 35% | Mix of settings |
| Fairbrother [26] | Perceptions of food and relationship between food and health | Repeated group interviews, individual interviews | Thematic networks analysis | Purposive sampling of two schools for socioeconomic diversity, one Year 5 class nominated by school staff.  For group interviews, all children in a Year 5 class (which ensured a gender balance) were invited; participation rate NR. For individual interviews, theoretical sampling based on findings from group interviews. | 53 | 9-10 | NR | Mix of settings |
| Fielden [27] | Understandings of obesity and links to diet and physical activity | Focus groups | Thematic mapping | Sampling of school NR.  Children from Reception and Year 6, sampled for gender balance and ethnic diversity, through liaison with school staff. | 12 | 4-5, 10-11 | NR | – |
| Gemmell [28] | Overweight children's perceptions and experiences relating to body size | Individual interviews | Interpretive Phenomenological Analysis. Initial inductive thematic coding followed by deeper coding of detail. | Recruited from a community-based health promotion intervention for children with overweight (response rate NR but ‘below that anticipated’). | 6 | 8-12 | 67% | All overweight |
| Gillison [29] | Development of guidance for parents on talking to children about weight | Individual interviews via zoom, with illustrated story cards | NR | Convenience sampling – adverts in local media and online. Children eligible regardless of weight status. | 11 | 9-11 | 63% | – |
| Goldthorpe [30] | Views about responsibility for health | Focus groups | Interpretive Phenomenological Analysis | Schools participating in a Healthy Schools programme were sampled for diversity in stage of implementation in the programme and in geographical area, and further selected to be representative of population demographics.  Convenience sampling of children by teachers; aimed for gender balance and those having school dinners vs packed lunch. Analysis focused on 2 of 14 focus groups, chosen for richness of data. | 20 | 8-10 | 50% | Lower-SES area; 56% BME sample |
| Hall [31] | Experiences of children with overweight; experiences of weight-based victimisation | Group interviews | Contextualist thematic analysis | Sampling of school NR.  Children in selected year group with parental consent (32/120), who were not absent on day of data collection (n=3). | 29 | 9-11 | 52% | Higher-SES area; mostly White sample |
| Harrold [32] | Obesity stigma | Individual interviews, based on illustrated story | Framework analysis | Sampling of schools NR (4/15 approached agreed to participate).  Children in Reception or Year 1, with parental consent (131/299) except those with learning and speech impairments (n=1) | 130 | 4-7 | 54% | Lower-SES area; mostly White sample |
| Herbert [33] | Views of weight monitoring | Focus groups, and thoughts written down and put in ‘secret box’ | Thematic analysis | All schools in selected area invited, as well as personal contacts (response rate 11/285, of which 4 recruited sufficient children to participate).  Children in selected year group with parental consent (response rate NR but ‘low’) | 48 | 8-12 | 48% | Mixed-SES schools |
| Hooper [34] | Conceptions of health relating to physical education | Repeated participatory focus group | Foucauldian discourse analysis. | Schools sampled based on prior survey, to select those addressing healthy lifestyles.  Children selected by stratified sampling (gender, ethnicity, SEN, English as additional language, SES), with opt-out parental consent (143/150. | 143 [?] | 11-12 | NR | Mix of settings |
| Kamal [35] | Views about determinants of obesity and compensatory reasoning about health behaviours | Individual interviews and focus groups | Content analysis, using a priori framework (for interviews), and thematic analysis using a critical realist approach (focus groups) | Sampling of schools NR (2 schools for individual interviews; 1 of these was also used for focus group).  Children sampled from Years 1, 2, 4 and 5, if had conversational English and (for interviews) ability to draw pictures (as advised by school staff). | 48 (interviews);  35 (focus groups) | 5-10 | 55% | Higher-SES, mostly White school |
| Kesten [36] | Influences on girls' obesity-related health behaviours | Focus groups | Thematic coding | Stratified random sampling of schools (size and area-level SES).  All Year 3-6 children invited. Sample size guided by theoretical saturation. | 56 | 6-11 | 100% | Mix of settings |
| Kumari [37] | Views of young people with overweight | Individual interviews | Grounded theory approach | Participants recruited in person from a weight management programme | 11 | 11-13 | 36% | 54% BME sample. All overweight |
| Lewis [38] | Views of physical activity in children with overweight | Individual interviews | Thematic analysis with a priori framework | Participants recruited from community-based weight management programme. Purposive sampling (for diversity in terms of age, ethnicity and area-level SES) (58/290) | 58 | 6-16 | 50% | Lower-SES area. All overweight |
| Mansfield [39] | Views of childhood obesity | Interviews | Thematic analysis | Purposive sampling of school for low SES.  All children in Year 5 with parental consent (9/30) | 8 [?] | 9-10 | 37% | Lower-SES area |
| Miller [40] | Views on prevention of eating disorders | Focus groups | Framework analysis | Recruited through Guiding units, if parental and participant consent and no diagnosed or possible eating disorder. | 22 | 10-14 | 100% | – |
| Monaghan [41] | Views of body size and health | Focus groups | Feminist poststructural discourse analysis | Sampling of school NR  Sampling of participants NR. | 24 | 12-13 | 100% | Most White |
| Murphy [42] | Views of health, diet, physical activity and weight | Individual interviews | Framework analysis | Schools sampled to target high BME and/or low SES schools.  All children in selected year groups invited and recruited in order of response, with additional sampling to prioritise BME participants (26/210) | 26 | 9-10 | 58% | 73% BME, most lower-SES sample |
| Newson [43] | Experiences of children with obesity; views of a weight management programme | Individual interviews, plus scrapbook activity | Reflexive thematic analysis, with inductive, iterative coding | Children living with obesity sampled from community-based weight management programmes. | 34 | 7-13 | 56% | All overweight |
| Nnyanzi [44,45] | Impact and implementation of the NCMP | Individual interviews | Thematic analysis | Proportionate stratified random sampling of schools (for diversity of SES).  Participants sampled purposively for diversity in area-level SES, from the quantitative sample. | 21 | 10-11 | 67% | All white, most lower-SES sample |
| Ogden [46] | Choices about food | Individual interviews | Inductive thematic analysis | School sampling NR.  All children in selected year group, with parental consent (27 of approximately 80) | 27 | 9-10 | 63% | All White sample |
| Paddock [47,48] | Use of social media | Focus groups | Reflexive thematic analysis | School sampling NR.  Participant sampling NR but assume all students in a class, since focus groups took place as part of a citizenship lesson at school. | 64 | 11-14 | 52% | Mostly White sample; lower-SES area |
| Palmer [49] | Understandings and experiences of the body | Observations; group interviews | Thematic analysis | Schools sampled for diversity in SES, cultural context and state vs private.  Participants volunteered following presentations to class (all those in selected year groups), following parental consent (39/92) | 39 | 9-10 | 59% | Mix of settings |
| Rich [50–53] | [Varied aims in different reports] | Interviews in pairs, or groups of 3 or 4. | NR | Criterion sampling strategy for selecting schools for diversity in "social, cultural and policy contexts".  Individual participants selected by teachers.[50] | 90[50] | 9-16 | NR | – |
| Willett [54] | Girls' views about fashion and digital media | Workshops, with recorded conversations and group interviews (groups of 2-4) | NR | School sampling NR.  Participants sampling NR but selected by school with the aim of being representative of the school population. | 26 | 12-13 | 100% | Lower-SES, 75% BME school |
| Windram-Geddes [55,56] | Girls' experiences of PE and physical activity | Focus groups (groups of 2-4 in primary school, groups of 4-8 in secondary school) and, in secondary school, one individual interview | Thematic coding | Schools sampled for diversity of location and demographics.  Sampling in primary school through ‘Keep Active Club’, further details NR. In secondary schools all girls in selected year groups were invited to participate. | For primary approx. n=12; for secondary n=107 | 10-14 | 100% | – |
